# Novel Benzimidazole–Oxadiazole Derivatives as Anticancer Agents with VEGFR2 Inhibitory Activity: Design, Synthesis, In Vitro Anticancer Evaluation, and In Silico Studies

**DOI:** 10.1021/acsomega.4c08885

**Published:** 2025-02-14

**Authors:** Ulviye Acar Çevik, Ismail Celik, Şennur Görgülü, Zeynep Deniz Şahin İnan, Hayrani Eren Bostancı, Arzu Karayel, Yusuf Özkay, Zafer Asım Kaplancıklı

**Affiliations:** † Department of Pharmaceutical Chemistry, Faculty of Pharmacy, Anadolu University, Eskişehir 26470, Turkey; ‡ Department of Pharmaceutical Chemistry, Faculty of Pharmacy, Erciyes University, Kayseri 38039, Turkey; § Medicinal Plant, Drug and Scientific Research and Application Center (AUBIBAM), Anadolu University, Eskişehir 26470, Turkey; ∥ Department of Histology and Embryology, Sivas Cumhuriyet University, Sivas 58140, Turkey; ⊥ Department of Biochemistry, Faculty of Pharmacy, Sivas Cumhuriyet University, Sivas 58140, Turkey; # Department of Physics, Faculty of Arts and Science, Hitit University, Çorum 19030, Turkey

## Abstract

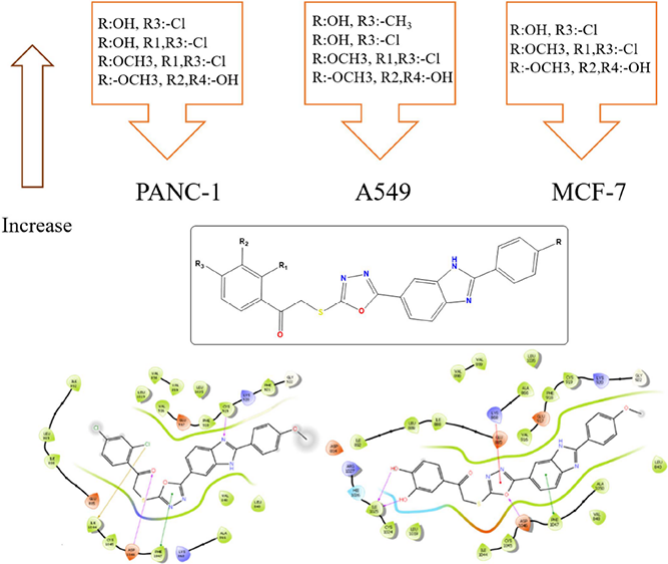

The aim of this research is the synthesis of benzimidazole-1,3,4-oxadiazole derivatives that could be potential anticancer leads inhibiting VEGFR2. The compounds were evaluated for their cytotoxicity against cancer cell lines PANC-1, MCF-7, and A549 using the MTT assay. Two different normal cell lines (hTERT-HPNE and CCD-19Lu) were used to calculate the selectivity indices of the compounds. Compound **4r** showed the highest anticancer activities, with IC_50_ = 5.5, 0.3, and 0.5 μM against the PANC-1, A549, and MCF-7 cell lines, respectively. Also, compounds **4r** and **4s** were further evaluated for their inhibitory activity against VEGFR2. VGFRA immunolocalizations of compounds **4r** and **4s** were visualized by the VEGFA immunofluorescent staining method. Molecular docking studies have proven that, as in sorafenib, compounds **4r** and **4s** show hydrogen bond formation with Asp1046 and Cys919 and hydrophobic interactions with other active site amino acids. Molecular dynamics simulations were carried out for compounds **4r** and **4s** to examine the stability and behavior of the protein–ligand complex obtained from molecular docking under in silico physiological conditions. An in silico ADME investigation was undertaken to confirm the druglikeness of the synthesized compounds. Furthermore, the stable geometries of the ligands were determined through the application of density functional theory (DFT). The optimized geometries were confirmed to correspond to true minima, as no imaginary frequencies were observed in the vibration frequency survey. The rotations of the thio and benzimidazole groups with respect to the 1,3,4-oxadiazole rings are 180 deg, and the molecules are planar.

## Introduction

Despite improvements in diagnosis and treatments for many cancers, there are still unmet needs for treatment, notably for pancreatic cancer. Researchers have conducted extensive research on the development of novel anticancer drugs that minimally impact healthy cells and reduce the risk of subsequent cancer.^1^ Given the findings that vascular endothelial growth factor (VEGF) can promote the proliferation and migration of vascular endothelial cells and induce the formation of blood vessels, the VEGF receptor (VEGFR) has recently become a prominent anticancer drug target.^2^ Since VEGFA is the most important mediator that induces angiogenesis through VEGFR2, antiangiogenic agents available today mostly target VEGFA or its receptor called VEGFR2.^3^ VEGFR2 is a tyrosine kinase receptor, which exerts its activity by binding to VEGF. The signal transduction networks initiated by VEGFA/VEGFR2, the most prominent ligand–receptor complex in the VEGF system, lead to endothelial cell proliferation, migration, survival, and new vessel formation involved in angiogenesis. VEGFR2 inhibitors, also known as kinase insert domain receptor (KDR) inhibitors, are tyrosine kinase receptor inhibitors that reduce angiogenesis, leading to anticancer activity. VEGFR2 inhibitors targeting tumor angiogenic pathways have been widely used in clinical cancer treatment. Therefore, one appealing strategy for developing novel candidates for selective anticancer drugs is targeting VEGFR2.^4−10^

**Figure 1:**
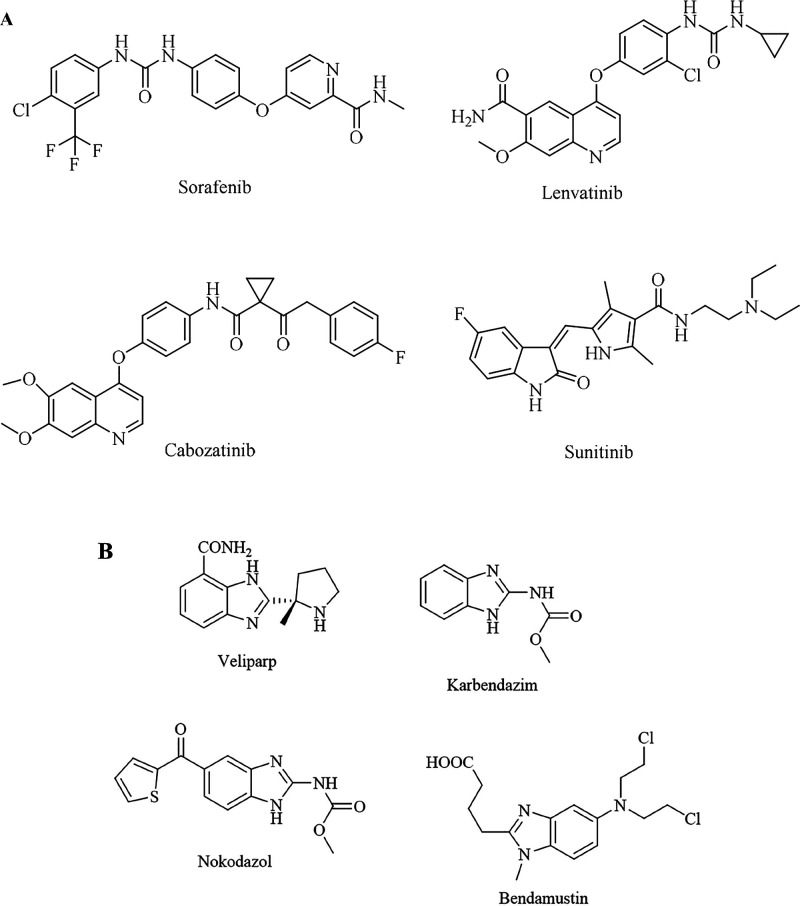
(a) FDA approved some VEGFR2 inhibitors. (b) Anticancer drugs containing benzimidazole structure.

Sorafenib, cabozatinib, lenvatinib, and sunitinib are VEGFR2 inhibitors approved by the Food and Drug Administration (FDA) (Figure 1a).^11−14^

Benzimidazole is an important part of many drugs, such as veliparib, bendamustine, nocodazole, and carbendazim (Figure 1b).^15−18^ Researchers have investigated benzimidazole compounds for their numerous biological actions, including antibacterial, anti-inflammatory, antiallergic, antioxidant, antitubercular, anthelmintic, antimalarial, and anticancer qualities.^19−26^

In the context of structure–activity studies, it is essential to determine the correct molecular structures. Density functional theory (DFT) is one of the most prevalent methodologies currently employed for elucidating the structures of quantum many-body systems, including atoms, molecules, and solids. It is based on the solution of the Schrödinger wave equation. This theory accepts electron density as the fundamental variable instead of the many-particle wave function in electronic structure calculations and uses the relationship between the electron density and the energy of a system. This method is based on the Hohenberg–Kohn^27^ and Kohn–Sham^28^ theorems. The loop in the calculations can be summarized as follows: the Kohn–Sham equations are solved numerically by means of a procedure known as the self-consistency loop, which is repeated until a solution is obtained. The process commences with an initial estimation of the electron density and effective potential, which are then used to create the exchange–correlation potentials. A new density is established by solving the Kohn–Sham equations. Consequently, at each iteration of the self-consistency loop, the energy is calculated, and this process continues until the ground state energy is reached.

Based on the importance of benzimidazole potency, in our present work, we aimed to explore the potential anticancer activity of newly designed 5-(2-(4-substitutedphenyl)-1*H*-benzimidazol-6-yl)-1,3,4-oxadiazole-2-thiol derivatives. To identify the pharmacological potency of all the newly synthesized series of benzimidazol-5-(2-(4-substitutedphenyl)-1,3,4-oxadiazole derivatives, we screened the compounds for in vitro anticancer activities against PANC-1, MCF-7, and A549 cancer cell lines using the MTT assay. Computational in silico methods, such as molecular docking and molecular dynamics simulations, are indispensable in drug discovery, as they provide insights into molecular interactions, predict biological activity, and guide experimental studies efficiently. An in silico study was performed to understand the interactions at the active site of the protein and benzimidazole derivatives VEGFR2 tyrosine kinase receptors and to correlate the compound’s activity. Furthermore, a computational ADME analysis was conducted to verify the drug-like properties of the synthesized compounds. In addition, the density functional theory (DFT) method was used to calculate the electronic properties of 16 newly synthesized compounds in order to identify which substituent group is more stable and chemically active.

## Results and Discussion

### Chemistry

**Figure 2:**
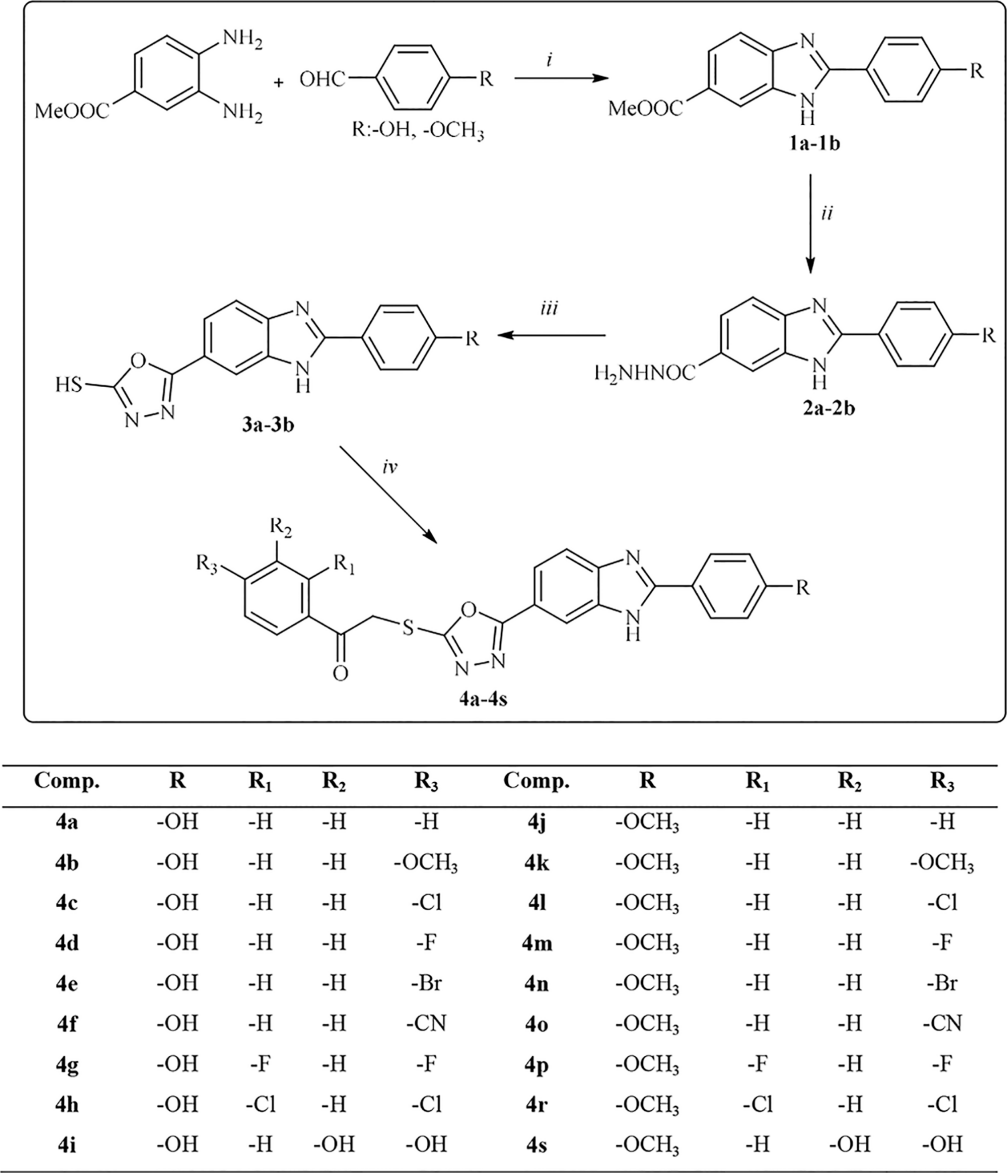
Synthesis procedure for obtaining target compounds (**4a–4s**). Reagents and conditions: (i) 4-hydroxy/methoxy benzaldehyde, Na_2_S_2_O_5_, DMF, MW, 10 min; (ii) 2-(4-substitutedphenyl)-1*H*-benzimidazole-6-carbohydrazide, NH_2_NH_2_·H_2_O, MW, 10 min; (iii) CS_2_, NaOH, EtOH, 6 h; and (iv) phenacyl bromide derivatives, K_2_CO_3_, acetone, 12 h.

In this study, 18 new compounds bearing the benzimidazole-oxadiazole structure were synthesized, as exhibited in Figure 2, and their structures were confirmed by spectroscopic methods (^1^H NMR, ^13^C NMR, and HRMS). In the first step of the synthesis studies, 4-hydroxy/methoxy benzaldehyde and sodium metabisulfite were reacted in dimethylformamide under microwave irradiation, and because of the condensation reaction of the resulting benzaldehyde sodium metabisulfite adduct and 3,4-diamino benzoate under microwave irradiation, a compound **1** derivative was obtained. In the next step, compound **1** was treated with hydrazine hydrate under microwave irradiation to obtain 2-(4-substitutedphenyl)-1*H*-benzimidazole-6-carbohydrazide (**2**). The synthesized hydrazide derivative (**2**) was dissolved in ethanol; NaOH and CS_2_ were added; and the oxadiazole ring was obtained. In the last step, compound **3** and phenacyl bromide derivatives were reacted in acetone to reach the target products.

### In Vitro Studies

#### Anticancer Activity

The synthesized compounds **4a–4s** were screened for their in vitro anticancer activity via the standard MTT assay using three human tumor cell lines, namely, the human lung adenocarcinoma cell line (A549), the human breast cancer cell line (MCF-7), and the human pancreas cell line (PANC-1). Cisplatin was used as a reference drug. The results, expressed as IC_50_ values of the final compounds, are summarized in Table 1. These compounds showed better cytotoxicity compared to cisplatin on PANC-1 cells, as some compounds **4c**, **4h**, **4r**, and **4s** exhibited IC_50_ values of 17.7, 26.7, 5.5, and 6.7 μM, respectively. Among these compounds, compounds **4r** and **4s** have the highest potency. When the structures of the compounds are evaluated, it is seen that the compounds are derived from two different sources. There are hydroxyl and methoxy substituents on the phenyl ring attached to the benzimidazole. It has also been derivatized using different groups on the phenyl ring close to oxadiazole. When the activities in the PANC-1 cell line are evaluated, it is seen that the activity of compounds bearing the 2,4-dichloro substituent is especially high. In addition, it is observed that the activity increases significantly with the replacement of the hydroxyl group with the methoxy group in the phenyl ring attached to the benzimidazole. It was determined that the activity of the 3,4-dihydroxy group increased significantly with the presence of the methoxy group in the phenyl ring attached to the benzimidazole.

**Table 1:** IC_50_ Values (μM) of the Compounds against hTERT-HPNE, PANC-1, A549, HepG2, CCD-19Lu, and MCF-7 Cell Lines

	cell lines				
comp.	hTERT-HPNE	PANC-1	A549	CCD-19Lu	MCF-7
**4a**	26.2	42.2	63.2	46.1	39.5
**4b**	86.8	33.1	5.1	40.3	17.4
**4c**	21.5	17.7	6.3	16.3	6.1
**4d**	6.8	33.3	10.3	5.2	10.1
**4e**	11.3	83.8	96.1	95.3	95.2
**4f**	74.9	60.4	50.7	68.8	45.6
**4g**	17.1	36.6	16.8	31.8	30.7
**4h**	32.9	26.7	14.6	30.3	15.7
**4i**	10.1	23.8	21.1	13.9	16.5
**4j**	93.6	95.7	99.1	95.2	53.7
**4k**	29.3	82.0	74.7	90.4	50.2
**4l**	19.5	42.1	49.1	47.8	22.6
**4m**	28.6	41.1	25.3	20.2	36.9
**4n**	36.2	29.5	19.6	36.8	28.9
**4o**	64.2	70.9	65.2	48.4	49.2
**4p**	20.4	32.6	14.9	14.7	37.1
**4r**	10.9	5.5	0.3	0.8	0.5
**4s**	38.3	6.6	1.6	1.3	1.2
cisplatin	30.6	28.8	19.7	22.6	14.7

The compounds were generally found to be more effective against the A549 cell line. These compounds showed better cytotoxicity compared to cisplatin on A549 cells, as compounds **4b**, **4c**, **4g**, **4h**, **4n**, **4p**, **4r**, and **4s** exhibited IC_50_ values range of 0.3 μM-19.6 μM. Again, it was determined that the most effective compounds in the series were **4r** and **4s**, with IC_50_ values of 0.3 and 1.6 μM, respectively.

**Figure 3:**
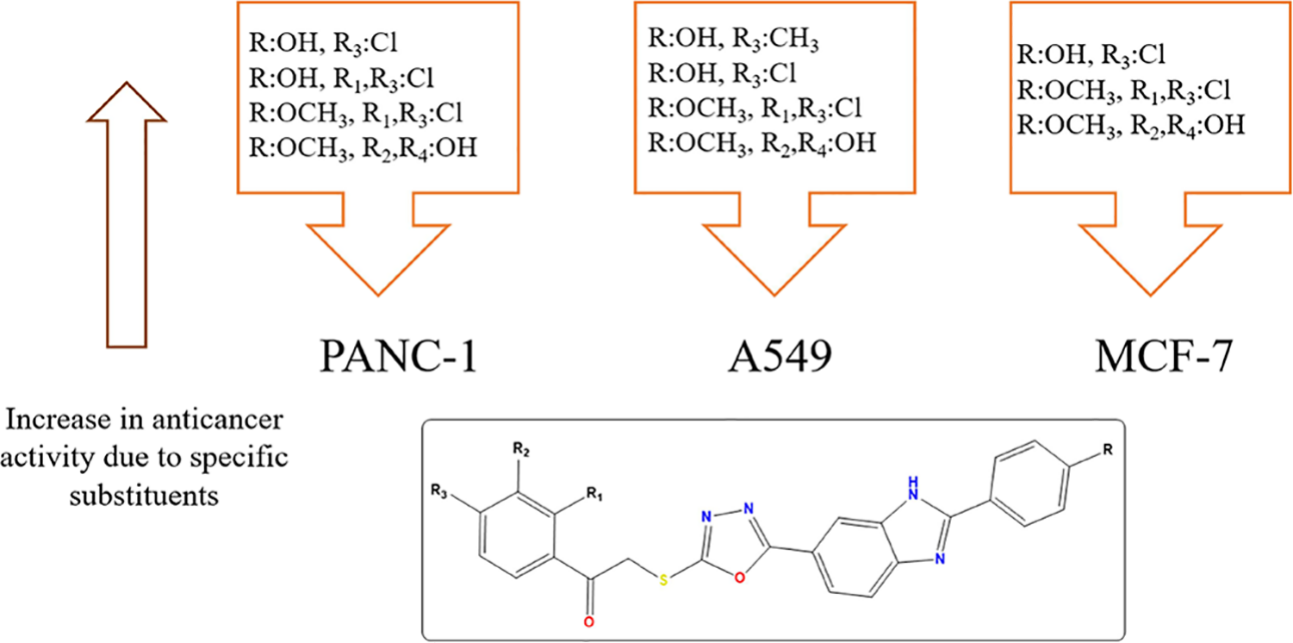
SAR study of compounds **4a–4s**.

The SAR study of compounds **4a–4s** is shown in Figure 3. Compounds **4c**, **4r**, and **4s** were the most potent cytotoxic compounds on the MCF-7 cell line, with IC_50_ values of 6.1, 0.5, and 1.2 μM, respectively. Compound **4r**, with a methoxy group and a 2,4-dichloro group, showed strong activity (0.3–5.5 μM) against all three cell lines. Compound **4s**, with a methoxy group and a 3,4-dihydroxy group, showed strong activity (1.2–6.6 μM) against all three cell lines.

#### Selectivity Index (SI)

By comparing the compounds’ cytotoxic effects against tumor cell lines and normal cell lines, the drug safety parameter for the anticancer activity of the compounds was evaluated, thereby confirming their cytoprotective qualities. In this study, normal human lung (CCD-19Lu) and normal human pancreas (hTERT-HPNE) cell lines were used as controls. The selectivity index (SI) calculated for the compounds tested scales the IC_50_ values of the compounds against various tumor cell lines with the IC_50_ values against normal cell lines (Table 2). Compound **4s** exhibited a moderate selectivity index, particularly against pancreatic cancer, with an SI value of 5.80. This suggests that **4s** is significantly more cytotoxic to pancreatic cancer cells compared with normal pancreatic cells, highlighting its potential as a targeted therapeutic agent for pancreatic cancer. However, its selectivity against lung cancer was notably low, with an SI value of 0.81, indicating limited specificity for lung cancer cells over normal lung cells. Conversely, compound **4r** demonstrated a higher selectivity index for lung cancer, with an SI value of 2.66, suggesting a preferential cytotoxic effect on lung cancer cells compared with normal lung cells. This highlights the potential of **4r** as a selective therapeutic agent for lung cancer. In comparison, the selectivity index of **4r** for pancreatic cancer was lower, at 1.98, indicating a reduced, yet still notable, specificity for pancreatic cancer cells. These findings suggest that compound **4s** is a promising candidate for pancreatic cancer therapy due to its moderate selectivity for pancreatic cancer cells, while compound **4r** shows promise for lung cancer treatment due to its moderate selectivity for lung cancer cells. The differential selectivity indices underscore the importance of targeted therapeutic strategies and the potential for these compounds to provide selective anticancer effects with reduced toxicity to normal cells.

**Table 2:** Selectivity Indices of Compounds **4r** and **4s**

compounds	(PANC-1)^a^	(A549)^b^
**4r**	1.98	2.66
**4s**	5.80	0.81

a SI = cytotoxicity against hTERT-HPNE cells/cytotoxicity against PANC-1 cell line.

b SI = cytotoxicity against CCD-19Lu cells/cytotoxicity against A549 cell line.

#### VEGFA Immunofluorescent Staining

**Figure 4:**
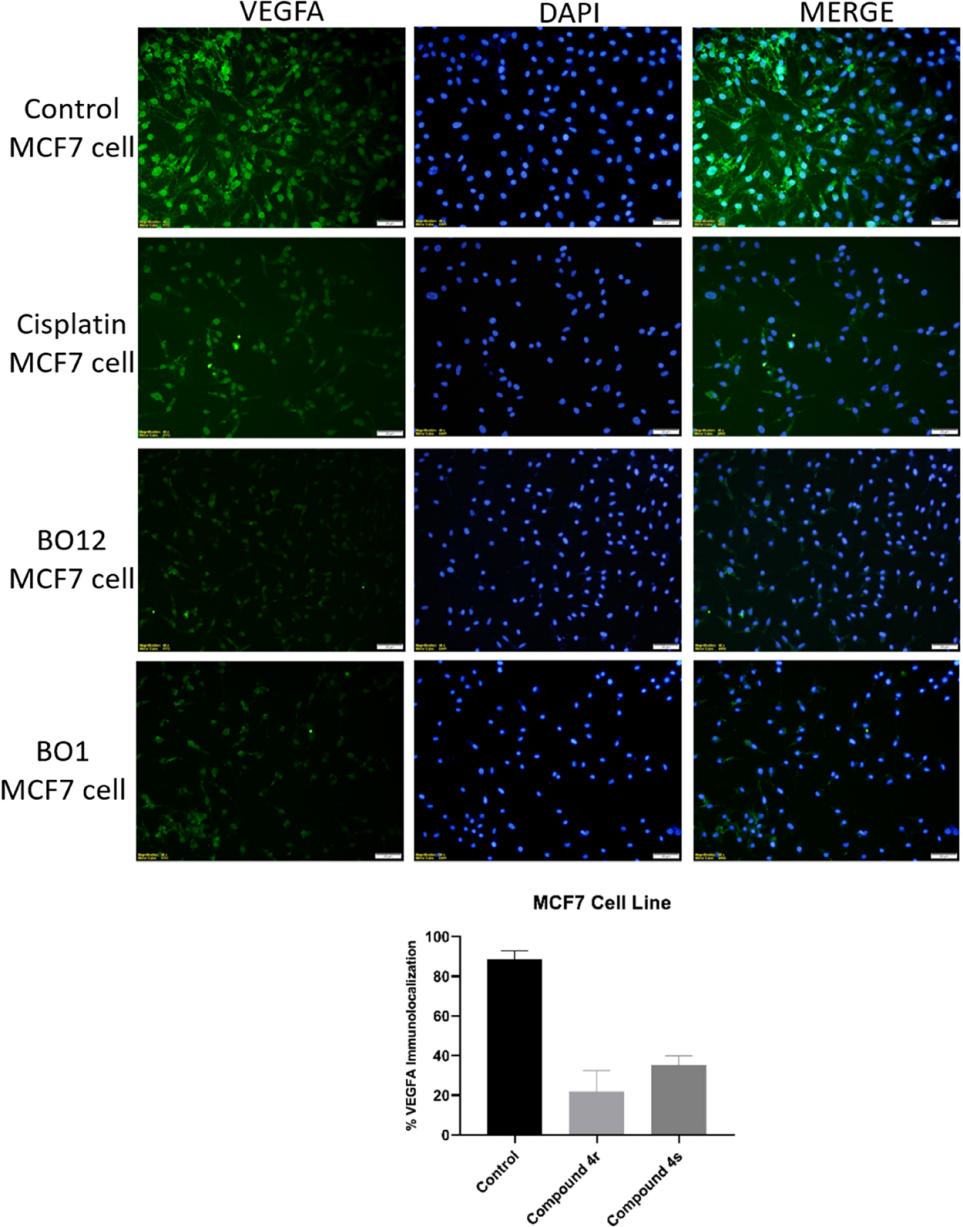
Treatment applied to MCF-7 breast cancer cell line compounds **4r** (BO12), **4s** (BO1), and the untreated control group are green with the anti-VEGFA antibody, cell nucleus is blue with DAPI staining, and merge images are given to understand the difference (X40 Magnification, Alexa Fluor 488; green, DAPI; blue). After treatment with MCF-7 breast cancer cell line compounds **4r** (BO12), **4s** (BO1) and untreated control group, the immunolocalization of the anti-VEGFA antibody among the groups was determined by semiquantitative scoring and graphed.

An endogenous angiogenic growth factor called VEGFA primarily promotes the migration and proliferation of vascular endothelial cells.^2^ Fluorescent staining degrees of the MCF-7 cancer cell line, marked with an anti-VEGFA antibody and immunofluorescence technique, were compared under light microscopy (Figure 4). Accordingly, compounds **4r** and **4s** were applied to the cell lines and maintained for 24 h. The VEGFA immunolocalization of compounds **4r** and **4s** in MCF-7 breast cancer cells was compared with the control. Accordingly, while the highest VEGFA immunolocalization was observed in the control group, less VEGFA immunolocalization was observed in compounds **4r** and **4s**. Five different areas were selected for each group. All cell nuclei seen in the selected areas are compared with those stained VEGFA positive. When evaluated as a percentage, much less VGFRA immunolocalization was observed for compounds **4r** and **4s** compared to that of the control.

#### Assessment of VEGFR2 Inhibition

The comparative analysis of compounds **4r**, **4s**, and sorafenib against VEGFR2 inhibition, as illustrated in Table 3, demonstrates distinct differences in their efficacy. Sorafenib exhibits the highest potency with an IC_50_ value of 0.312 ± 0.0112 μM, indicating its strong inhibitory effect at lower concentrations. Compound **4r** follows with an IC_50_ value of 0.418 ± 0.021 μM, showing a slightly reduced but still significant inhibitory potential compared to that of sorafenib. Compound **4s**, with an IC_50_ value of 0.502 ± 0.028 μM, is the least potent among the tested compounds yet maintains a level of efficacy that suggests potential therapeutic relevance. The ranking of inhibitory efficacy is as follows: sorafenib > **4r** > **4s**. Despite **4r** and **4s** having higher IC_50_ values than sorafenib, their values remain below 1 μM, indicating that both compounds are still effective inhibitors of VEGFR2. Given sorafenib’s established clinical use, these findings suggest that **4r** and **4s** could serve as promising candidates for further development as VEGFR2 inhibitors. As depicted in Table 3, the error bars represent the standard deviations, highlighting the reliability and reproducibility of the data. The visual representation underscores the relative potencies of the compounds, clearly showing the superior efficacy of sorafenib, followed by **4r** and then **4s**.

**Table 3:** Inhibitory Effects of Compounds **4r** and **4s** against VEGFR2

compounds	IC_50_ (μM)
**4r**	0.418 ± 0.021
**4s**	0.502 ± 0.028
sorafenib	0.312 ± 0.0112

### In Silico Studies

#### Molecular Docking

**Figure 5:**
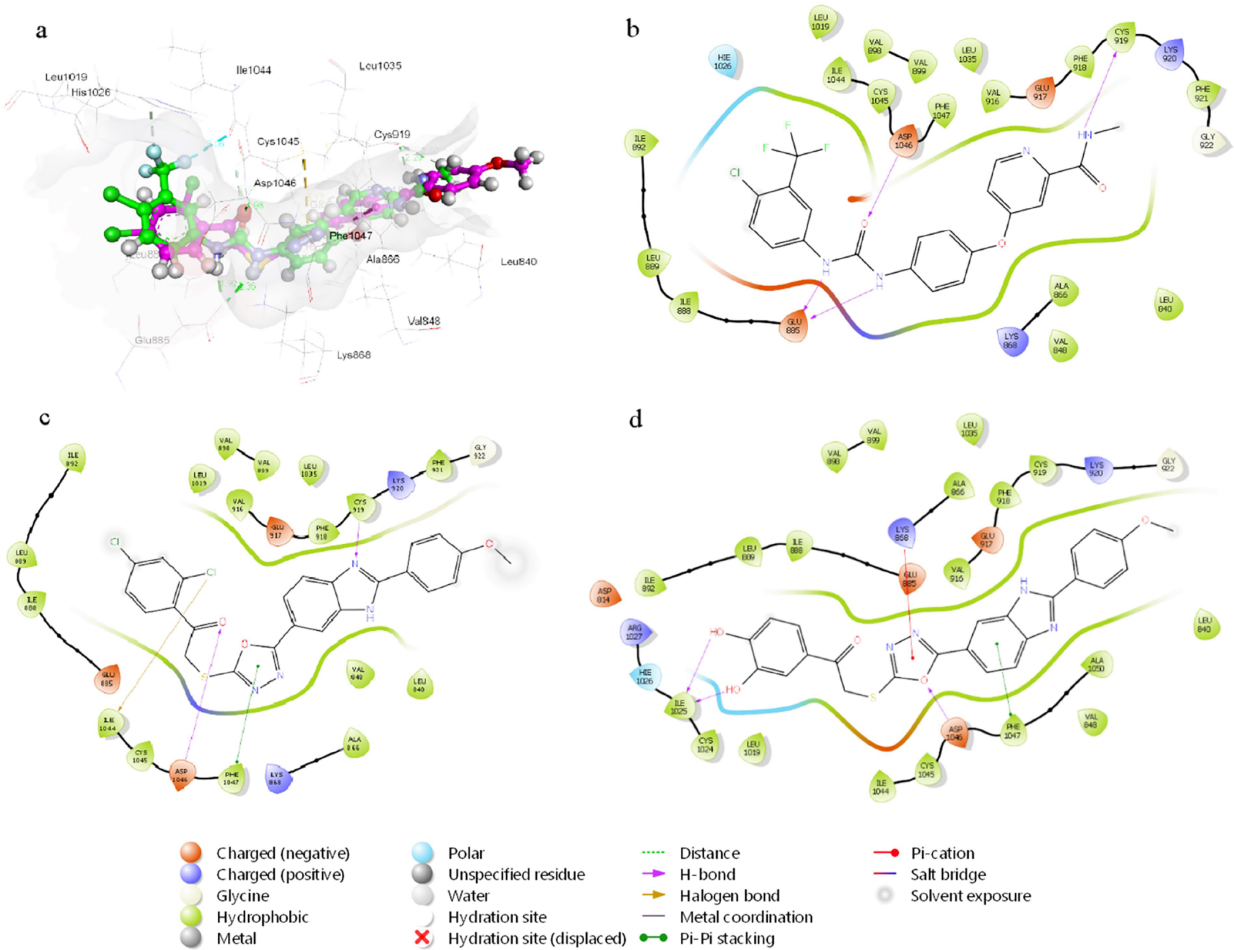
(a) Superimposed binding poses of compound **4r** and sorafenib in the VEGFR2 active site, (b) two-dimensional interaction diagram of sorafenib, (c) two-dimensional interaction diagram of compound **4r**, and (d) two-dimensional interaction diagram of compound **4s**.

The unique pharmacophore group centered on the benzimidazole core was designed as a potential VEGFR2 inhibitor using computer-aided design. For VEGFR2 inhibition, the formation of hydrogen bonds with Asp1046, Glu885, and Cys919, as well as interactions with active site amino acids such as Phe1047, Ile1044, and Cys1045, similar to those in sorafenib, is significant. Specifically, the carbonyl group of sorafenib forms hydrogen bonds with Asp1046, while its oxadiazole oxygen interacts with Cys919. Similarly, compound **4r** exhibits hydrogen bonding between its carbonyl group and the side-chain carboxyl group of Asp1046, as well as a hydrogen bond between the benzimidazole nitrogen and Cys919. Compound **4s**, on the other hand, forms hydrogen bonds between its benzimidazole nitrogen and Cys919, while its oxadiazole oxygen interacts with Phe1047. The main scaffold of the designed compounds was investigated to determine whether it had similar protein–ligand interactions and binding poses to sorafenib through molecular modeling methods. To ensure the reliability of the molecular docking process, validation was performed by redocking sorafenib into the VEGFR2 crystal structure, achieving an RMSD of 0.9 Å, which confirms the accuracy of the docking protocol. The designed compounds with a benzimidazole scaffold were subjected to the same redocking process under identical conditions. Eighteen compounds were selected based on their docking scores and substituents (Table 4). From these, compounds **4r** and **4s** were chosen for molecular dynamic interaction display and molecular dynamic simulations based on their docking scores. According to biological activity data, the most active compounds were **4r** and **4s**. As shown in Figure 5a, compound **4s** and sorafenib exhibited similar binding poses in the active site. Figure 5b–d presents the two-dimensional protein–ligand interactions of sorafenib, **4r**, and **4s**, respectively. The similar binding poses and hydrogen bond interactions of compounds **4r** and **4s** with key VEGFR2 active site residues, such as Asp1046 and Cys919, strongly suggest that these compounds could act as competitive inhibitors, mimicking sorafenib’s mechanism of action.

**Table 4:** Molecular Docking Protein–Ligand Binding Energies and Theoretical ADME Parameters of the Designed Potential VEGFR2 Inhibitors

	docking		ADME						
comp.	glide XP score	glide EModel	mol MW	QPlogP o/w	QPlogS	QPPCaco	QPP MDCK	HOA	rule of five
**4a**	–11.471	–97.776	428.464	3.961	–6.807	209.031	149.768	1	0
**4b**	–11.726	–105.297	442.491	4.265	–7.369	208.512	149.436	1	0
**4c**	–11.686	–105.213	462.909	4.346	–7.513	151.130	259.815	1	0
**4d**	–11.709	–101.973	446.455	4.068	–6.978	151.065	190.284	1	0
**4e**	–11.791	–104.687	507.360	4.408	–7.536	151.605	280.241	1	1
**4f**	–11.774	–106.272	453.474	3.212	–7.791	43.252	27.255	1	0
**4g**	–11.266	–97.831	464.445	4.248	–7.208	155.173	273.429	1	0
**4h**	–12.371	–105.368	497.355	4.837	–8.043	181.572	679.882	1	0
**4i**	–12.273	–109.959	460.463	2.386	–6.089	16.246	9.485	2	0
**4j**	–11.412	–100.344	442.491	4.685	–7.259	495.021	381.071	1	0
**4k**	–10.656	–102.649	456.518	5.108	–7.876	687.696	542.212	1	1
**4l**	–11.846	–99.753	476.936	5.138	–7.786	497.105	943.723	1	1
**4m**	–11.669	–99.089	460.482	4.921	–7.619	498.247	691.172	1	0
**4n**	–11.738	–103.115	521.387	5.257	–8.113	498.194	1.014.717	1	2
**4o**	–11.474	–103.109	467.501	4.018	–8.213	142.906	99.265	1	0
**4p**	–11.624	–102.770	478.472	5.011	–7.531	509.907	900.661	1	1
**4r**	–12.308	–105.980	511.381	5.800	–8.699	812.483	3.430.188	1	2
**4s**	–12.398	–106.165	474.490	3.330	–6.699	72.446	44.756	1	0
sorafenib	–12.664	–128.963							

#### Molecular Dynamics Simulations

**Figure 6:**
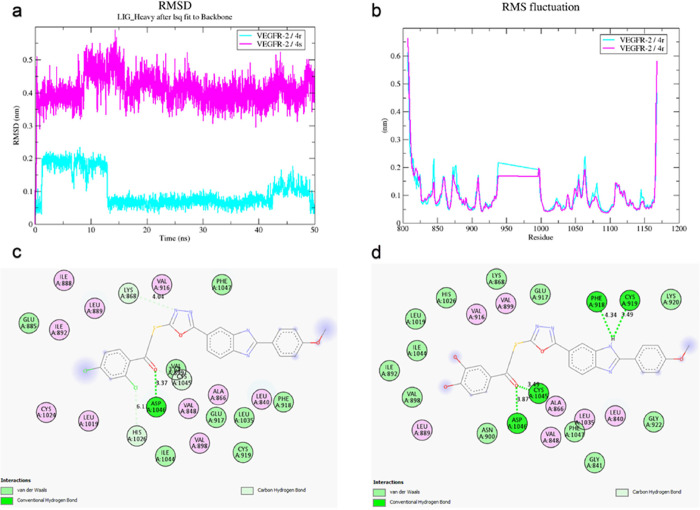
Molecular dynamics simulation of VEGFR2 with **4r** and **4s** complexes. (a) RMSD graph of VEGFR2/**4r** and **4s** complexes, (b) RMS fluctuation, (c) protein–ligand interactions after a 50 ns molecular dynamics simulation of **4r** within the VEGFR2 active site, and (d) protein–ligand interactions after a 50 ns molecular dynamics simulation of **4s** within the VEGFR2 active site.

To investigate the protein–ligand interaction stability of compounds **4r** and **4s** in a simulated physiological environment, a 50 ns molecular dynamics simulation was performed. The stability of the protein–ligand complex was evaluated by using RMSD and RMSF measurements along with trajectory analysis. RMSD provides information on the deviations/shifts of the protein and ligand. The RMSD measurement calculated how the ligand changed over time relative to that of the protein. As shown in Figure 6a, at the beginning of the simulation, compound **4s** exhibited deviations up to 0.4 nm but maintained its stability throughout the simulation. Compound **4r**, on the other hand, showed deviations between 0.1 and 0.2 nm. The stable RMSD values observed for compounds **4r** and **4s** during the 50 ns simulation further confirm their consistent interactions with the VEGFR2 binding pocket. RMSF measurements provide insights into the conformational changes and flexibility of the protein. As seen in Figure 6b, the protein–ligand complex formed by **4s** with VEGFR2 fluctuated less and was more stable compared to the **4r**-VEGFR2 complex. After 50 ns, the interactions of the **4r** and **4s** ligands within the active site are shown in Figure 5. Despite some conformational changes from the initial configuration, both **4r** and **4s** remained stable in the active site, as given in Figure 6c-**d**. The hydrogen bond interaction between the carbonyl group of **4r** and Asp1046 was maintained throughout the simulation. For compound **4s**, a hydrogen bond was formed between Asp1046 and the oxygen of the oxadiazole ring according to the docking pose. As suggested by the initial deviation in the RMSD graph, the hydrogen bond shifted from the oxadiazole oxygen atom back to the carbonyl group. Additionally, hydrogen bonds were formed between the carbonyl group and Cys1045 (SH) and Asp1046 (CO_2_H), and between the benzimidazole N and Cys919 SH. There was also an interaction (possibly π–π) between the benzimidazole group and the aromatic system of Phe918. The previously reported stable RMSD of 0.15 nm for the sorafenib-VEGFR2 complex throughout the molecular dynamics simulation not only reflects the strong binding of sorafenib but also confirms the reliability and accuracy of the MD simulation protocol.^29^ Comparing this to the RMSD values of 4r and 4s (ranging from 0.1 to 0.4 nm), it is evident that the new compounds exhibit stable binding. The results from the molecular dynamics simulations indicate that the designed compounds have the potential to form strong interactions with and inhibit VEGFR2.

#### ADME Predictions

Many drug candidates fail to gain approval due to their unsuitable physicochemical properties. Therefore, some ADME parameters of the designed compounds were calculated and evaluated against Lipinski’s Rule of Five. The data are presented in Table 4. The designed compounds were found to have suitable theoretical ADME parameters that comply with the limiting rules, and the calculated values fall within the desired ranges. The molecular weight (Mol MW) of all the compounds is below 500 Da, which aligns with Lipinski’s rule that a drug-like molecule should have a molecular weight less than 500 Da. The predicted octanol/water partition coefficient (QPlogPo/w) values for the compounds are also within the acceptable range of less than 5, indicating good membrane permeability. **4r** had a molecular weight of 511.381 Da and a QPlogPo/w value of 5.800, indicating high lipophilicity, with a predicted MDCK cell permeability value of 3,430.188, suggesting excellent membrane permeability. However, it slightly exceeded the preferred range for QPlogPo/w, which might necessitate further optimization. **4s** had a molecular weight of 474.490 Da and a QPlogPo/w value of 3.330, indicating balanced lipophilicity and good solubility. It also exhibited moderate permeability values, making it a well-rounded candidate with fewer deviations from Lipinski’s rules. These findings support the potential of the designed benzimidazole derivatives as VEGFR2 inhibitors with favorable pharmacokinetic profiles.

#### Quantum Mechanical Calculations

The main molecules in the building blocks of the compounds consist of a 1,3,4-oxadiazole ring with a 4-substitutedphenyl-benzimidazol group and a thio group attached to the R1-R2-R3-substitutedphenyl-ethanone at the fifth and second locations of this ring, respectively (Figure S1). The rotations of the thio group and the benzimidazole group with respect to the 1,3,4-oxadiazole rings are 180 deg, and the molecules are planar, according to DFT analysis.

According to the HOMO–LUMO analysis (Table 5), the order of stability in the initial group of molecules (**4a–4i**) is as follows: **4b** > **4i** > **4a** > **4d** > **4g** > **4c** > **4e** > **4h** > **4f**. Compounds **4b** and **4i** (with high Δ*E* values of 3.908 and 3.853 eV, respectively) are more stable than the other molecules of the first group. The order of stability in the second group (**4j–4s**) is as follows: **4k** > **4s** > **4j** > **4m** > **4p**> **4l** > **4n** > **4r** > **4o**. The molecules **4k** and **4s** have higher Δ*E* values (3.876 and 3.821 eV, respectively), corresponding to their greater stability.

**Table 5:** HOMO–LUMO Energies (eV) and Calculated Global Reactivity Parameters of the Best Stable States of Compounds **4a**–**4s** at the B3LYP/6-31G(d,p) Level in the Gas Phase^a^

compound	*E*_L_ (eV)	*E*_H_ (eV)	Δ*E* (eV)	IP (eV)	EA(eV)	χ (eV)	η (eV)	σ (eV)^−1^	μ (eV)	ω (eV)
**4a**	–1.844	–5.518	3.674	5.518	1.844	3.681	1.836	0.272	–3.681	3.689
**4b**	–1.562	–5.470	3.908	5.470	1.562	3.516	1.954	0.256	–3.516	3.164
**4c**	–2.080	–5.564	3.484	5.564	2.080	3.822	1.742	0.287	–3.822	4.193
**4d**	–1.882	–5.545	3.663	5.545	1.882	3.714	1.832	0.273	–3.714	3.765
**4e**	–2.086	–5.562	3.476	5.562	2.086	3.824	1.738	0.288	–3.824	4.207
**4f**	–2.669	–5.630	2.961	5.630	2.669	4.149	1.481	0.338	–4.149	5.814
**4g**	–1.994	–5.508	3.514	5.508	1.994	3.751	1.757	0.284	–3.751	4.003
**4h**	–2.280	–5.530	3.250	5.530	2.280	3.905	1.625	0.307	–3.905	4.692
**4i**	–1.607	–5.460	3.853	5.460	1.607	3.533	1.926	0.259	–3.533	3.241
**4j**	–1.836	–5.475	3.639	5.475	1.836	3.655	1.820	0.275	–3.655	3.671
**4k**	–1.554	–5.430	3.876	5.430	1.554	3.492	1.938	0.258	–3.492	3.146
**4l**	–2.072	–5.520	3.448	5.520	2.072	3.796	1.724	0.290	–3.796	4.178
**4m**	–1.874	–5.502	3.628	5.502	1.874	3.688	1.814	0.276	–3.688	3.748
**4n**	–2.078	–5.517	3.439	5.517	2.078	3.797	1.719	0.291	–3.797	4.192
**4o**	–2.660	–5.583	2.923	5.583	2.660	4.122	1.461	0.342	–4.122	5.813
**4p**	–1.985	–5.465	3.480	5.465	1.985	3.725	1.740	0.287	–3.725	3.988
**4r**	–2.272	–5.486	3.214	5.486	2.272	3.879	1.607	0.311	–3.879	4.680
**4s**	–1.598	–5.419	3.821	5.419	1.598	3.509	1.910	0.262	–3.509	3.222

a Gap Δ*E*: (*E*_LUMO_ – *E*_HOMO_), IP (−HOMO): ionization potential, EA (−LUMO): electron affinity, χ(IP + EA)/2: electronegativity, η (IP – EA)/2: chemical hardness, σ (1/2η): chemical softness, μ – (IP + EA)/2: chemical potential, ω (μ^2^/2η): electrophilic index.

The distributions of HOMOs for the all molecules exhibit a similar trend, in which they are distributed in the 1,3,4-oxadiazole ring with the 4-substitutedphenyl-benzimidazol group and the thio group, except for the R1–R2–R3-substitutedphenyl-ethanone. LUMO distributions follow the same trend for each molecule and are located in the R1–R2–R3-substitutedphenyl-ethanone group, as shown in Figure S2.

In the MEP diagram of all molecules (Figure S2), red regions indicate electron excess near the O atom of ethanone and N atoms of 1,3,4-oxadiazole and benzimidazole rings. In addition, the blue regions of all molecules, which are electron-deficient, are located around the NH of the benzimidazole rings. The red and blue regions are responsible for the phenomena of electrophilic and nucleophilic attacks, respectively; they represent potential protein–ligand interaction sites, a finding that has also been corroborated through molecular docking.

## Conclusions

This study demonstrates that the newly designed benzimidazole-oxadiazole derivatives exhibit significant potential as VEGFR2 inhibitors for advancing cancer therapy. All compounds showed moderate to strong anticancer efficacy against the human lung adenocarcinoma cell line (A549), the human breast cancer cell line (MCF-7), and the human pancreas cell line (PANC-1). The IC_50_ values for compounds **4r** and **4s** were more potent than those for the other compounds, at 5.5 μM and 6.6 μM for PANC-1, 0.3 and 1.6 μM for A549, 0.5 and 1.2 μM for MCF-7. The selectivity index of compound **4r** was 1.98 and 2.66 for PANC-1 and A549, respectively. The selectivity index of compound **4s** was 5.80 and 0.81 for PANC-1 and A549, respectively. Following that, the VEGFR2 inhibitory activities of compounds **4r** and **4s** were investigated. VEGFA immunolocalization of compounds **4r** and **4s** was evaluated relative to the control by VEGFA immunofluorescence staining. Molecular docking and molecular dynamics simulations demonstrated that compounds **4r** and **4s** exhibited stable binding to VEGFR2, replicating the interactions between sorafenib and key active site residues, including Asp1046 and Cys919.

## Materials and Methods

### Synthesis

All of the chemicals employed in the synthetic procedure were purchased from Sigma-Aldrich Chemicals (Sigma-Aldrich Corp., St. Louis, MO, USA) or Merck Chemicals (Merck KGaA, Darmstadt, Germany). Melting points of the obtained compounds were determined by an MP90 digital melting point apparatus (Mettler Toledo, OH, USA) and were uncorrected. ^1^H NMR and ^13^C NMR spectra of the synthesized compounds were performed by a Bruker 300 and 75 MHz digital FT-NMR spectrometer (Bruker Bioscience, Billerica, MA, USA) in DMSO-d_6_, respectively. Splitting patterns were designated as follows: s: singlet; d: doublet; t: triplet; and m: multiplet in the NMR spectra. Coupling constants (J) were reported as Hertz. All reactions were monitored by thin-layer chromatography (TLC) using Silica Gel 60 F254 TLC plates (Merck KGaA, Darmstadt, Germany). Mass spectra were recorded on an LCMS-IT-TOF (Shimadzu,Kyoto, Japan) instrument using electrospray ionization (ESI).

### Synthesis of Methyl-2-(4-substitutedphenyl)-1*H*-benzimidazole-6-carboxylate Derivatives (1a, 1b)

4-Substituted benzaldehyde (0.03 mol), sodium metabisulfite (5.7 g, 0.03 mol), and DMF (10 mL) were placed in a microwave synthesis reactor vial (30 mL) and kept in the microwave synthesis reactor at 240 °C under 10 bar pressure for 5 min. At the end of this period, the mixture was removed from the reactor, and methyl-3,4-diaminobenzoate (4.98 g, 0.03 mol) was added. Additionally, the reaction was subjected to microwave irradiation for another 5 min under the same reaction conditions. At the end of the reaction period, the product was poured into ice water and precipitated, filtered, washed with plenty of water, and crystallized from ethanol.

### Synthesis of 2-(4-Substitutedphenyl)-1*H*-benzimidazole-6-carbohydrazide Derivatives (2a, 2b)

Methyl-2-(4-substitutedphenyl)-1*H*-benzimidazole-6-carboxylate (0.02 mol), ethanol (15 mL), and hydrazine hydrate (5 mL) were added to a microwave synthesis reactor vial (30 mL) and heated at 240 °C in the microwave synthesis reactor. It was kept under 10 bar of pressure at 240 °C for 10 min. At the end of the reaction period, the product was poured into ice water and precipitated, filtered, washed with plenty of water, and crystallized from ethanol.

### Synthesis of 5-(2-(4-Substitutedphenyl)-1*H*-benzimidazol-6-yl)-1,3,4-oxadiazole-2-thiol Derivatives (3a, 3b)

Sodium hydroxide (0.96 g, 0.024 mol) was dissolved in ethanol (10 mL), and a 2-(4-substituted phenyl)-1*H*-benzimidazole-6-carbohydrazide (0.02 mol) derivative was added. Carbon disulfide (1.45 mL, 0.024 mol) in ethanol (10 mL) was placed in a dropping funnel and added dropwise to the reaction mixture. At the end of the reaction, HCl was added to the product until pH = 5, and the product was precipitated by pouring it into ice water and crystallized from ethanol by washing with plenty of water.

### Synthesis of Target Compounds (4a–4s)

The compound 5-(2-(4-substitutedphenyl)-1*H*-benzimidazol-6-yl)-1,3,4-oxadiazole-2-thiol (**3**) (0.001 mol) was dissolved in acetone, and the appropriate 2-bromoacetophenone derivative (0.001 mol) were added. The reaction mixture was kept at 40 °C under reflux for 12 h, and acetone (20 mL) was evaporated at the end of the reaction. The remaining substance was filtered with water, dried, and crystallized from ethanol.

#### 2-((5-(2-(4-Hydroxyphenyl)-1*H*-benzimidazol-6-yl)-1,3,4-oxadiazol-2-yl)thio)-1-phenyl-ethanone (4a)

Yield: 66%, M.P. = 302.5 °C. ^1^H NMR (300 MHz, DMSO-d_6_): δ: 5.20 (2H, s, CH_2_), 6.93 (2H, d, *J* = 8.64 Hz, Aromatic CH), 7.57–7.62 (2H, m, Aromatic CH), 7.70–7.78 (3H, m, Aromatic CH), 8.02 (2H, d, *J* = 8.64 Hz, Aromatic CH) 8.08–8.10 (3H, m, Aromatic CH), 10.07 (1H, s, OH). ^13^C NMR (75 MHz, DMSO-d_6_): δ = 40.99, 116.01, 116.28, 116.72, 117.40, 120.75, 120.81, 128.97, 129.03, 129.39, 132.41, 134.48, 135.52, 154.68, 160.17, 162.95, 166.56, 178.56, 193.26. HRMS (*m*/*z*): [M + H]^+^ calcd for C_23_H_16_N_4_O_3_S: 429.1010; found: 429.1016.

#### 2-((5-(2-(4-Hydroxyphenyl)-1*H*-benzimidazol-6-yl)-1,3,4-oxadiazol-2-yl)thio)-1-(4-methylphenyl)-ethanone (4b)

Yield: 64%, M.P.= 271.1 °C. ^1^H NMR (300 MHz, DMSO-d_6_): δ: 2.41 (3H, s, CH_3_), 5.16 (2H, s, CH_2_), 6.93 (2H, d, *J* = 8.73 Hz, Aromatic CH), 7.60 (2H, d, *J* = 7.80 Hz, Aromatic CH), 7.69–7.73 (2H, m, Aromatic CH), 7.40 (2H, d, *J* = 8.01 Hz, Aromatic CH), 7.68 (1H, d, *J* = 8.40 Hz, Aromatic CH), 7.76 (1H, dd, *J*_1_ = 8.40 Hz, *J*_*2*_ = 1.56 Hz, Aromatic CH), 7.98 (2H, d, *J* = 8.22 Hz, Aromatic CH), 8.03 (2H, d, *J* = 8.70 Hz, Aromatic CH), 8.07 (1H, s, Aromatic CH), 10.08 (1H, s, OH). ^13^C NMR (75 MHz, DMSO-d_6_): δ= 21.71, 40.90, 116.29, 116.78, 120.69, 120.79, 124.99, 128.76, 129.09, 129.93, 133.03, 137.15, 142.10, 145.07, 152.95, 154.65, 160.21, 163.02, 166.51, 192.76. HRMS (*m*/*z*): [M + H]^+^ calcd for C_24_H_18_N_4_O_3_S: 443.1177; found: 443.1172.

#### 2-((5-(2-(4-Hydroxyphenyl)-1*H*-benzimidazol-6-yl)-1,3,4-oxadiazol-2-yl)thio)-1-(4-chlorophenyl)-ethanone (4c)

Yield: 72%, M.P.= 277.0 °C. ^1^H NMR (300 MHz, DMSO-d_6_): δ: 5.17 (2H, s, CH_2_), 6.93 (2H, d, *J* = 8.01 Hz, Aromatic CH), 7.65–7.66 (3H, m, Aromatic CH), 7.69–7.76 (1H, m, Aromatic CH), 8.03 (2H, d, *J* = 8.64 Hz, Aromatic CH), 8.09–8.14 (3H, m, Aromatic CH), 10.05 (1H, s, OH), 13.02 (1H, s, NH). ^13^C NMR (75 MHz, DMSO-d_6_): δ = 42.05, 109.71, 112.47, 116.27, 116.71, 117.18, 119.56, 120.53, 120.90, 129.07, 129.52, 130.89, 134.23, 139.42, 155.02, 160.11, 162.78, 166.57, 192.43. HRMS (*m*/*z*): [M + H]^+^ calcd for C_23_H_15_N_4_O_3_SCl: 463.0638; found: 463.0626.

#### 2-((5-(2-(4-Hydroxyphenyl)-1*H*-benzimidazol-6-yl)-1,3,4-oxadiazol-2-yl)thio)-1-(4-florophenyl)-ethanone (4d)

Yield: 74%, M.P. = 267.4 °C. ^1^H NMR (300 MHz, DMSO-d_6_): δ: 4.99 (2H, s, CH_2_), 6.76 (2H, d, *J* = 8.55 Hz, Aromatic CH), 7.21–7.27 (3H, m, Aromatic CH), 7.51–7.56 (3H, m, Aromatic CH), 7.85–7.88 (3H, m, Aromatic CH), 9.94 (1H, s, OH), 13.00 (1H, s, NH). ^13^C NMR (75 MHz, DMSO-d_6_): δ = 40.69, 116.09, 116.15, 116.46, 116.72, 117.10, 120.47, 120.61, 128.87, 129.46, 131.85, 131.98, 135.29, 135.71, 159.72, 160.02, 162.70, 166.41, 191.77. HRMS (*m*/*z*): [M + H]^+^ calcd for C_23_H_15_N_4_O_3_FS: 447.0932; found: 447.0922.

#### 2-((5-(2-(4-Hydroxyphenyl)-1*H*-benzimidazol-6-yl)-1,3,4-oxadiazol-2-yl)thio)-1-(4-bromophenyl)-ethanone (4e)

Yield: 73%, M.P. = 272.8 °C. ^1^H NMR (300 MHz, DMSO-d_6_): δ: 5.17 (2H, s, CH_2_), 6.93 (2H, d, *J* = 8.43 Hz, Aromatic CH), 7.62 (1H, d, *J* = 8.37 Hz, Aromatic CH), 7.74 (1H, s, Aromatic CH), 7.82 (2H, d, *J* = 8.58 Hz, Aromatic CH), 8.00–8.05 (5H, m, Aromatic CH), 10.08 (1H, s, OH), 13.04 (1H, s, NH). ^13^C NMR (75 MHz, DMSO-d_6_): δ= 40.82, 109.70, 112.46, 116.27, 117.18, 119.56, 120.90, 128.66, 128.99, 130.96, 132.47, 134.55, 135.59, 144.42, 155.01, 160.22, 162.87, 166.62, 192.67. HRMS (*m*/*z*): [M + H]^+^ calcd for C_23_H_15_N_4_O_3_SBr: 507.0130; found: 507.0121.

#### 2-((5-(2-(4-Hydroxyphenyl)-1*H*-benzimidazol-6-yl)-1,3,4-oxadiazol-2-yl)thio)-1-(4-cyanophenyl)-ethanone (4f)

Yield: 71%, M.P. = 127.3 °C. ^1^H NMR (300 MHz, DMSO-d_6_): δ: 5.17 (2H, s, CH_2_), 6.94 (2H, d, *J* = 7.08 Hz, Aromatic CH), 7.40 (2H, d, *J* = 6.93 Hz, Aromatic CH), 7.71–7.79 (2H, m, Aromatic CH), 7.98–8.07 (5H, m, Aromatic CH), 10.10 (1H, s, OH). ^13^C NMR (75 MHz, DMSO-d_6_): δ= 43.11, 114.07, 114.23, 115.23, 116.99, 117.90, 118.49, 120.97, 122.18, 125.73, 127.61, 128.08, 128.17, 130.82, 131.58, 132.02, 132.30, 135.27, 151.23, 151.81, 153.66, 167.06, 194.09. HRMS (*m*/*z*): [M + H]^+^ calcd for C_24_H_15_N_5_O_3_S: 454.0986; found: 454.0968.

#### 2-((5-(2-(4-Hydroxyphenyl)-1*H*-benzimidazol-6-yl)-1,3,4-oxadiazol-2-yl)thio)-1-(2,4-diflorophenyl)-ethanone (4g)

Yield: 78%, M.P. = 284.7 °C. ^1^H NMR (300 MHz, DMSO-d_6_): δ: 5.05 (2H, s, CH_2_), 6.95 (2H, d, *J* = 8.28 Hz, Aromatic CH), 7.28–7.33 (1H, m, Aromatic CH), 7.48–7.55 (1H, m, Aromatic CH), 7.63–7.74 (2H, m, Aromatic CH), 8.01–8.13 (4H, m, Aromatic CH), 10.17 (1H, s, OH), 13.33 (1H, s, NH). ^13^C NMR (75 MHz, DMSO-d_6_): δ= 44.00, 105.54, 106.25, 109.78, 112.52, 113.26, 116.26, 116.54, 117.08, 119.50, 120.80, 129.10, 133.29, 135.65, 144.40, 147.09, 160.17, 162.84, 166.63, 167.62, 189.81. HRMS (*m*/*z*): [M + H]^+^ calcd for C_23_H_14_N_4_O_3_F_2_S: 465.0833; found: 465.0827.

#### 2-((5-(2-(4-Hydroxyphenyl)-1*H*-benzimidazol-6-yl)-1,3,4-oxadiazol-2-yl)thio)-1-(2,4-dichlorophenyl)-ethanone (4h)

Yield: 69%, M.P. = 163.5 °C. ^1^H NMR (300 MHz, DMSO-d_6_): δ: 5.20 (2H, s, CH_2_), 7.10 (1H, d, *J* = 8.88 Hz, Aromatic CH), 7.56–7.77 (5H, m, Aromatic CH), 8.01–8.15 (5H, m, Aromatic CH). ^13^C NMR (75 MHz, DMSO-d_6_): δ = 40.93,114.05, 114.24, 117.04, 118.07, 118.36, 120.92, 122.03, 125.88, 127.09, 129.10, 129.74, 129.93, 133.02, 145.08, 151.78, 153.66, 163.09, 166.54, 167.19, 192.74. HRMS (*m*/*z*): [M + H]^+^ calcd for C_23_H_14_N_4_O_3_SCl_2_: 497.0255; found: 497.0236.

#### 2-((5-(2-(4-Hydroxyphenyl)-1*H*-benzimidazol-6-yl)-1,3,4-oxadiazol-2-yl)thio)-1-(3,4-dihydroxyphenyl)-ethanone (4i)

Yield: 72%, M.P. = 152.1 °C. ^1^H NMR (300 MHz, DMSO-d_6_): δ: 5.17 (2H, s, CH_2_), 7.14 (1H, d, *J* = 7.89 Hz, Aromatic CH), 7.39–7.42 (3H, m, Aromatic CH), 7.91–7.98 (4H, m, Aromatic CH), 8.00 (1H, s, Aromatic CH), 8.14–8.16 (1H, m, Aromatic CH). ^13^C NMR (75 MHz, DMSO-d_6_): δ = 48.11, 114.11, 114.29, 115.70, 116.13, 116.34, 116.55, 117.24, 118.84, 121.14, 122.52, 125.47, 129.29, 131.95, 132.04, 151.90, 153.67, 163.05, 166.49, 166.97, 191.92. HRMS (*m*/*z*): [M + H]^+^ calcd for C_23_H_16_N_4_O_5_S: 461.0923; found: 461.0914.

#### 2-((5-(2-(4-Methoxyphenyl)-1*H*-benzimidazol-6-yl)-1,3,4-oxadiazol-2-yl)thio)-1-phenyl-ethanone (4j)

Yield: 75%, M.P. = 207.7 °C. ^1^H NMR (300 MHz, DMSO-d_6_): δ: 3.85 (3H, s, OCH_3_), 5.21 (2H, s, CH_2_), 7.13 (2H, d, *J* = 7.20 Hz, Aromatic CH), 7.58–7.63 (2H, m, Aromatic CH), 7.70–7.82 (3H, m, Aromatic CH), 8.08–8.16 (5H, m, Aromatic CH). ^13^C NMR (75 MHz, DMSO-d_6_): δ = 40.99, 55.88, 109.83, 112.62, 114.97, 116.88, 117.34, 119.75, 120.61, 121.10, 128.97, 129.40, 134.49, 135.52, 147.02, 154.57, 151.52, 162.94, 166.49, 193.27. HRMS (*m*/*z*): [M + H]^+^ calcd for C_24_H_18_N_4_O_3_S: 443.1176; found: 443.1172.

#### 2-((5-(2-(4-Methoxyphenyl)-1*H*-benzimidazol-6-yl)-1,3,4-oxadiazol-2-yl)thio)-1-(4-methylphenyl)-ethanone (4k)

Yield: 77%, M.P. = 250.8 °C. ^1^H NMR (300 MHz, DMSO-d_6_): δ: 3.85 (3H, s, OCH_3_), 5.15 (2H, s, CH_2_), 7.13 (2H, d, *J* = 8.91 Hz, Aromatic CH), 7.39 (2H, d, *J* = 8.04 Hz, Aromatic CH), 7.71–7.79 (2H, m, Aromatic CH), 7.97 (2H, d, J = 8.22 Hz, Aromatic CH), 8.13 (2H, d, *J* = 7.08 Hz, Aromatic CH), 13.13 (1H, s, NH). ^13^C NMR (75 MHz, DMSO-d_6_): δ= 21.72, 42.14, 55.87, 114.96, 116.88, 118.78, 119.73, 120.87, 122.38, 125.49, 126.26, 128.87, 129.09, 129.92, 133.02, 145.07, 154.23, 161.56, 163.03, 166.49, 192.74. HRMS (*m*/*z*): [M + H]^+^ calcd for C_25_H_20_N_4_O_3_S: 457.1319; found: 457.1329.

#### 2-((5-(2-(4-Methoxyphenyl)-1*H*-benzimidazol-6-yl)-1,3,4-oxadiazol-2-yl)thio)-1-(4-chlorophenyl)-ethanone (4l)

Yield: 66%, M.P. = 244.9 °C. ^1^H NMR (300 MHz, DMSO-d_6_): δ: 3.85 (3H, s, OCH_3_), 5.18 (2H, s, CH_2_), 7.12 (2H, d, *J* = 8.70 Hz, Aromatic CH), 7.67 (2H, d, *J* = 8.61 Hz, Aromatic CH), 7.76–7.80 (2H, m, Aromatic CH), 8.10 (2H, d, *J* = 8.37 Hz, Aromatic CH), 8.16–8.19 (3H, m, Aromatic CH), 13.35 (1H, s, NH). ^13^C NMR (75 MHz, DMSO-d_6_): δ= 40.88, 55.87, 109.89, 112.63, 114.94, 116.79, 117.32, 119.69, 122.42, 128.88, 129.52, 130.90, 134.23, 135.65, 139.41, 144.35, 154.01, 161.50, 166.55, 192.45. HRMS (*m*/*z*): [M + H]^+^ calcd for C_24_H_17_N_4_O_3_SCl: 477.0794; found: 477.0783.

#### 2-((5-(2-(4-Methoxyphenyl)-1*H*-benzimidazol-6-yl)-1,3,4-oxadiazol-2-yl)thio)-1-(4-florophenyl)-ethanone (4m)

Yield: 70%, M.P. = 165.8 °C. ^1^H NMR (300 MHz, DMSO-d_6_): δ: 3:85 (3H, s, OCH_3_), 5.06 (2H, s, CH_2_), 7.13–7.16 (1H, m, Aromatic CH), 7.36–7.47 (5H, m, Aromatic CH), 8.10–8.17 (5H, m, Aromatic CH). ^13^C NMR (75 MHz, DMSO-d_6_): δ = 42.03, 56.00, 114.98, 116.26, 116.35, 116.55, 116.86, 122.34, 128.89, 131.97, 132.09, 132.39, 138.96, 143.52, 152.97, 154.53, 156.48, 164.17, 167.51, 191.82. HRMS (*m*/*z*): [M + H]^+^ calcd for C_24_H_17_N_4_O_3_FS: 461.1094; found: 461.1078.

#### 2-((5-(2-(4-Methoxyphenyl)-1*H*-benzimidazol-6-yl)-1,3,4-oxadiazol-2-yl)thio)-1-(4-bromophenyl)-ethanone (4n)

Yield: 73%, M.P. = 157.3 °C. ^1^H NMR (300 MHz, DMSO-d_6_): δ: 3.36 (3H, s, OCH_3_), 5.06 (2H, s, CH_2_), 7.11 (2H, d, *J* = 8.79 Hz, Aromatic CH), 7.62–7.78 (5H, m, Aromatic CH), 8.02–8.18 (5H, m, Aromatic CH). ^13^C NMR (75 MHz, DMSO-d_6_): δ= 40.90, 55.87, 109.95, 112.66, 114.92, 116.77, 117.34, 120.56, 121.06, 122.40, 128.99, 129.52, 130.90, 134.21, 139.42, 154.59, 161.50, 162.81, 166.58, 192.84. HRMS (*m*/*z*): [M + H]^+^ calcd for C_24_H_17_N_4_O_3_SBr: 521.0274; found: 521.0277.

#### 2-((5-(2-(4-Methoxyphenyl)-1*H*-benzimidazol-6-yl)-1,3,4-oxadiazol-2-yl)thio)-1-(4-cyanophenyl)-ethanone (4o)

Yield: 75%, M.P. = 129.1 °C. ^1^H NMR (300 MHz, DMSO-d_6_): δ: 3.36 (3H, s, OCH_3_), 5.16 (2H, s, CH_2_), 6.94 (2H, d, *J* = 8.55 Hz, Aromatic CH), 7.40 (2H, d, *J* = 7.98 Hz, Aromatic CH), 7.67–7.78 (3H, m, Aromatic CH), 7.97–8.05 (4H, m, Aromatic CH). ^13^C NMR (75 MHz, DMSO-d_6_): δ= 40.91, 57.40, 116.28, 116.54, 116.73, 117.06, 117.24, 117.51, 120.79, 121.10, 123.01, 129.04, 129.10, 129.50, 129.93, 133.02, 145.08, 160.17, 163.00, 166.53, 192.75. HRMS (*m*/*z*): [M + H]^+^ calcd for C_25_H_17_N_5_O_3_S: 468.1145; found: 468.1125.

#### 2-((5-(2-(4-Methoxyphenyl)-1*H*-benzimidazol-6-yl)-1,3,4-oxadiazol-2-yl)thio)-1-(2,4-diflorophenyl)-ethanone (4p)

Yield: 70%, M.P. = 206.3 °C. ^1^H NMR (300 MHz, DMSO-d_6_): δ: 3.85 (3H, s, OCH_3_), 5.05 (2H, s, CH_2_), 7.14 (2H, d, *J* = 8.82 Hz, Aromatic CH), 7.28–7.33 (2H, m, Aromatic CH), 7.49–7.56 (2H, m, Aromatic CH), 7.71–7.81 (2H, m, Aromatic CH), 8.16 (2H, d, *J* = 8.70 Hz, Aromatic CH). ^13^C NMR (75 MHz, DMSO-d_6_): δ = 40.79, 60.70, 105.05, 105.46, 107.44, 109.73, 112.90, 113.30, 114.99, 116.34, 119.90, 122.09, 124.37, 126.35, 128.96, 132.37, 136.32, 136.84, 140.99, 156.99, 162.50, 191.29. HRMS (*m*/*z*): [M + H]^+^ calcd for C_24_H_16_N_4_O_3_F_2_S: 479.0975; found: 479.0984.

#### 2-((5-(2-(4-Methoxyphenyl)-1*H*-benzimidazol-6-yl)-1,3,4-oxadiazol-2-yl)thio)-1-(2,4-dichlorophenyl)-ethanone (4r)

Yield: 68%, M.P. = 108.7 °C. ^1^H NMR (300 MHz, DMSO-d_6_): δ: 4.26 (3H, s, OCH_3_), 5.43 (2H, s, CH_2_), 7.54 (2H, br.s., Aromatic CH), 7.91–8.18 (4H, m, Aromatic CH), 8.34 (1H, s, Aromatic CH), 8.55 (3H, br.s, Aromatic CH). ^13^C NMR (75 MHz, DMSO-d_6_): δ= 43.18, 56.30, 115.39, 117.22, 119.59, 121.30, 122.75, 125.10, 126.03, 128.57, 129.31, 131.24, 132.39, 135.49, 137.98, 139.44, 154.19, 155.75, 161.99, 166.97, 194.71. HRMS (*m*/*z*): [M + H]^+^ calcd for C_24_H_16_N_4_O_3_SCl_2_: 511.0396; found: 511.0393.

#### 2-((5-(2-(4-Methoxyphenyl)-1*H*-benzimidazol-6-yl)-1,3,4-oxadiazol-2-yl)thio)-1-(3,4-dihydroxyphenyl)-ethanone (4s)

Yield: 72%, M.P. = 254.1 °C. ^1^H NMR (300 MHz, DMSO-d_6_): δ: 3.85 (3H, s, OCH_3_), 5.04 (2H, s, CH_2_), 6.82 (1H, d, *J* = 8.28 Hz, Aromatic CH), 7.12 (2H, d, *J* = 8.82 Hz, Aromatic CH), 7.41 (1H, s, Aromatic CH), 7.48 (1H, dd, *J*_*1*_ = 8.31 Hz, *J*_*2*_ = 2.01 Hz, Aromatic CH), 7.69–7.79 (2H, m, Aromatic CH), 8.09 (1H, s, Aromatic CH), 8.17 (2H, *J* = 8.85 Hz, Aromatic CH). ^13^C NMR (75 MHz, DMSO-d_6_): δ = 40.77, 55.84, 114.94, 115.01, 115.44, 115.63, 116.48, 116.87, 120.37, 120.80, 122.44, 122.92, 126.08, 128.70, 128.93, 146.33, 153.94, 154.31, 161.53, 163.30, 166.42, 190.85. HRMS (*m*/*z*): [M + H]^+^ calcd for C_24_H_18_N_4_O_5_S: 475.1081; found: 475.1071.

### Anticancer Activity

#### MTT Method

Many analyses are performed to determine cell proliferation. MTT analysis is one of the viability determination tests. According to the working mechanism of the method, the dye containing the tetrazolium ring reacts with the dehydrogenase enzymes in the cells, only to be reduced and broken down by the metabolic cells, turning into water-insoluble formazan crystals. Color changes associated with mitochondrial activity and resulting from the addition of a solvent as a result of the reaction are measured in the microplate reader as optical density data values.^30^ Cells were planted in flat-bottomed, 96-well, sterile, lidded, disposable microplates with 1 × 10^4^ cells per well in 200 μL of appropriate medium and were allowed to adhere to the plate bottom by incubating for 24 h. At the end of 24 h, 100 μL of medium was withdrawn, and test substances at different concentrations, prepared in 100 μL, were added. Following the substance application, after a 24 h incubation period, 20 μL of stock MTT solution (MTT solution dissolved in 5 mg/mL PBS and filtered before application) was added to each well and protected from light, and the incubation continued for 3 h. Then, the media of the cells were completely withdrawn, and 100 μL of DMSO was added to dissolve the formazan crystals. After shaking in the dark for 15 min, the optical densities (absorbance) of the color changes at 540–570 and 650 nm reference wavelengths were read in the microplate reader. The data were calculated and created in Microsoft Office Excel. For this purpose, first, the blind well value and the control groups of colored substances were removed from the optical readings. The averages of optical densities and % viability values were made from Excel formula calculations, and the results were discussed. The viability of the control group was assumed to be 100%, and all other viability values were calculated based on this (series were run in *n* = 5 replicates).

#### VEGFA Immunofluorescent Staining

For VEGFA immunofluorescent staining, cells were grown on sterile coverslips in 6-well culture dishes. The cells were purified from the medium and washed with phosphate-buffered saline (PBS) (Sigma-Aldrich, Germany). The washed cells were fixed in ph 7.4% paraformaldehyde (Merk, Germany) for 20 min at room temperature. After fixation, the cells were washed two times with PBS for 5 min. For permeability, the cells were incubated in 0.1% Triton X-100 (Sigma-Aldrich, Germany) solution for 10 min. PBS was then washed for 5 min. After washing, Ultra V Block (Thermo Scientific, PBQ180830, USA) was dropped and waited at room temperature for 30 min to prevent nonspecific binding. Then, the primary rabbit polyclonal antibody anti-VEGFA (STJ96235, St John’s Laboratory Ltd., UK) was dripped onto the cells. The cells were incubated overnight at +4 °C in a dark and humid environment. At the end of the period, the cells were washed twice with PBS for 5 min. The secondary antibody, Goat Anti-Rabbit IgG H&L (ab150077, Alexa Fluor 488, Abcam, USA), was used for primary antibody detection. Secondary antibodies were diluted to 1:200 with antibody diluent reagent (Invitrogen, USA) and applied to the cells for 1 h in a humidity chamber. Then, 0.1 μg/mL was dyed with 4 ′6-diamidino-2-phenylindole dihydrochloride (DAPI, Sigma-Aldrich, Germany), and after washing with PBS, the coverslips in the culture dishes were removed and inverted onto the slide. Fluorescent microscopy (Olympus BX51, Japan) was employed by using suitable filters for fluorescence examination and then by making a registration.

#### Assessment of VEGFR2 Inhibition

The VEGFR2 Kinase Assay Kit was used for VEGFR2 inhibition. The experiment was performed in vitro according to the kit procedure.^31^

### Molecular Modeling

#### Molecular Docking

The molecular modeling study was carried out with the Schrodinger 2021-2 program. In the first stage, it was imported into the ‘Protein Preparation Wizard’ module with the code VGFR-2 PDB ID: 4ASD.^32^ The protein structure was prepared by preprocessing, removing water molecules, optimizing it, and minimizing it with OPLS3e field strengths. Ligand structures were drawn with ChemDraw 17.0 and minimized according to pH: 7 ± 2 by using the “LigPrep” module, and OPLS4 field strengths were preferred. Using the ‘Receptor Grid Generation’, a grid file of the active region of 20 × 20 × 20 Å 3 size was created in *x*: *y*: and *z*: coordinates, based on sorafenib in the 4ASD structure. In the last stage, molecular modeling was carried out with the extra precision (XP) mode of the ‘Glide’ module.^33^

#### Molecular Dynamics

Molecular dynamics simulation was performed with Gromacs version 2020.4.^34^ The topology of the ligand structures was generated by the CHARMM General Force Field (CGenFF) server, and the topology file of the VEGFR2 structure was created with the TIP3 water model of the force field forces Charmm36-Jul2020.^35^ Molecular dynamics simulations were performed under periodic boundary condition. The created protein and ligand topology files were combined and solvated, and appropriate Na+ and Cl– ions were added. The energy of the resulting VEGFR2, ligand ion, and solvent system was minimized. The system was developed with *NVT* (amount of matter, *N*; volume, *V*; and temperature, *T*) and *NPT* (amount of matter, *N*; pressure, *P*; and temperature, *T*) steps at 1 atm pressure and 300 K temperature for V-rescale and Berendsen, respectively, balanced by methods. A standard molecular dynamics simulation with a duration of 50 ns was performed. Root mean square deviation (RMSD) and root mean square fluctuation (RMSF) measurements were made with trajectory analysis gmx scripts. The results were monitored with the Visual Molecular Dynamics (VMD) and Discovery Studio Visualizer (DSV) programs.

#### ADME

Theoretical ADME calculations of the compounds were calculated with the Schrödinger ‘QikProp’ module.^36^

#### Quantum Mechanical Calculations

It is crucial to identify the precise molecular structure with the lowest energy within the structure–activity relationship. Accordingly, the DFT method was employed for the geometric optimization stage of all structures. DFT calculations were performed using the Gaussian 09 program,^37^ with the B3LYP^38,39^ exchange–correlation functional and the 6-31G(d,p) basis set. The GaussView 5.0 program^40^ was used to generate the input geometries and visualize the results. The optimized geometries of all structures were confirmed to correspond to true minima, as no imaginary frequencies were observed in the vibration frequency survey. In order to investigate the electronic properties of the current molecules, a molecular electrostatic potential (MEP) analysis was conducted together with a HOMO–LUMO analysis. Both of these were performed at the B3LYP/6-31G(d,p) level.

## Supporting Information Available

^1^H NMR, ^13^C NMR, and HRMS spectra of **4a–4s** compounds (PDF)
