# Supplementary material for: Novel Benzimidazole–Oxadiazole Derivatives as Anticancer Agents with VEGFR2 Inhibitory Activity: Design, Synthesis, In Vitro Anticancer Evaluation, and In Silico Studies
Source: ACS Omega. 2025 Feb 14;10(7):6801–13. doi: 10.1021/acsomega.4c08885 (PMC11866186; doi:10.1021/acsomega.4c08885)
Supplement: Supplementary file 1 — ao4c08885_si_001.pdf [file ao4c08885_si_001.pdf]

Novel benzimidazole-oxadiazole derivatives as anticancer agents with VEGFR2 inhibitory activity: Design, synthesis, *in vitro* anti-cancer evaluation, and *in silico* studies

Ulviye Acar Çevik[1], Ismail Celik[2], Şennur Görgülü[3], Zeynep Deniz Şahin İnan[4], Hayrani Eren Bostancı[5*], Arzu Karayel[6], Yusuf Özkay[1], Zafer Asım Kaplacıklı[1]

[1] Department of Pharmaceutical Chemistry, Faculty of Pharmacy, Anadolu University, Eskişehir 26470, Turkey.

[2] Department of Pharmaceutical Chemistry, Faculty of Pharmacy, Erciyes University, Kayseri 38039, Turkey.

[3] Medicinal Plant, Drug and Scientific Research and Application Center (AUBIBAM), 26470 Eskişehir, Turkey

[4] Department of Histology and Embryology, Sivas Cumhuriyet University, Sivas, Turkey

[5] Department of Biochemistry, Faculty of Pharmacy, Cumhuriyet University, Sivas, Turkey

[6] Department of Physics, Faculty of Arts and Science, Hitit University, 19030 Çorum, Turkey.

*Correspondence:

Corresponding Author. E-mail: erenbostanci@cumhuriyet.edu.tr

Address: Sivas Cumhuriyet University, Faculty of Pharmacy, Department of Biochemistry Sivas, Turkiye.

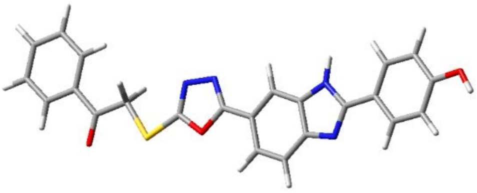
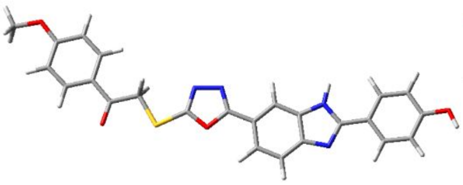
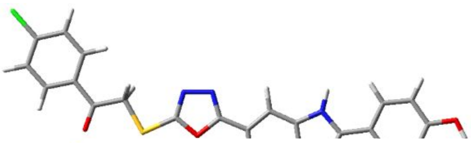
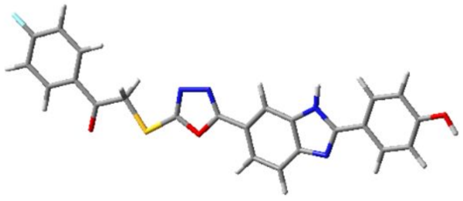

4b

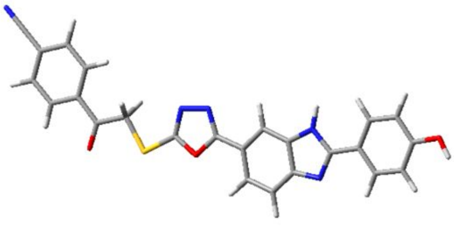

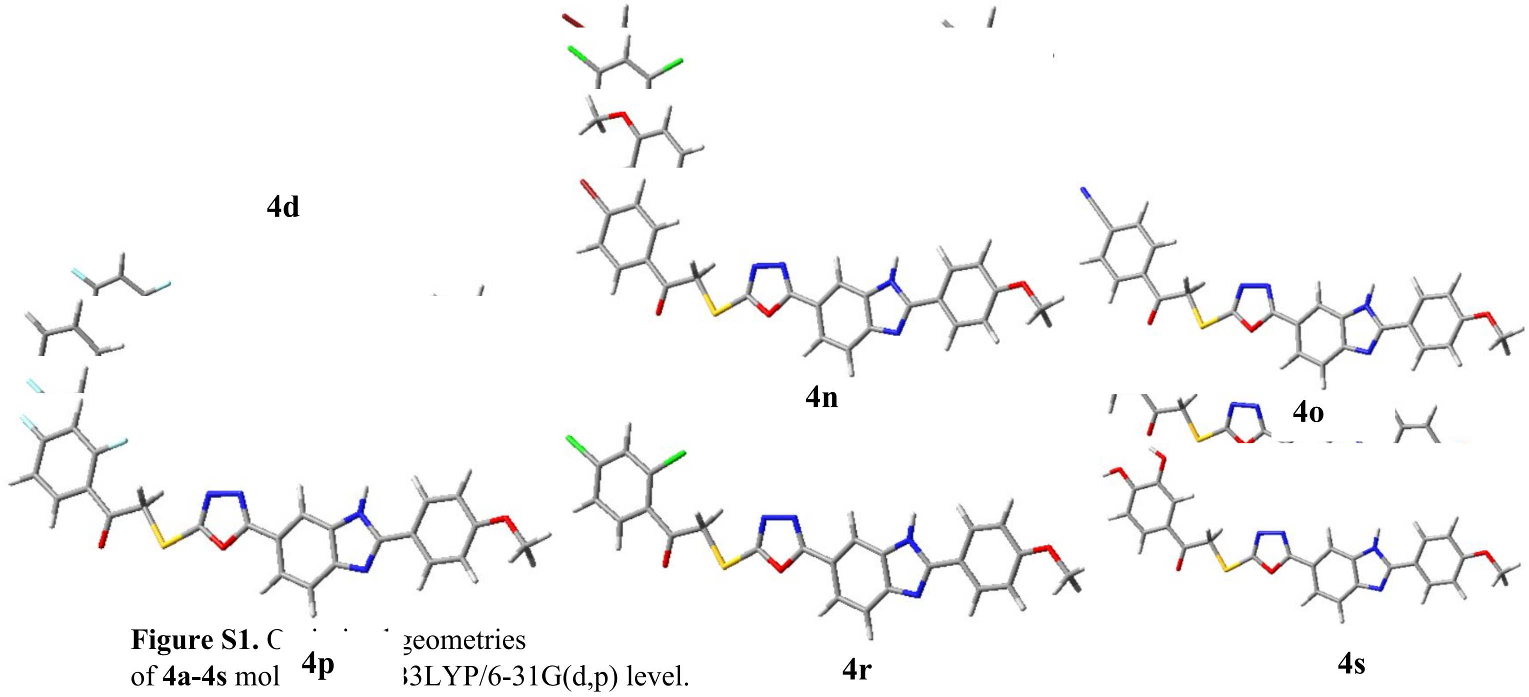

Figure S1. C[illegible] geometries of **4a-4s** mol[illegible]3LYP/6-31G(d,p) level.

MEP **HOMO** **LUMO**

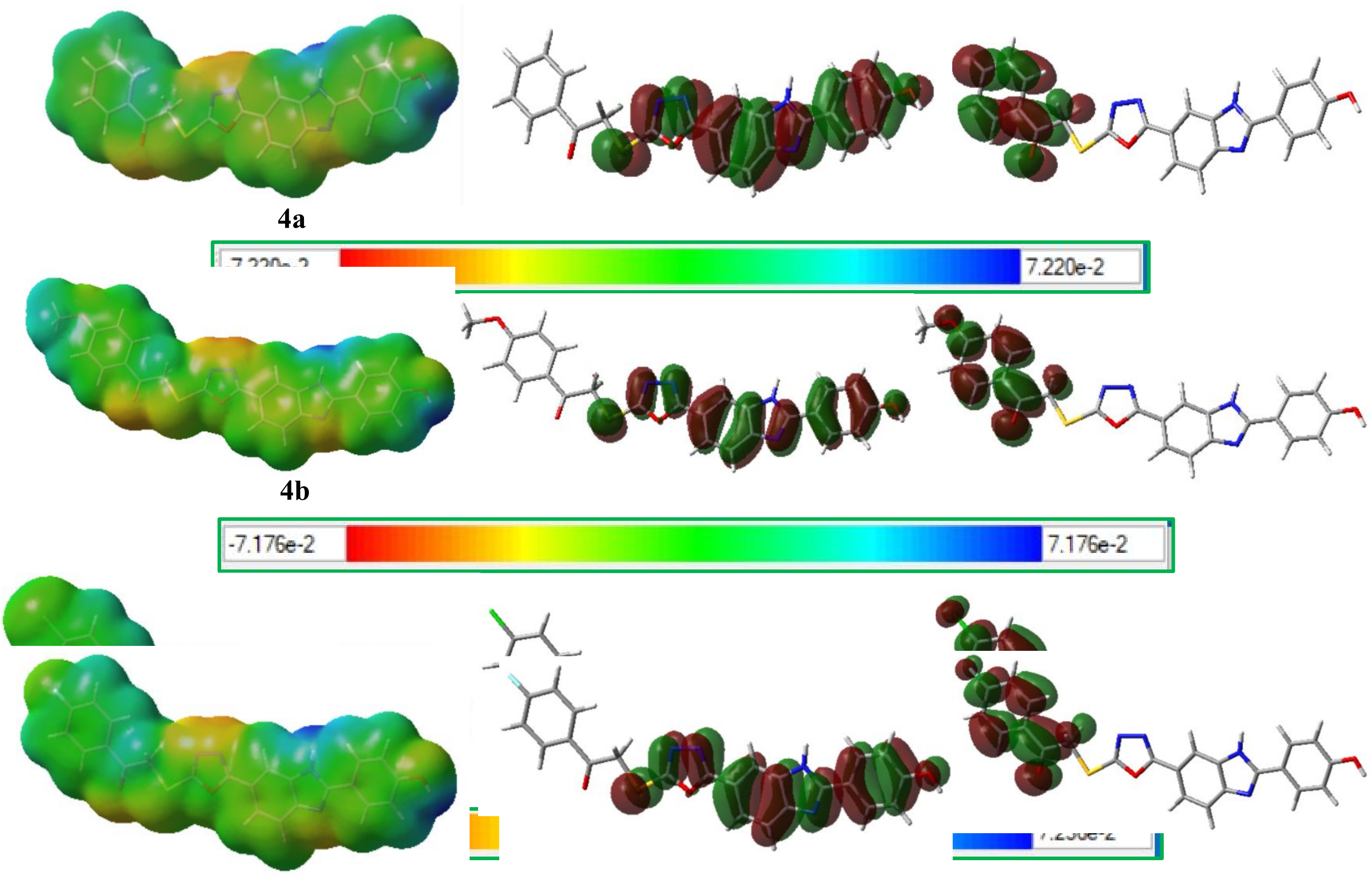

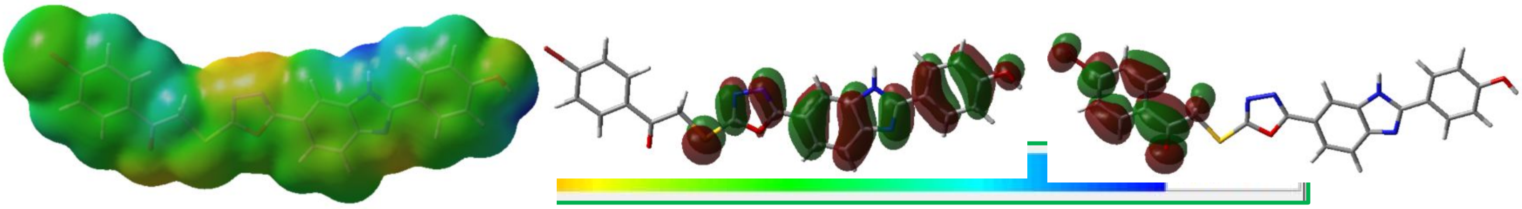

4e

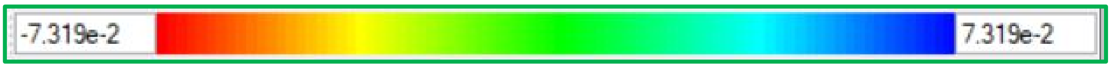

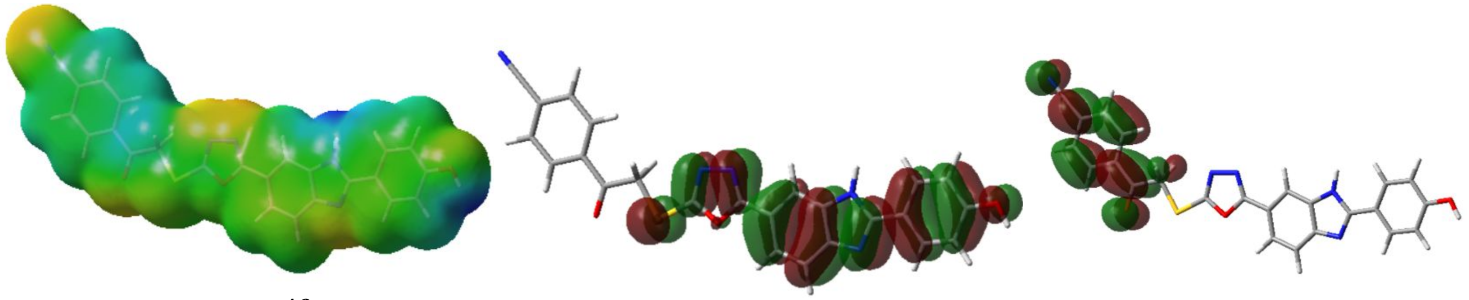

4f

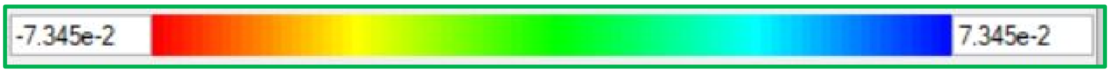

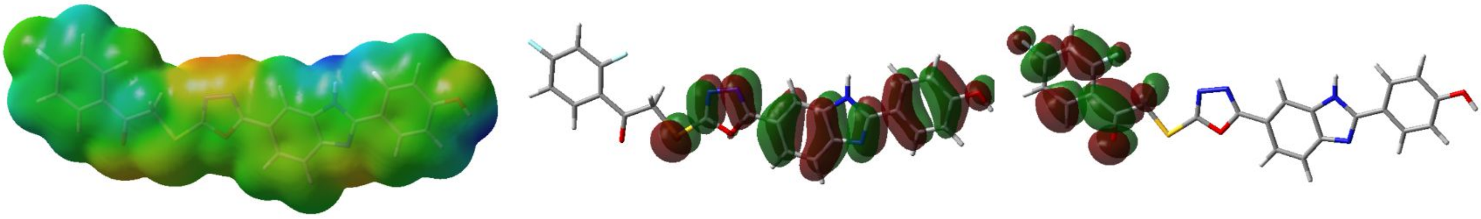

4g

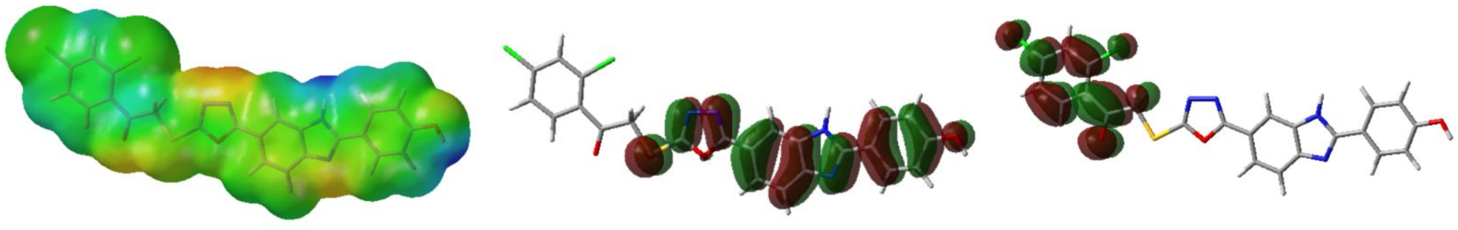

4h

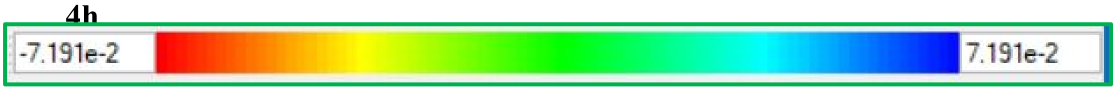

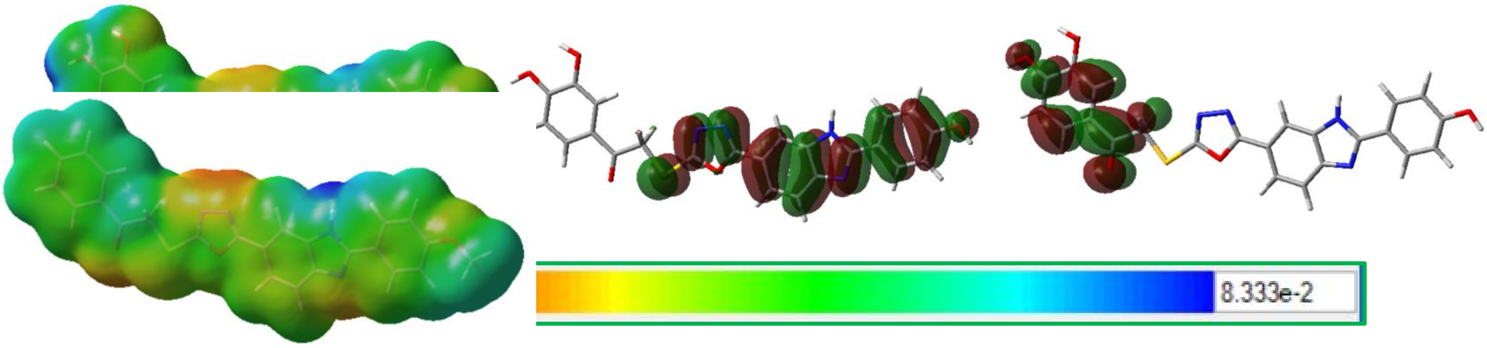

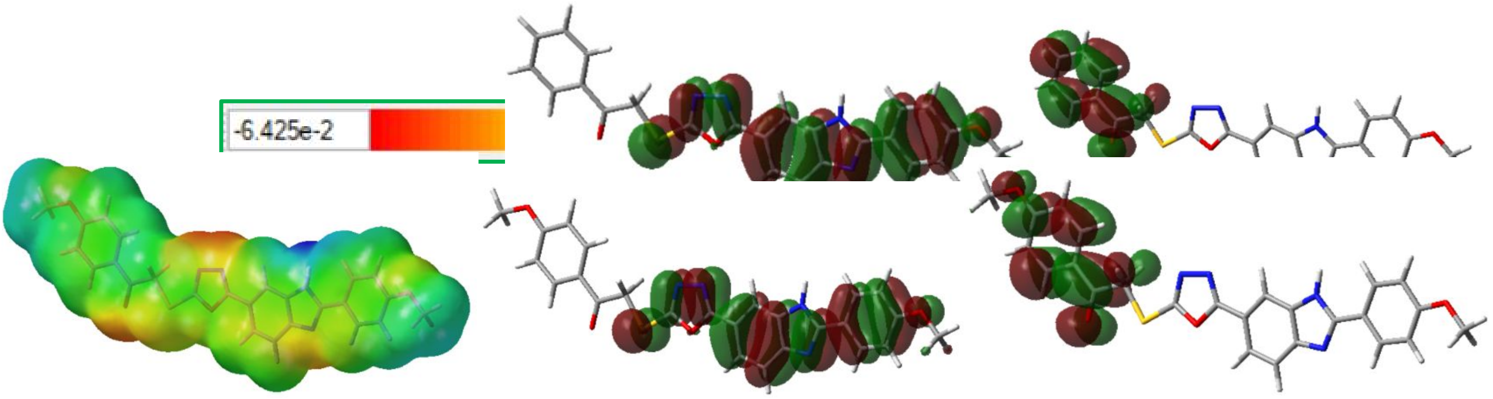

4k

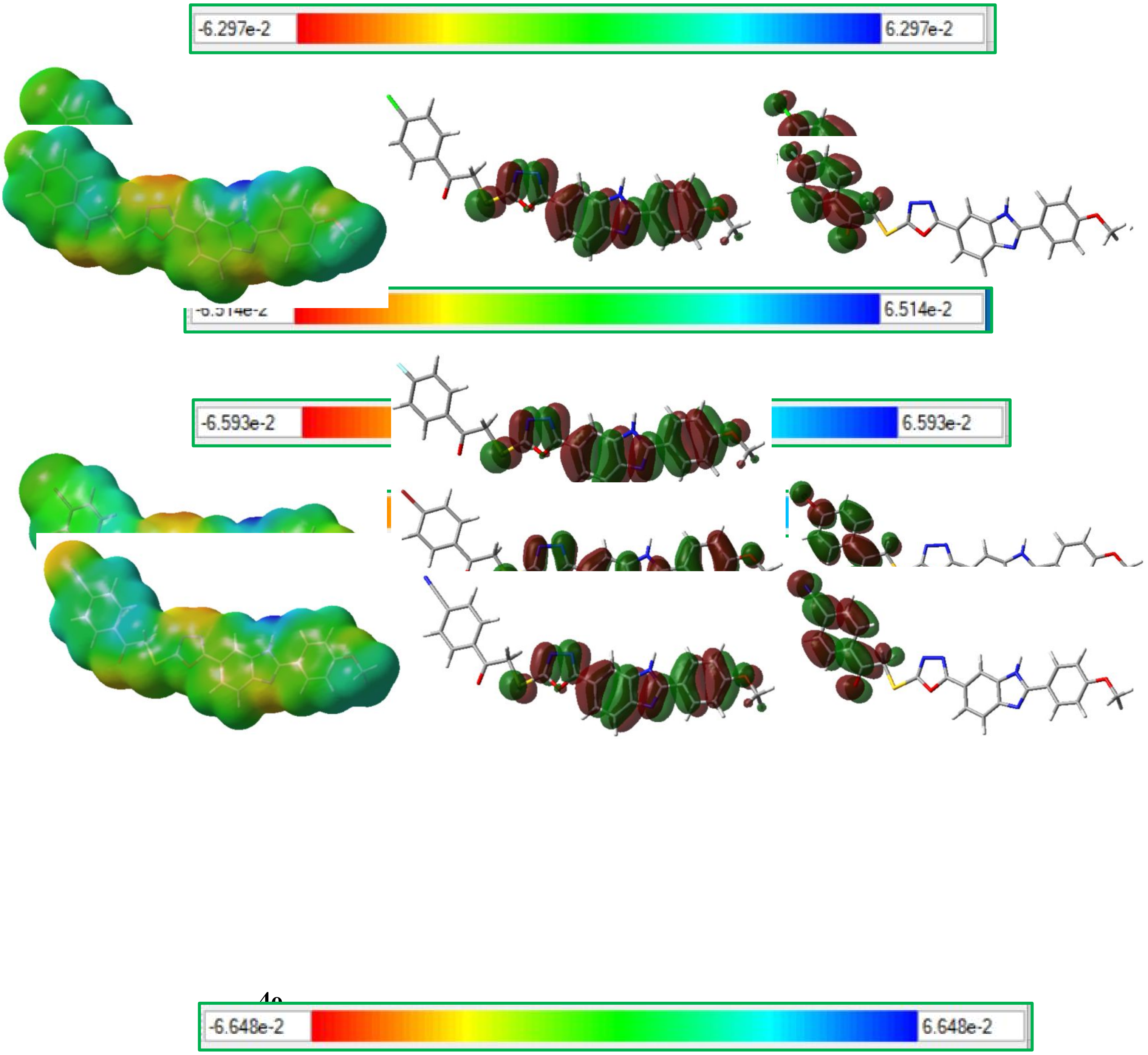

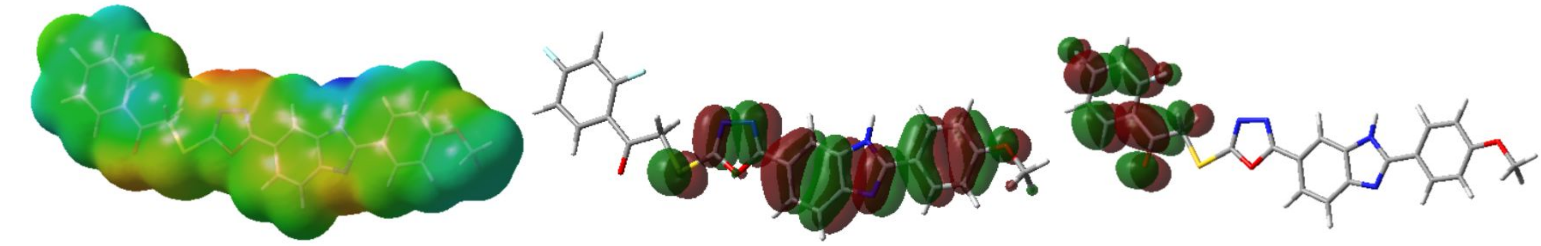

4p

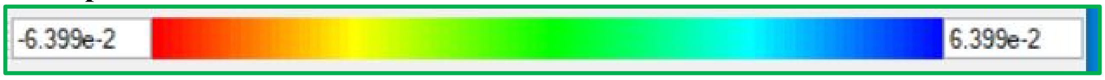

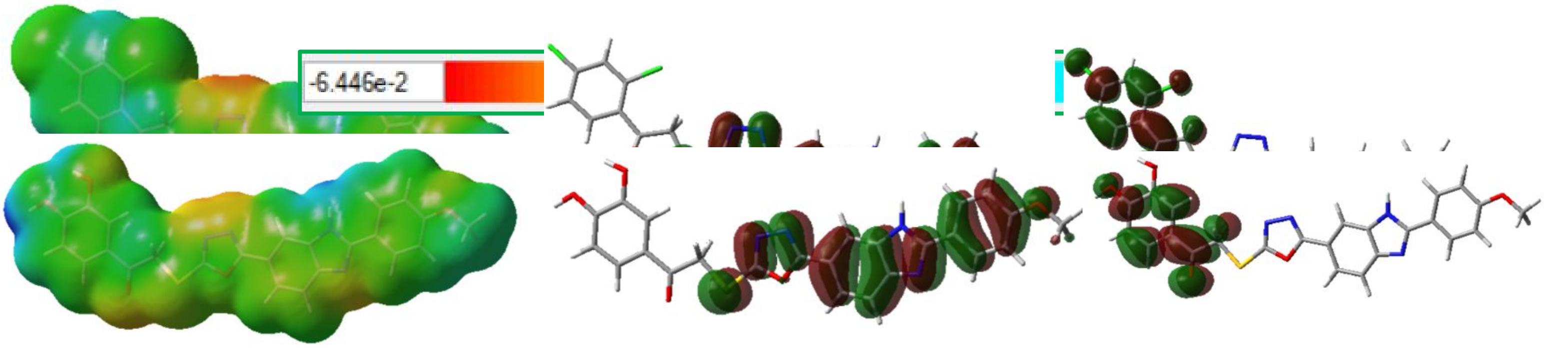

4s

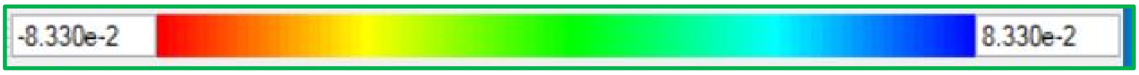

Figure S2. The Molecular Electrostatic Potential (MEP) and HOMO-LUMO diagrams of the compounds **4a-4s** at B3LYP/6-31G(d,p) level. The atom colours are as follows: carbon is represented by grey, nitrogen by blue, oxygen by red, sulfur by yellow, chlorine by green, bromine by dark red, fluorine by cyan and hydrogen by white. The surfaces plotted are those of the 0.0004 electrons/b3 contour of the electronic density.(For **4a** molecule: Color ranges, in au: blue, more positive than 0.07220; green, between 0.07220 and 0; yellow, between 0 and − 0.07220; red, more negative than − 0.07220).

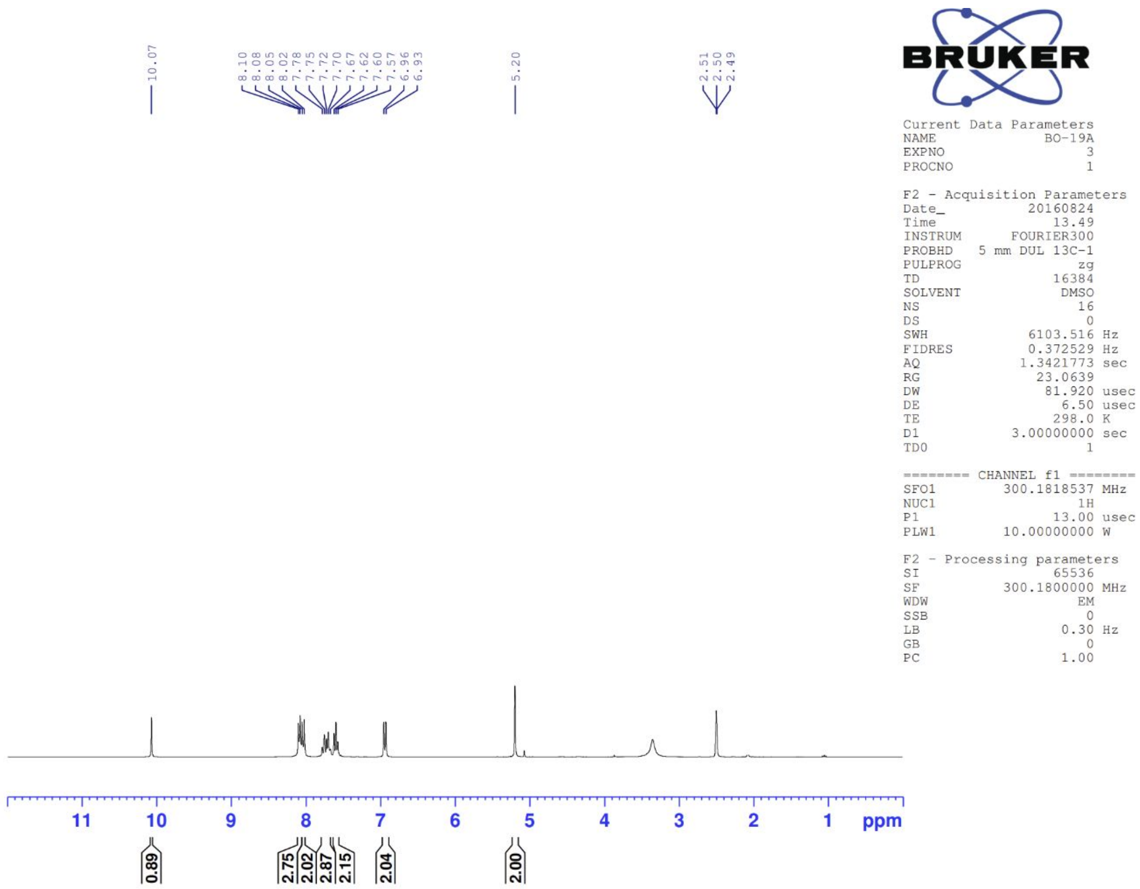

Figure S3. ^{1}H-NMR spectrum of compound **4a**

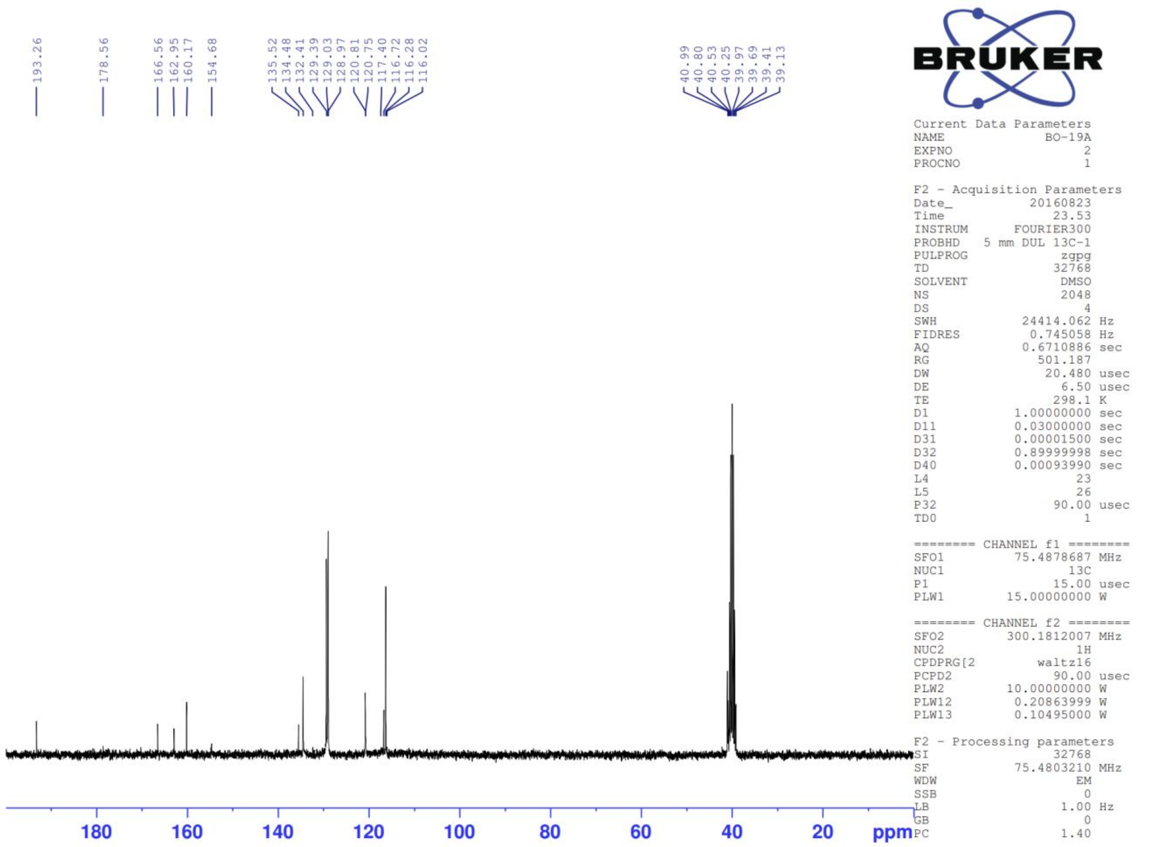

Figure S4. ^{13}C-NMR spectrum of compound **4a**

Data File: C:\LabSolutions\Data\Analiz\uac\BO-19A_24.lcd

Elmt	Val.	Min	Max	Elmt	Val.	Min	Max	Elmt	Val.	Min	Max	Elmt	Val.	Min	Max	Use Adduct
H	1	10	20	O	2	0	5	Cl	1	0	0	I	3	0	0	H
C	4	0	25	F	1	0	0	Br	1	0	1					
N	3	0	5	S	2	0	2	Ru	2	0	0					

Error Margin (ppm):	5	DBE Range:	0.0 - 20.0	Electron Ions:	both
HC Ratio:	unlimited	Apply N Rule:	yes	Use MSn Info:	no
Max Isotopes:	3	Isotope RI (%):	1.00	Isotope Res:	10000
MSn Iso RI (%):	10.00	MSn Logic Mode:	AND	Max Results:	500

Event#: 1 MS(E+) Ret. Time : 5.693 -> 5.773 - 9.947 -> 10.854 Scan# : 855 -> 867 - 1493 -> 1629

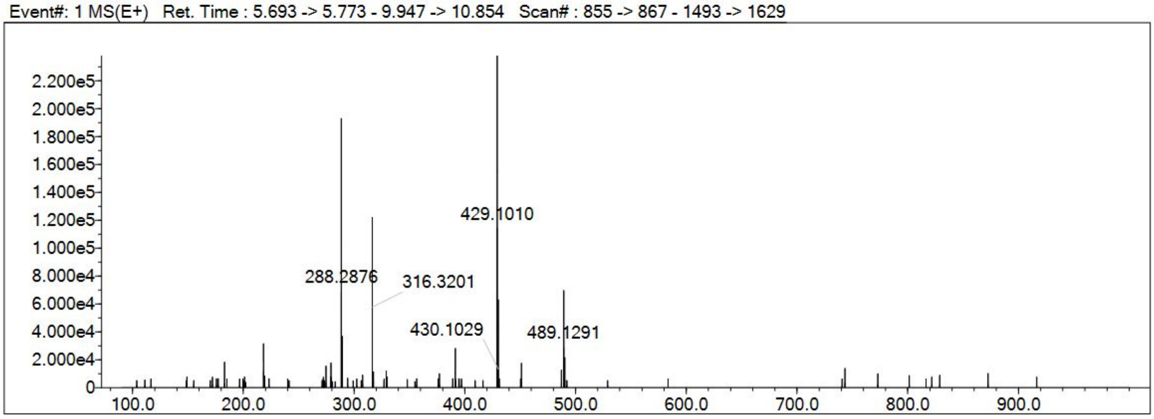

Measured region for 429.1010 m/z

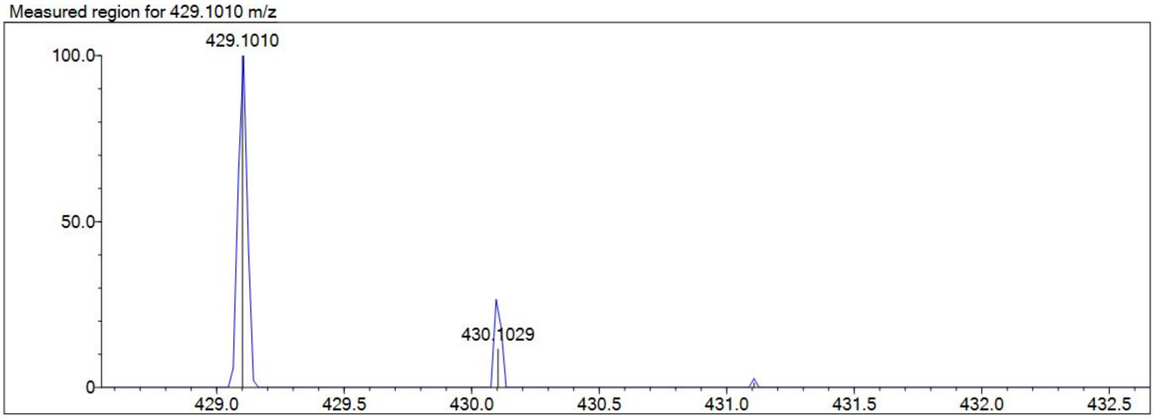

C23 H16 N4 O3 S [M+H]+ : Predicted region for 429.1016 m/z

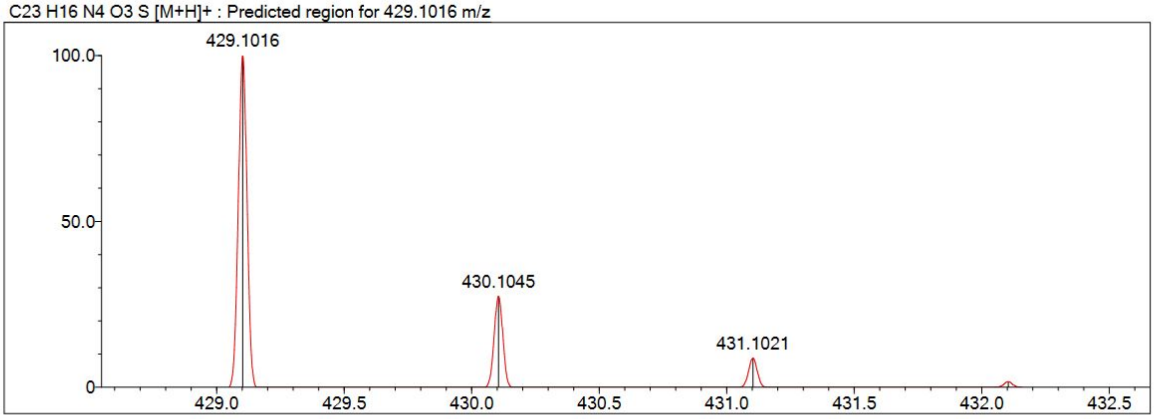

Rank	Score	Formula (M)	Ion	Meas. m/z	Pred. m/z	Df. (mDa)	Df. (ppm)	Iso	DBE
1	59.10	C23 H16 N4 O3 S	[M+H]+	429.1010	429.1016	-0.6	-1.40	59.70	18.0

Figure S5. Mass spectrum of compound **4a**

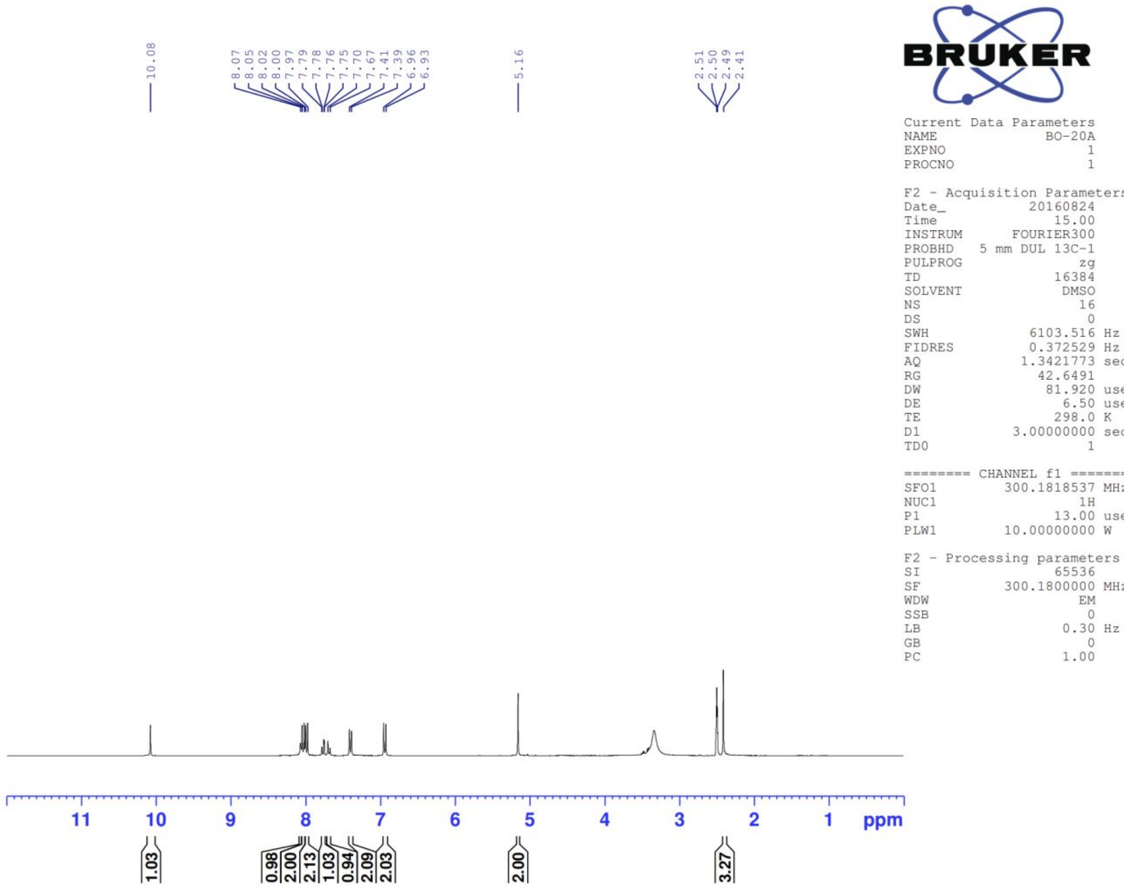

Figure S6. ^{1}H-NMR spectrum of compound **4b**

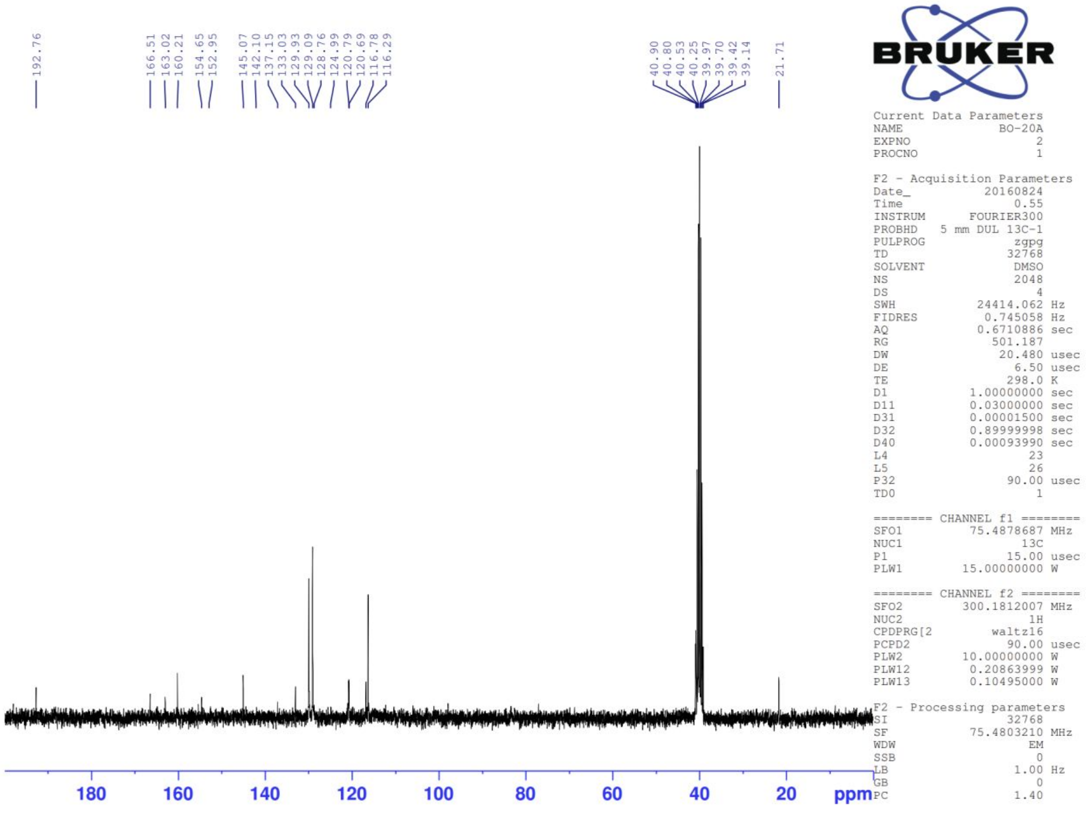

Figure S7. ^{13}C-NMR spectrum of compound **4b**

Data File: C:\LabSolutions\Data\Analiz\uac\BO-20B_26.lcd

Elmt	Val.	Min	Max	Elmt	Val.	Min	Max	Elmt	Val.	Min	Max	Elmt	Val.	Min	Max	Use Adduct
H	1	0	40	O	2	0	5	Cl	1	0	0	I	3	0	0	H
C	4	24	40	F	1	0	1	Br	1	0	0					
N	3	0	8	S	2	0	1	Ru	2	0	0					

Error Margin (ppm):	5	DBE Range:	10.0 - 20.0	Electron Ions:	both
HC Ratio:	unlimited	Apply N Rule:	yes	Use MSn Info:	no
Max Isotopes:	3	Isotope RI (%):	1.00	Isotope Res:	10000
MSn Iso RI (%):	10.00	MSn Logic Mode:	AND	Max Results:	500

Event#: 1 MS(E+) Ret. Time : 5.907 -> 6.040 - 0.080 -> 0.665 Scan# : 887 -> 907 - 13 -> 101

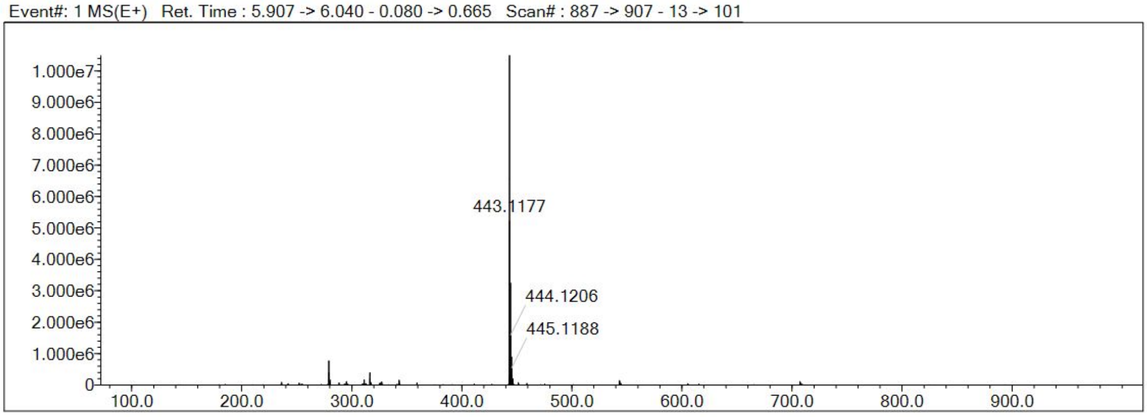

Measured region for 443.1177 m/z

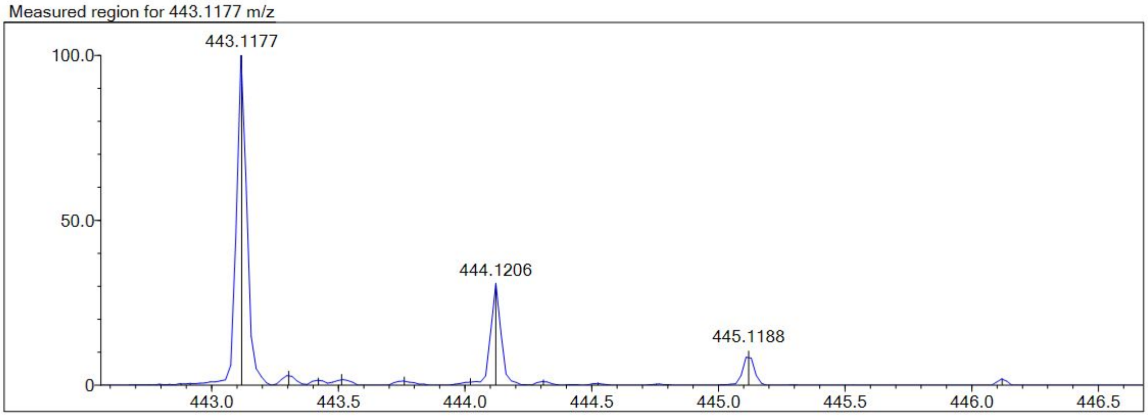

C24 H18 N4 O3 S [M+H]+ : Predicted region for 443.1172 m/z

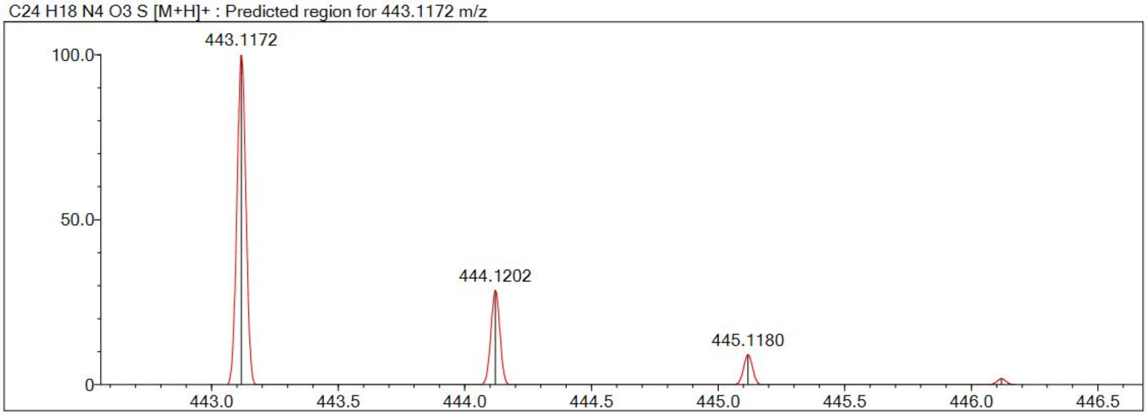

Rank	Score	Formula (M)	Ion	Meas. m/z	Pred. m/z	Df. (mDa)	Df. (ppm)	Iso	DBE
1	94.69	C24 H18 N4 O3 S	[M+H]+	443.1177	443.1172	0.5	1.13	94.99	18.0

Figure S8. Mass spectrum of compound **4b**

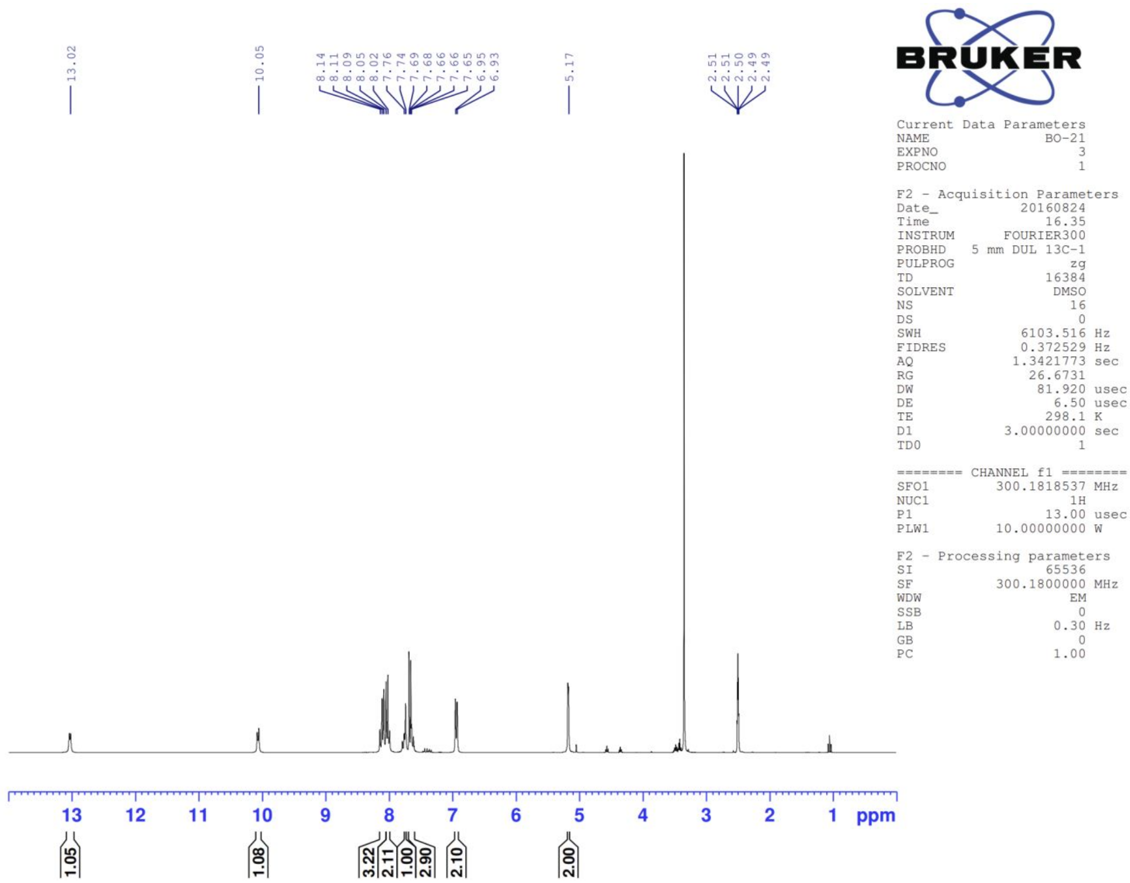

Figure S9. ^{1}H-NMR spectrum of compound **4c**

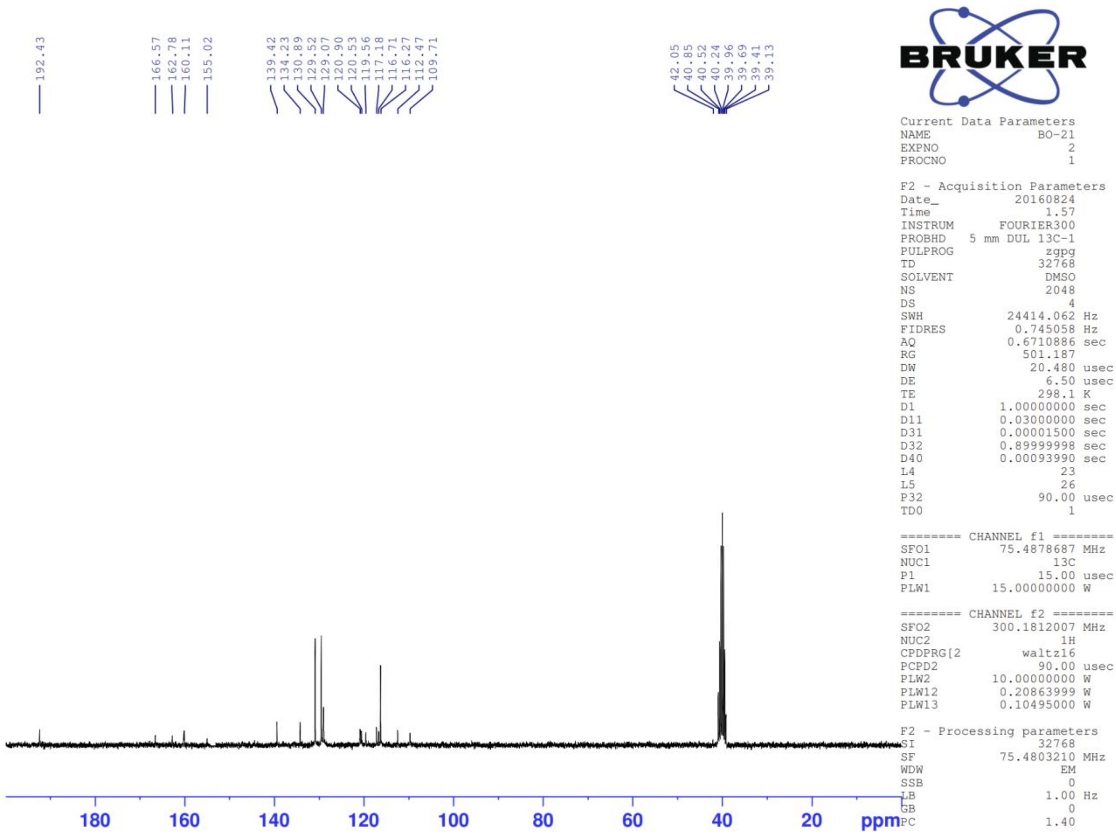

Figure S10. ^{13}C-NMR spectrum of compound **4c**

Data File: C:\LabSolutions\Data\Analiz\uac\BO-21_27.lcd

Elmt	Val.	Min	Max	Elmt	Val.	Min	Max	Elmt	Val.	Min	Max	Elmt	Val.	Min	Max	Use Adduct
H	1	10	20	O	2	0	4	Cl	1	0	2	I	3	0	0	H
C	4	23	23	F	1	0	2	Br	1	0	0					
N	3	0	5	S	2	0	2	Ru	2	0	0					

Error Margin (ppm): 5
HC Ratio: unlimited
Max Isotopes: 3
MSn Iso RI (%): 10.00

DBE Range: 0.0 - 20.0
Apply N Rule: yes
Isotope RI (%): 1.00
MSn Logic Mode: AND

Electron Ions: both
Use MSn Info: no
Isotope Res: 10000
Max Results: 500

Event#: 1 MS(E+) Ret. Time : 6.080 -> 6.213 - 8.387 -> 9.661 Scan# : 913 -> 933 - 1259 -> 1451

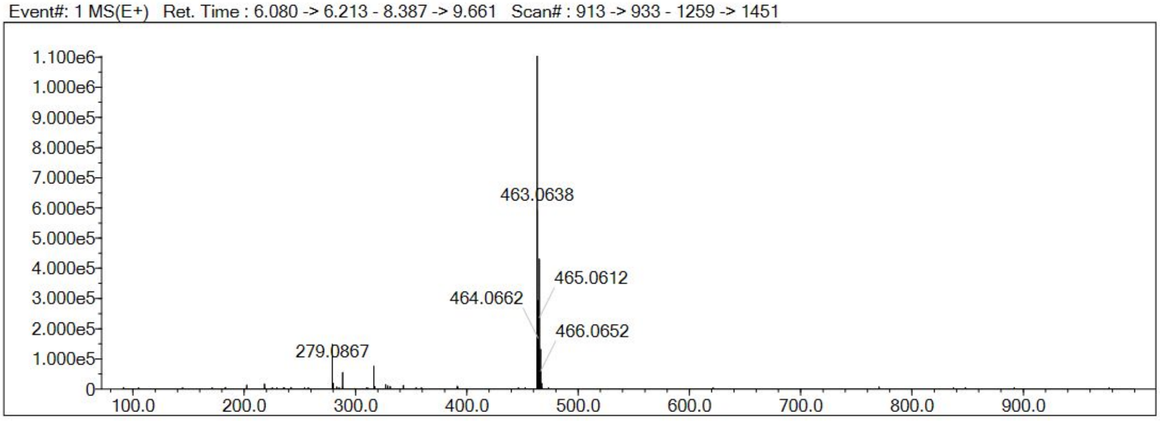

Measured region for 463.0638 m/z

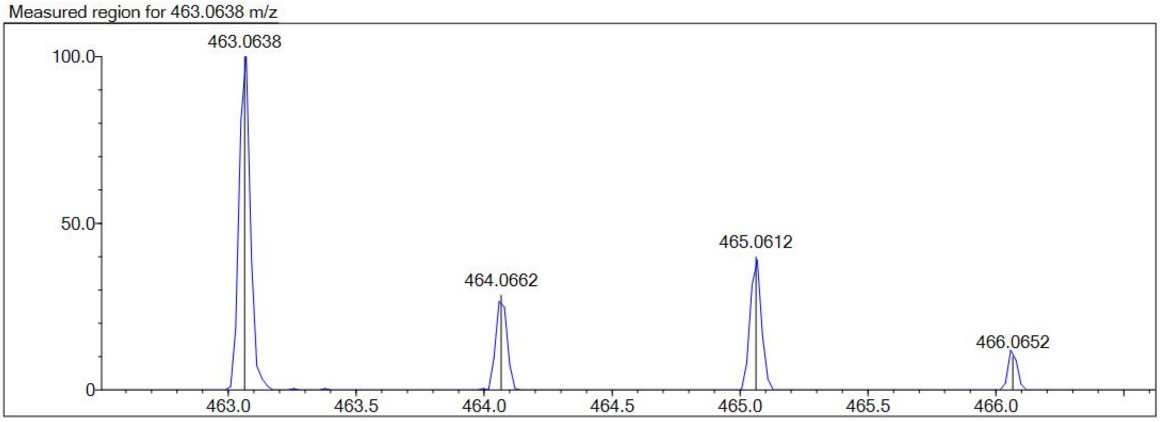

C23 H15 N4 O3 S Cl [M+H]+ : Predicted region for 463.0626 m/z

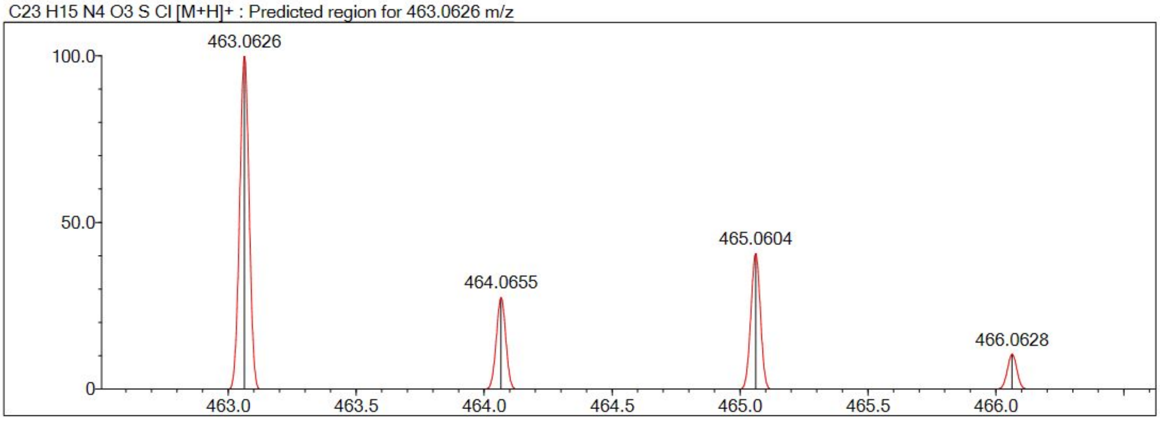

Rank	Score	Formula (M)	Ion	Meas. m/z	Pred. m/z	Df. (mDa)	Df. (ppm)	Iso	DBE
1	82.15	C23 H15 N4 O3 S Cl	[M+H]+	463.0638	463.0626	1.2	2.59	85.55	18.0

Figure S11. Mass spectrum of compound **4c**

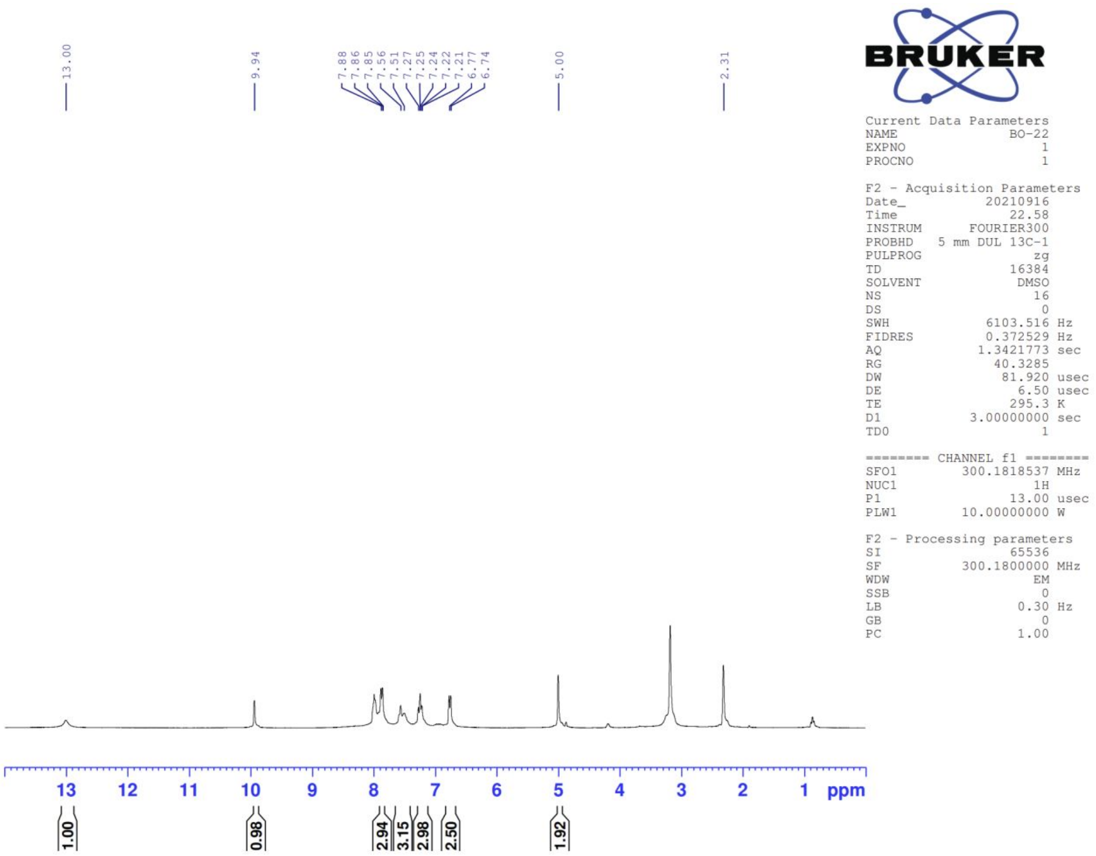

Figure S12. ^{1}H-NMR spectrum of compound **4d**

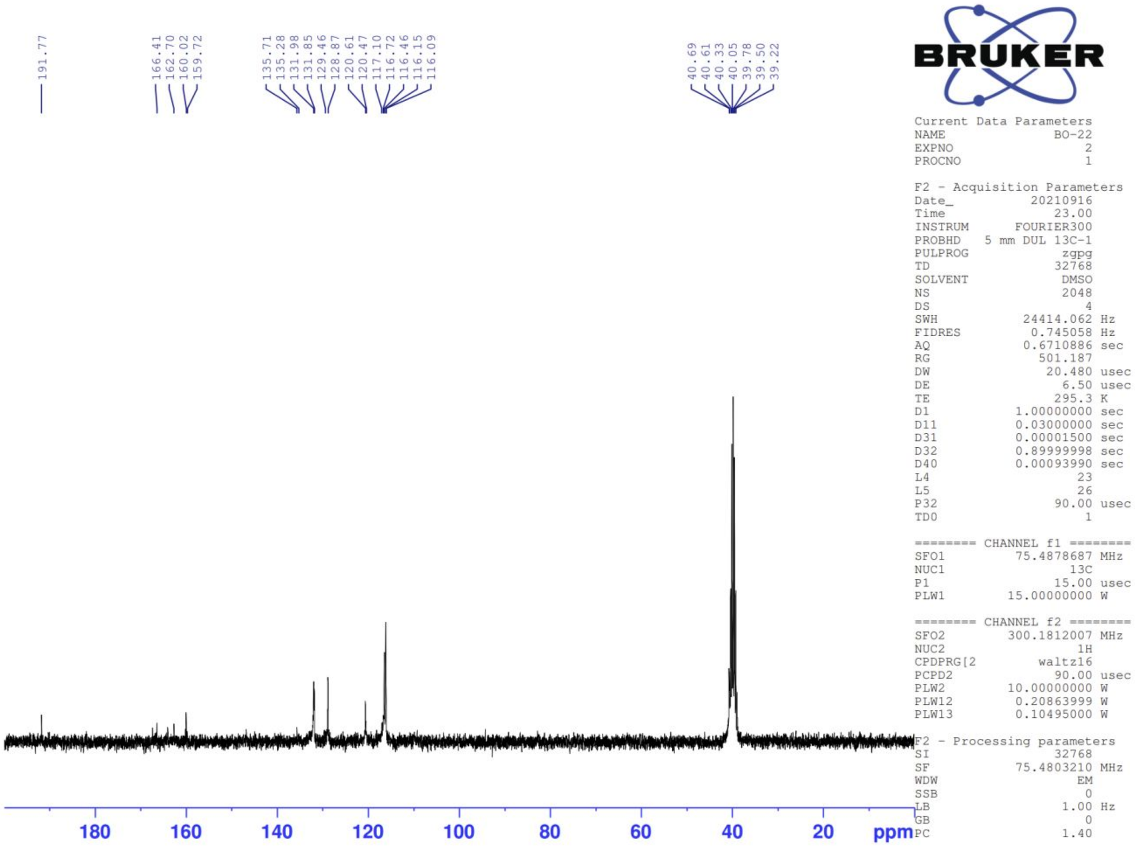

Figure S13. ^{13}C-NMR spectrum of compound **4d**

Formula Predictor Report - BO-22A_28.lcd

Page 1 of 1

Data File: C:\LabSolutions\Data\Analiz\uac\BO-22A_28.lcd

Elmt	Val.	Min	Max	Elmt	Val.	Min	Max	Elmt	Val.	Min	Max	Elmt	Val.	Min	Max	Use Adduct
H	1	10	15	O	2	0	4	Cl	1	0	2	I	3	0	0	H
C	4	23	23	F	1	0	2	Br	1	0	0					
N	3	0	5	S	2	0	2	Ru	2	0	0					

Error Margin (ppm):	5	DBE Range:	0.0 - 20.0	Electron Ions:	both
HC Ratio:	unlimited	Apply N Rule:	yes	Use MSn Info:	no
Max Isotopes:	3	Isotope RI (%):	1.00	Isotope Res:	10000
MSn Iso RI (%):	10.00	MSn Logic Mode:	AND	Max Results:	500

Event#: 1 MS(E+) Ret. Time : 5.733 -> 5.960 - 0.187 -> 1.156 Scan# : 861 -> 895 - 29 -> 175

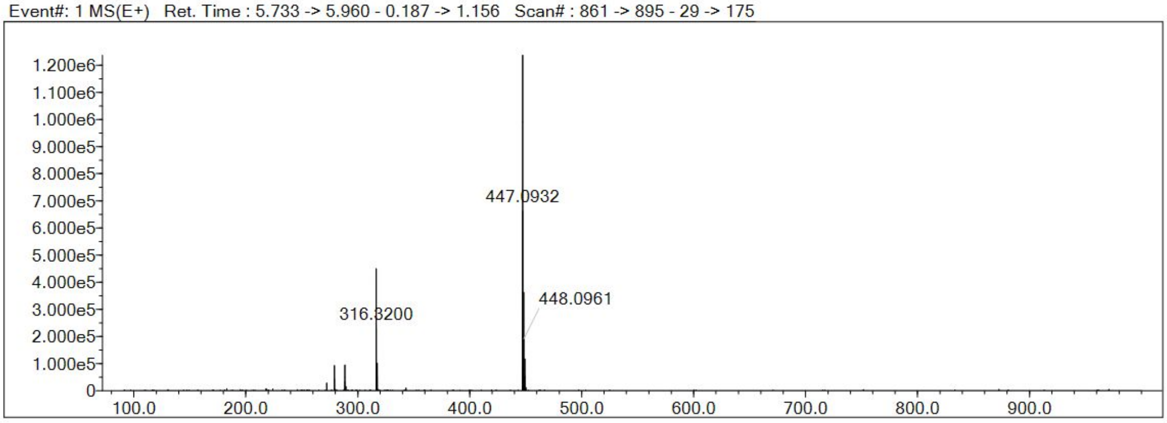

Measured region for 447.0932 m/z

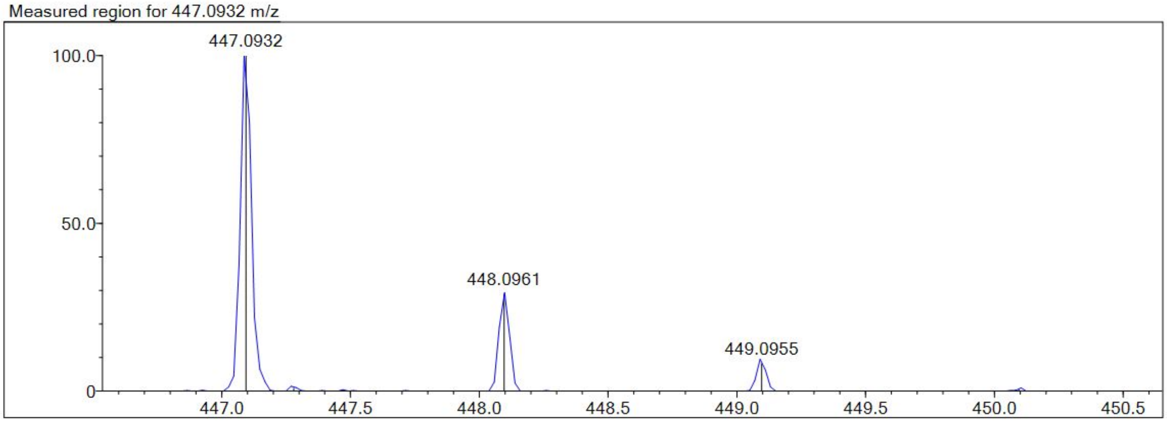

C23 H15 N4 O3 F S [M+H]+ : Predicted region for 447.0922 m/z

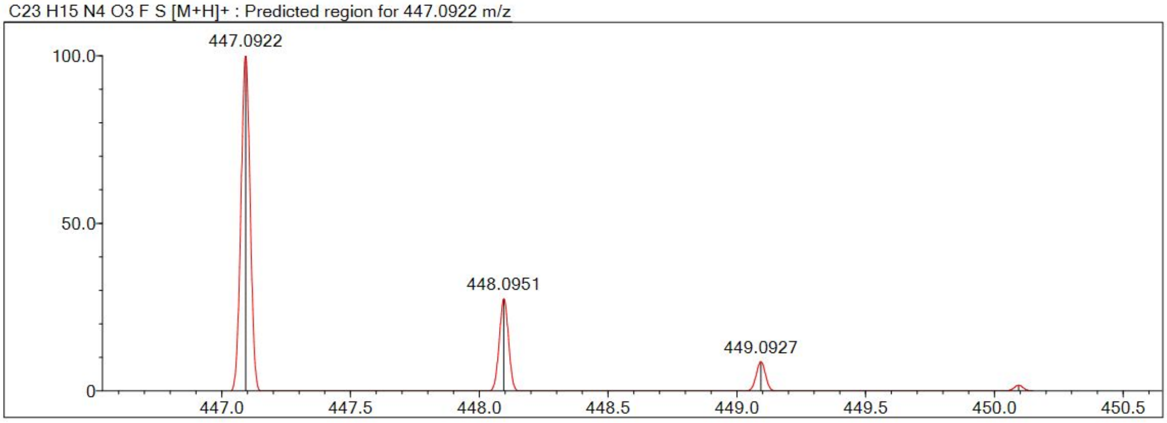

Rank	Score	Formula (M)	Ion	Meas. m/z	Pred. m/z	Df. (mDa)	Df. (ppm)	Iso	DBE
1	75.93	C23 H15 N4 O3 F S	[M+H]+	447.0932	447.0922	1.0	2.24	78.36	18.0

Figure S14. Mass spectrum of compound **4d**

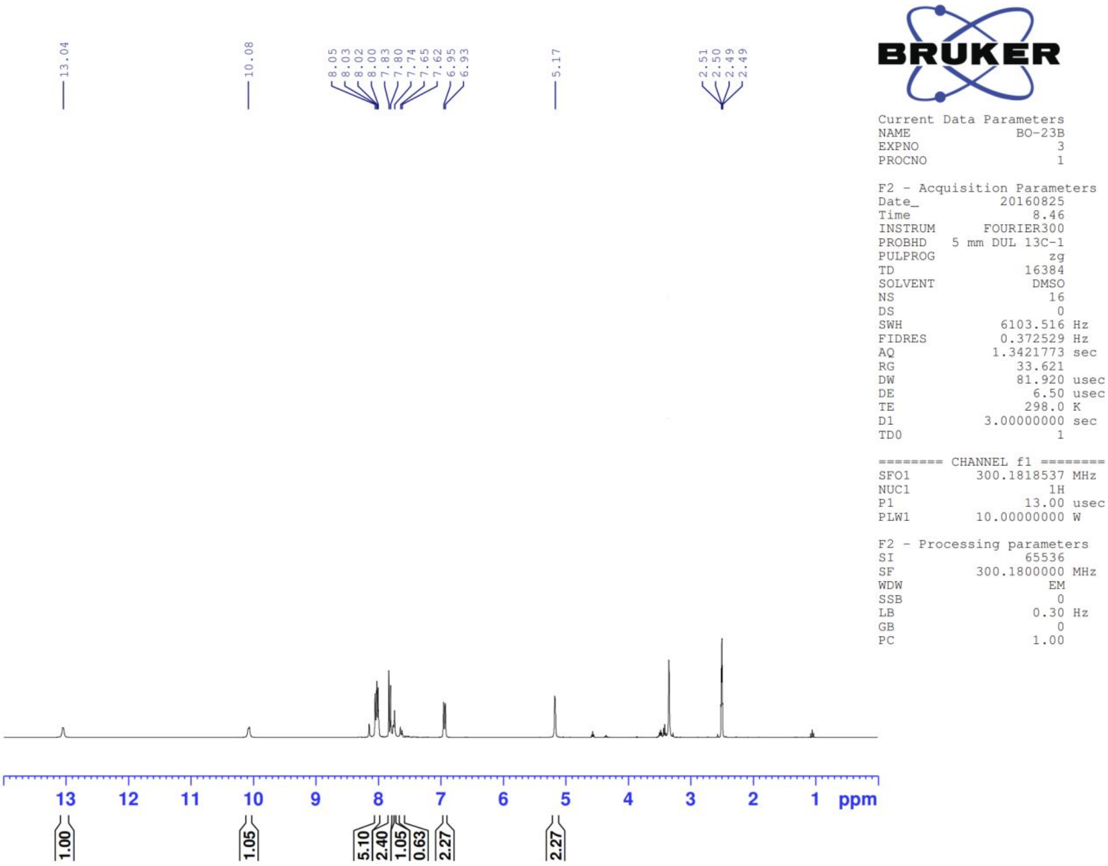

Figure S15. ^{1}H-NMR spectrum of compound **4e**

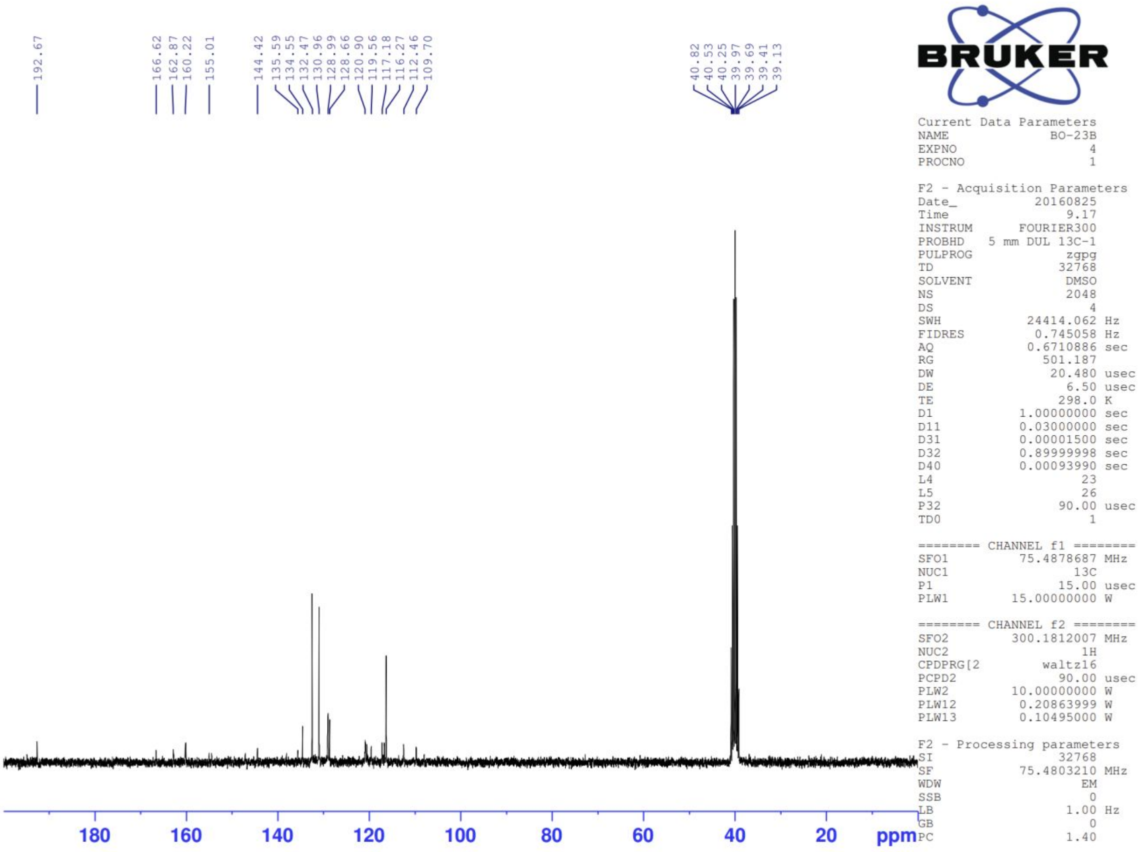

Figure S16. ^{13}C-NMR spectrum of compound **4e**

Data File: C:\LabSolutions\Data\Analiz\uac\BO-23B_32.lcd

Elmt	Val.	Min	Max	Elmt	Val.	Min	Max	Elmt	Val.	Min	Max	Elmt	Val.	Min	Max	Use Adduct
H	1	10	15	O	2	0	4	Cl	1	0	0	I	3	0	0	H
C	4	23	23	F	1	0	0	Br	1	0	1					
N	3	0	5	S	2	0	2	Ru	2	0	0					

Error Margin (ppm):	5	DBE Range:	0.0 - 20.0	Electron Ions:	both
HC Ratio:	unlimited	Apply N Rule:	yes	Use MSn Info:	no
Max Isotopes:	3	Isotope RI (%):	1.00	Isotope Res:	10000
MSn Iso RI (%):	10.00	MSn Logic Mode:	AND	Max Results:	500

Event#: 1 MS(E+) Ret. Time : 6.227 -> 6.333 - 0.107 -> 5.518 Scan# : 935 -> 951 - 17 -> 829

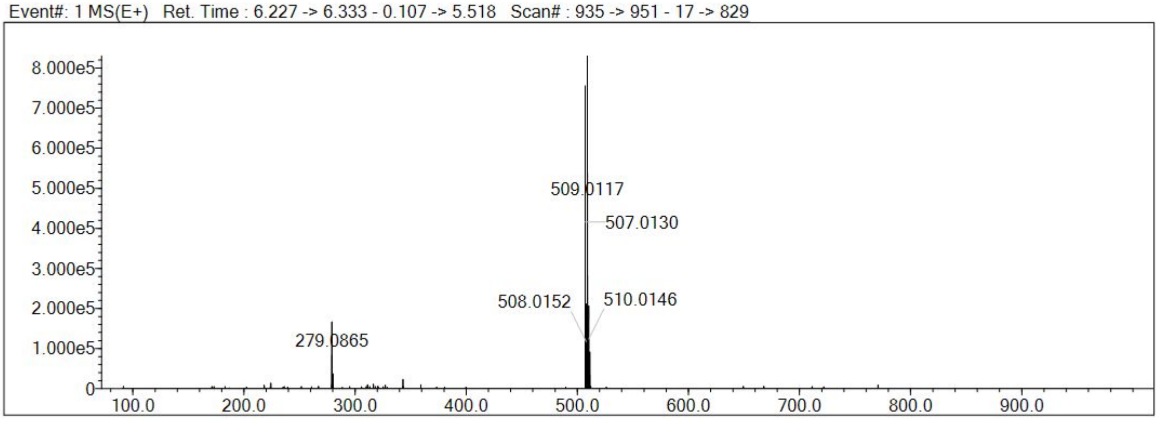

Measured region for 507.0130 m/z

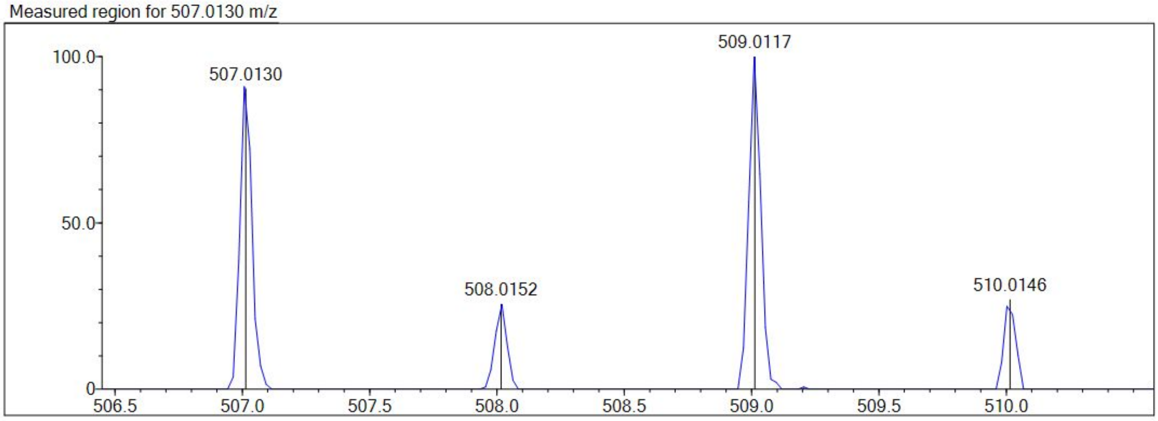

C23 H15 N4 O3 S Br [M+H]+ : Predicted region for 507.0121 m/z

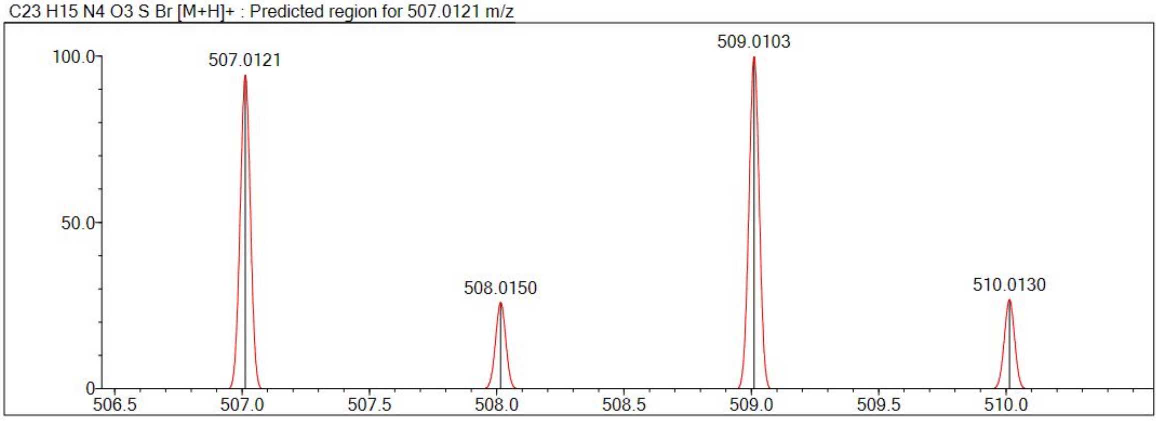

Rank	Score	Formula (M)	Ion	Meas. m/z	Pred. m/z	Df. (mDa)	Df. (ppm)	Iso	DBE
1	79.03	C23 H15 N4 O3 S Br	[M+H]+	507.0130	507.0121	0.9	1.78	80.60	18.0

Figure S17. Mass spectrum of compound **4e**

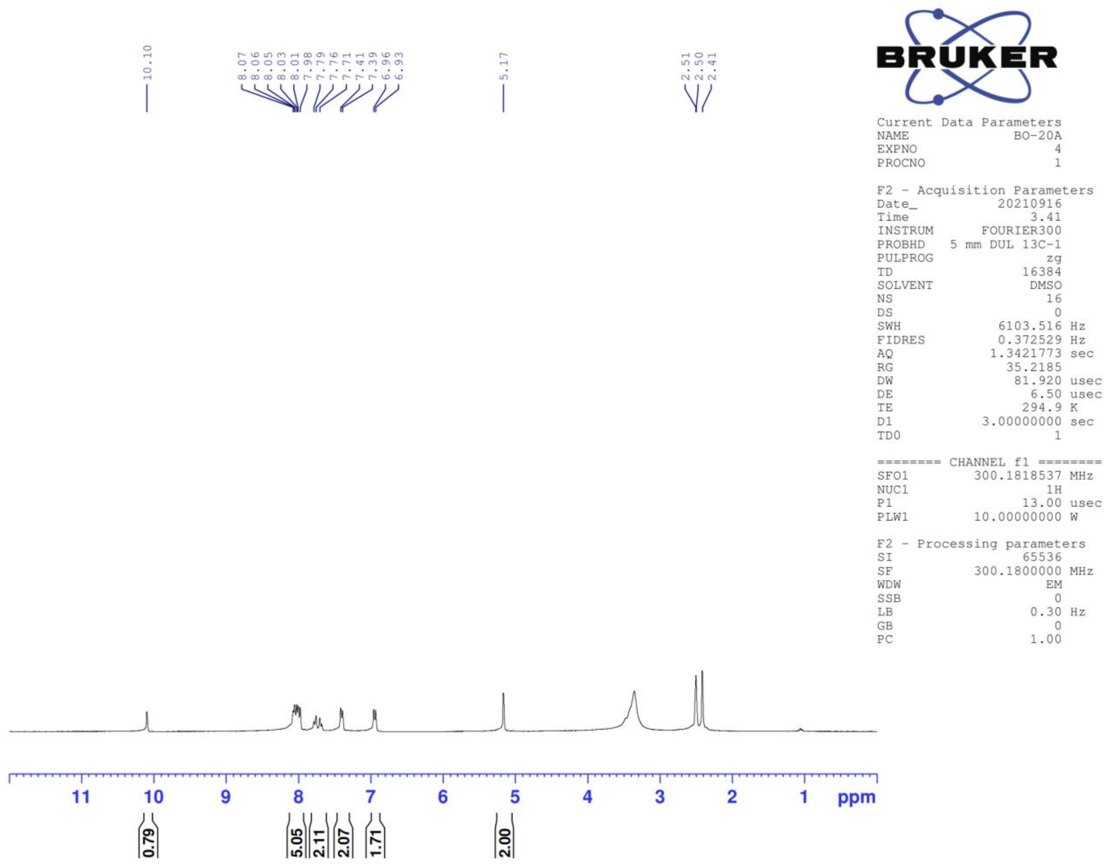

Figure S18. ^{1}H-NMR spectrum of compound **4f**

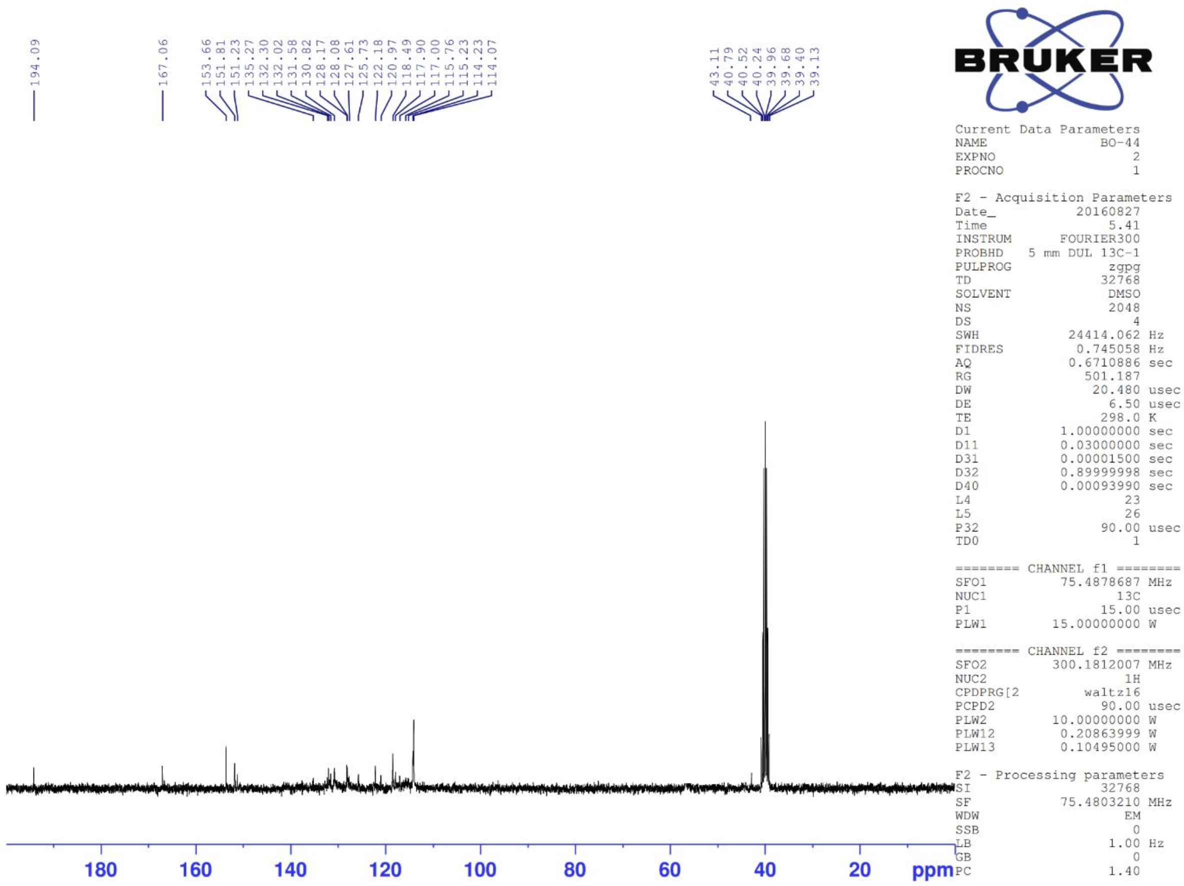

Figure S19. ^{13}C-NMR spectrum of compound **4f**

Formula Predictor Report - BO-24A_33.lcd

Page 1 of 1

Data File: C:\LabSolutions\Data\Analiz\uac\BO-24A_33.lcd

Elmt	Val.	Min	Max	Elmt	Val.	Min	Max	Elmt	Val.	Min	Max	Elmt	Val.	Min	Max	Use Adduct
H	1	10	15	O	2	0	4	Cl	1	0	0	I	3	0	0	H
C	4	23	24	F	1	0	0	Br	1	0	1					
N	3	0	5	S	2	0	2	Ru	2	0	0					

Error Margin (ppm):	5	DBE Range:	0.0 - 20.0	Electron Ions:	both
HC Ratio:	unlimited	Apply N Rule:	yes	Use MSn Info:	no
Max Isotopes:	3	Isotope RI (%):	1.00	Isotope Res:	10000
MSn Iso RI (%):	10.00	MSn Logic Mode:	AND	Max Results:	500

Event#: 1 MS(E+) Ret. Time : 5.773 -> 6.560 - 8.253 -> 9.215 Scan# : 867 -> 985 - 1239 -> 1383

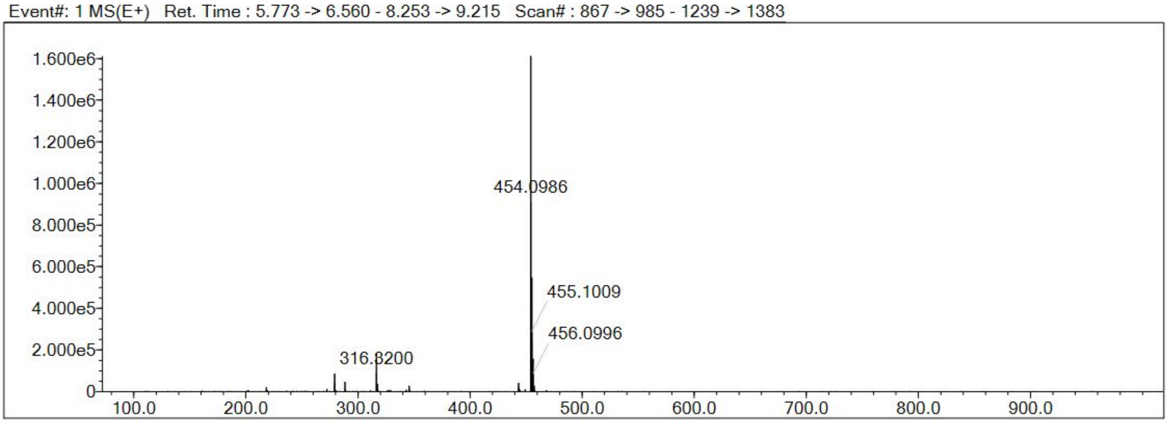

Measured region for 454.0986 m/z

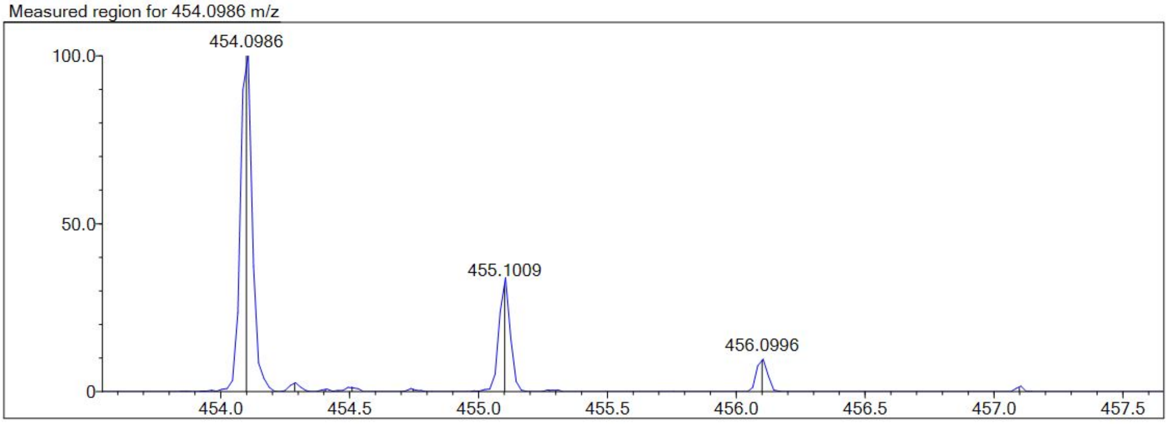

C24 H15 N5 O3 S [M+H]+ : Predicted region for 454.0968 m/z

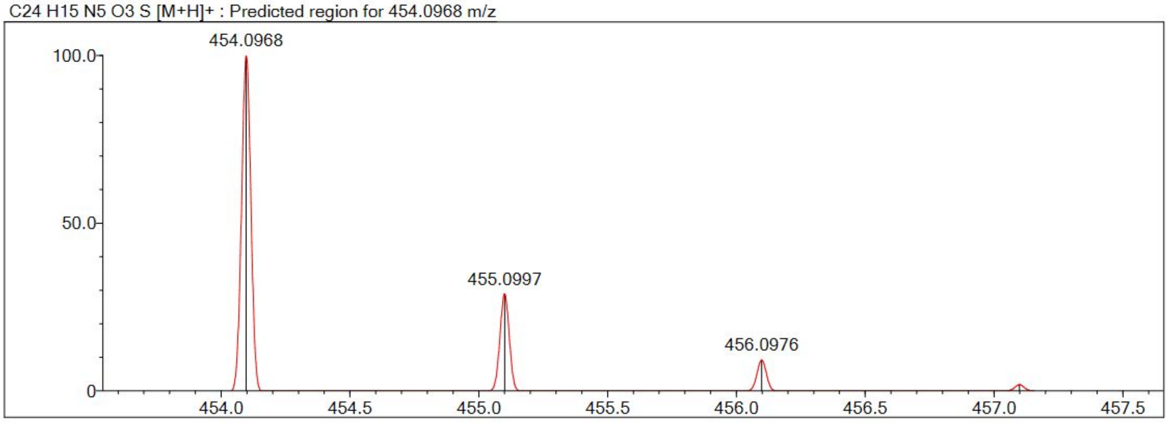

Rank	Score	Formula (M)	Ion	Meas. m/z	Pred. m/z	Df. (mDa)	Df. (ppm)	Iso	DBE
1	87.80	C24 H15 N5 O3 S	[M+H]+	454.0986	454.0968	1.8	3.96	94.82	20.0

Figure S20. Mass spectrum of compound **4f**

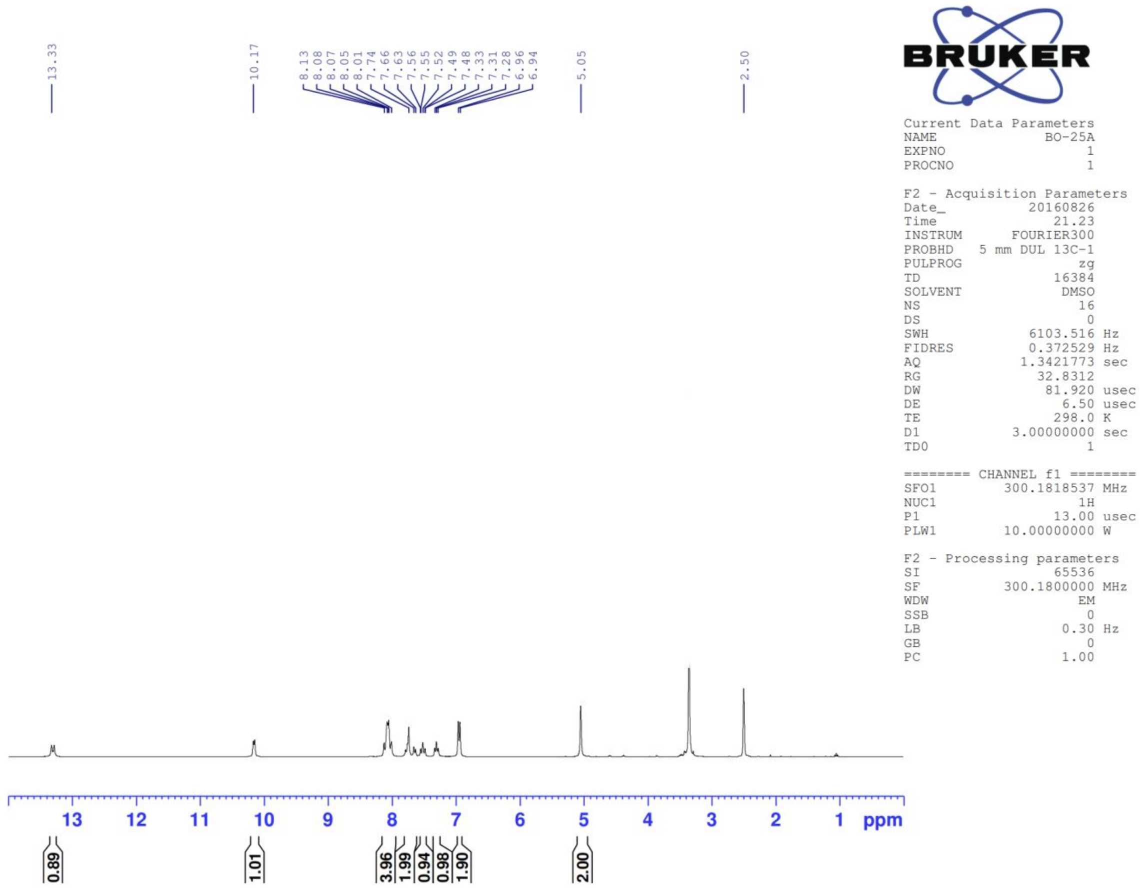

Figure S21. ^{1}H-NMR spectrum of compound **4g**

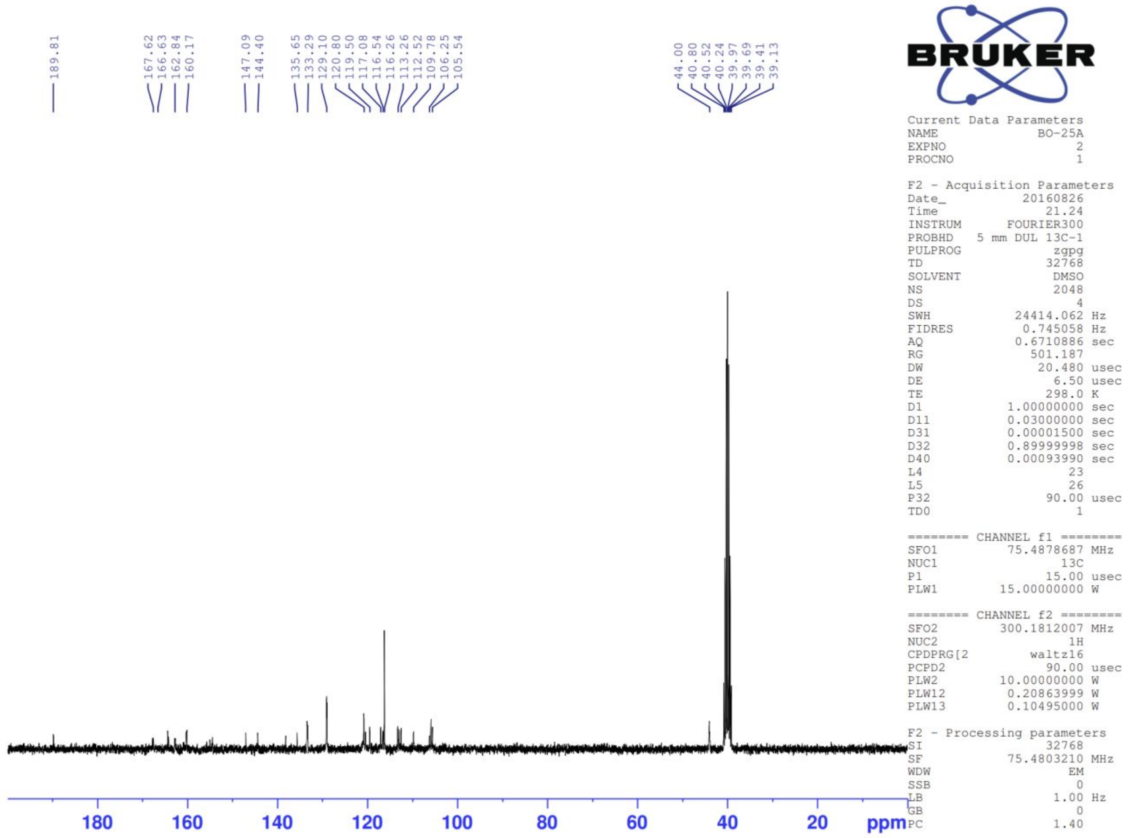

Figure S22. ^{13}C-NMR spectrum of compound **4g**

Data File: C:\LabSolutions\Data\Analiz\uac\BO-25X_1.lcd

Elmt	Val.	Min	Max	Elmt	Val.	Min	Max	Elmt	Val.	Min	Max	Elmt	Val.	Min	Max	Use Adduct
H	1	10	15	O	2	0	4	Cl	1	0	0	I	3	0	0	H
C	4	23	24	F	1	0	2	Br	1	0	1					
N	3	0	5	S	2	0	2	Ru	2	0	0					

Error Margin (ppm):	5	DBE Range:	0.0 - 20.0	Electron Ions:	both
HC Ratio:	unlimited	Apply N Rule:	yes	Use MSn Info:	no
Max Isotopes:	3	Isotope RI (%):	1.00	Isotope Res:	10000
MSn Iso RI (%):	10.00	MSn Logic Mode:	AND	Max Results:	500

Event#: 1 MS(E+) Ret. Time : 6.133 -> 6.560 - 8.080 -> 9.060 Scan# : 921 -> 985 - 1213 -> 1361

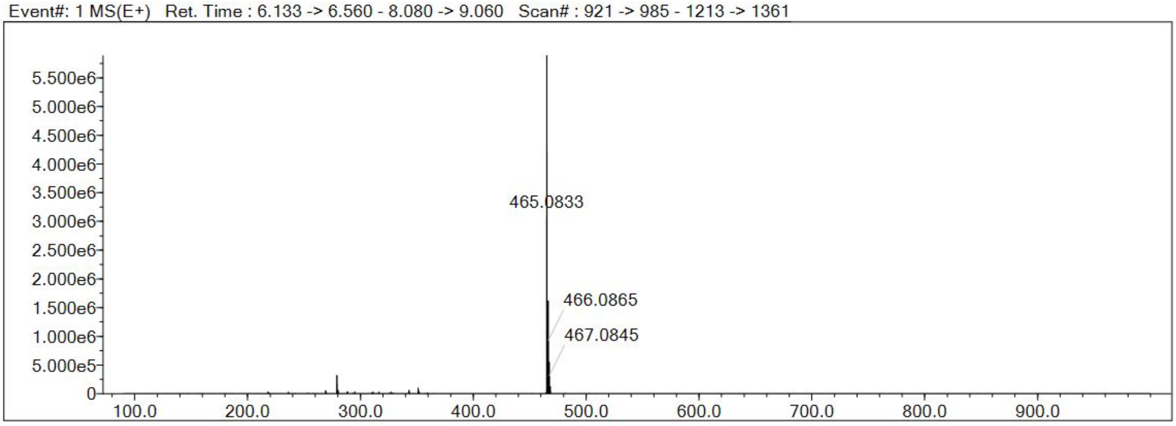

Measured region for 465.0833 m/z

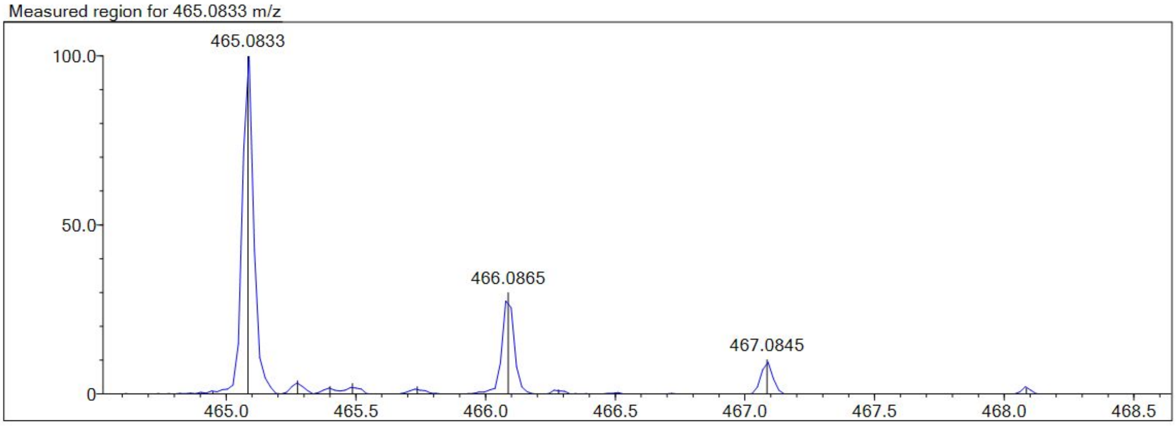

C23 H14 N4 O3 F2 S [M+H]+ : Predicted region for 465.0827 m/z

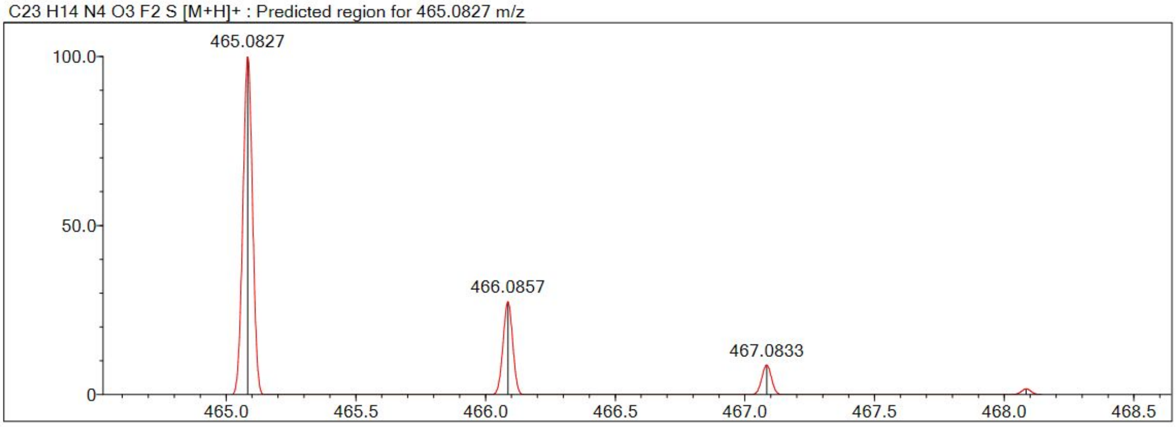

Rank	Score	Formula (M)	Ion	Meas. m/z	Pred. m/z	Df. (mDa)	Df. (ppm)	Iso	DBE
1	89.46	C23 H14 N4 O3 F2 S	[M+H]+	465.0833	465.0827	0.6	1.29	90.11	18.0

Figure S23. Mass spectrum of compound **4g**

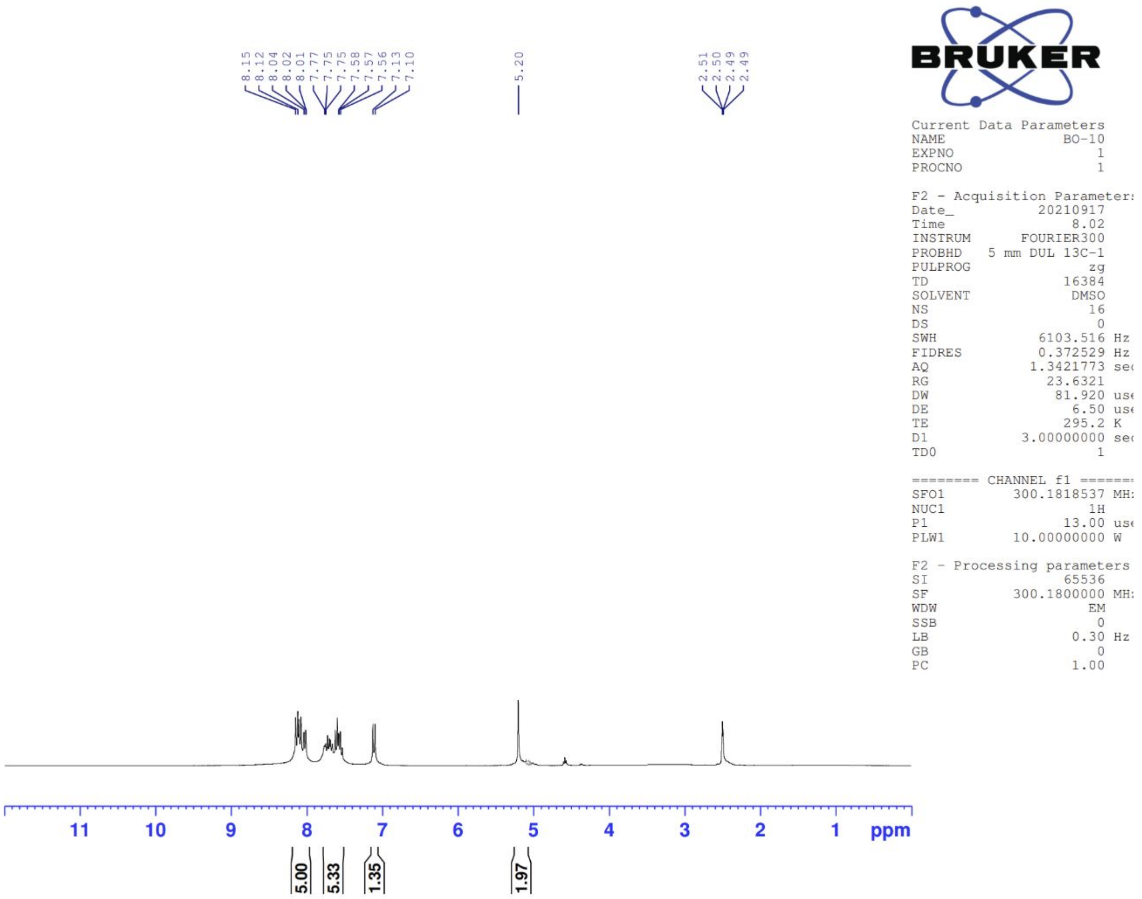

Figure S24. ^{1}H-NMR spectrum of compound **4h**

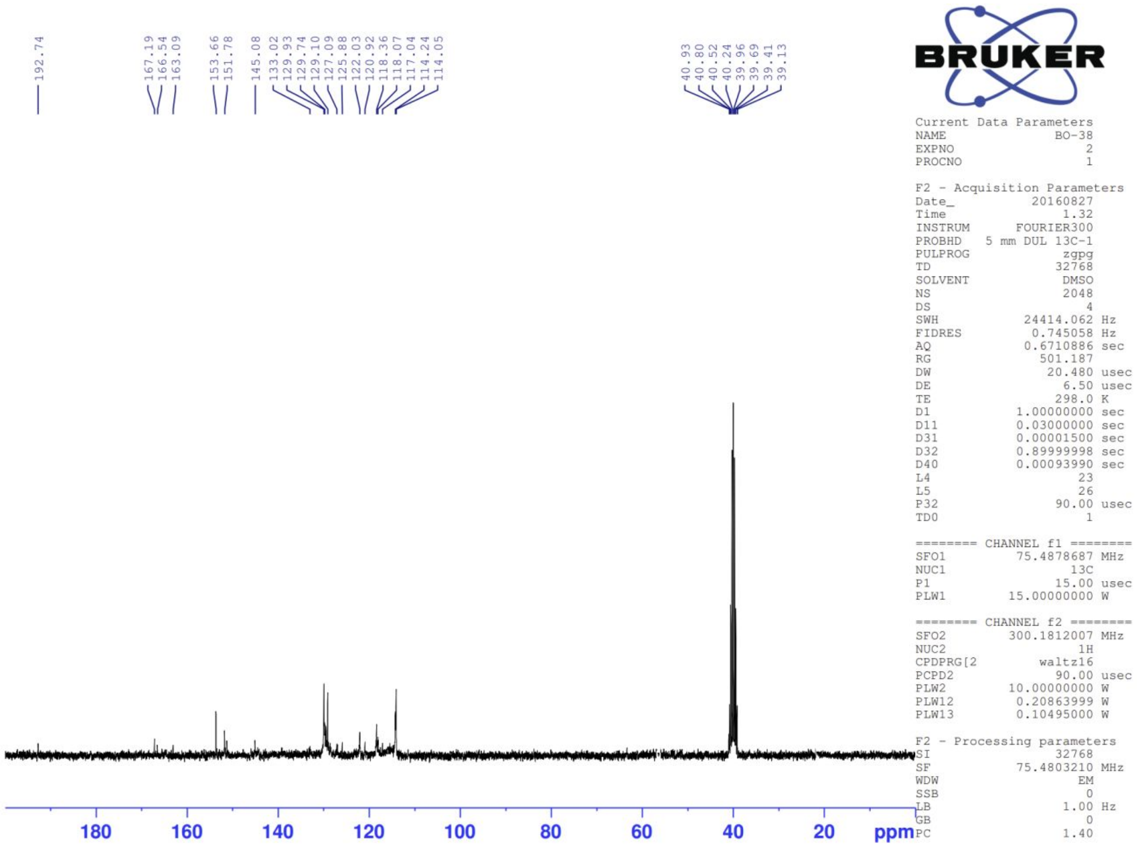

Figure S25. ^{13}C-NMR spectrum of compound **4h**

Data File: C:\LabSolutions\Data\Analiz\uac\BO-26_1.lcd

Elmt	Val.	Min	Max	Elmt	Val.	Min	Max	Elmt	Val.	Min	Max	Elmt	Val.	Min	Max	Use Adduct
H	1	10	15	O	2	0	4	Cl	1	2	2	I	3	0	0	H
C	4	23	24	F	1	0	2	Br	1	0	1					
N	3	0	5	S	2	0	2	Ru	2	0	0					

Error Margin (ppm):	5	DBE Range:	0.0 - 20.0	Electron Ions:	both
HC Ratio:	unlimited	Apply N Rule:	yes	Use MSn Info:	no
Max Isotopes:	3	Isotope RI (%):	1.00	Isotope Res:	10000
MSn Iso RI (%):	10.00	MSn Logic Mode:	AND	Max Results:	500

Event#: 1 MS(E+) Ret. Time : 6.373 -> 6.533 - 7.107 -> 9.297 Scan# : 957 -> 981 - 1067 -> 1395

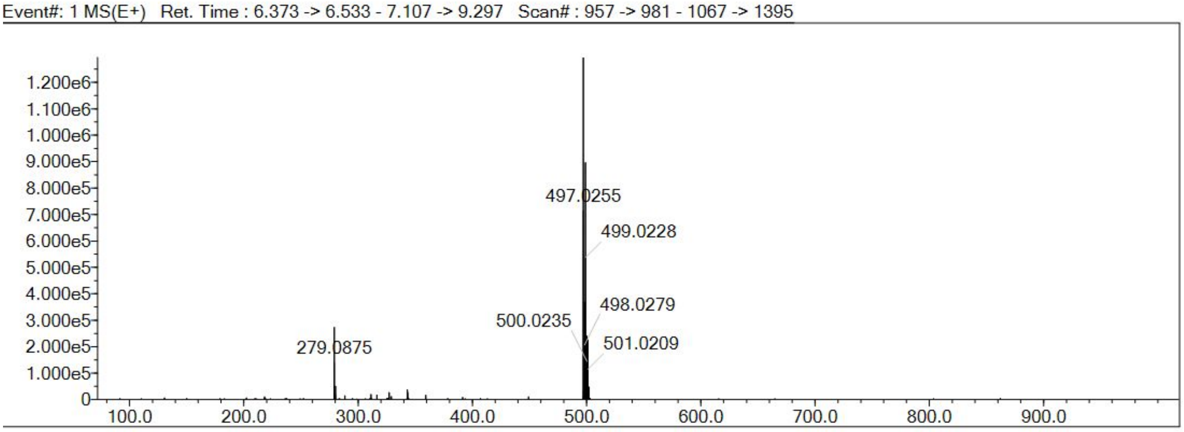

Measured region for 497.0255 m/z

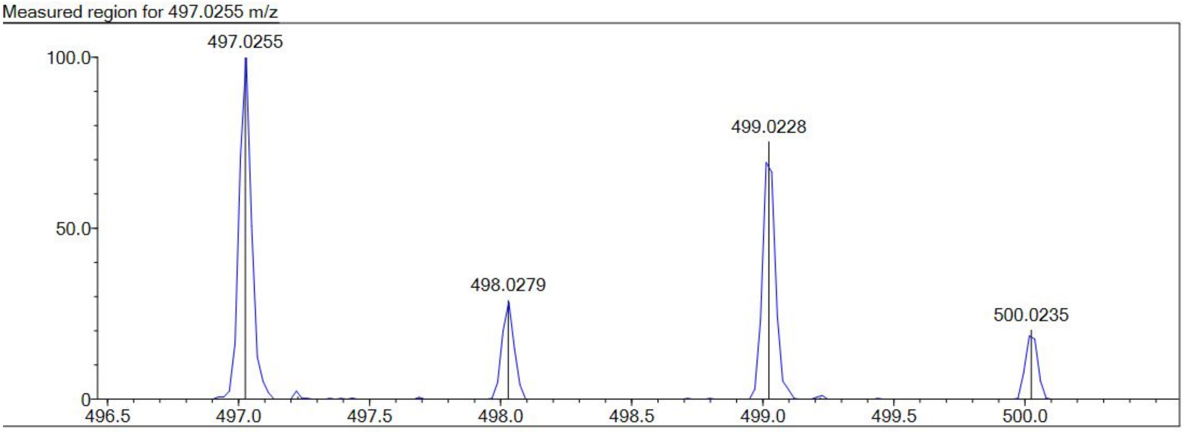

C23 H14 N4 O3 S Cl2 [M+H]+ : Predicted region for 497.0236 m/z

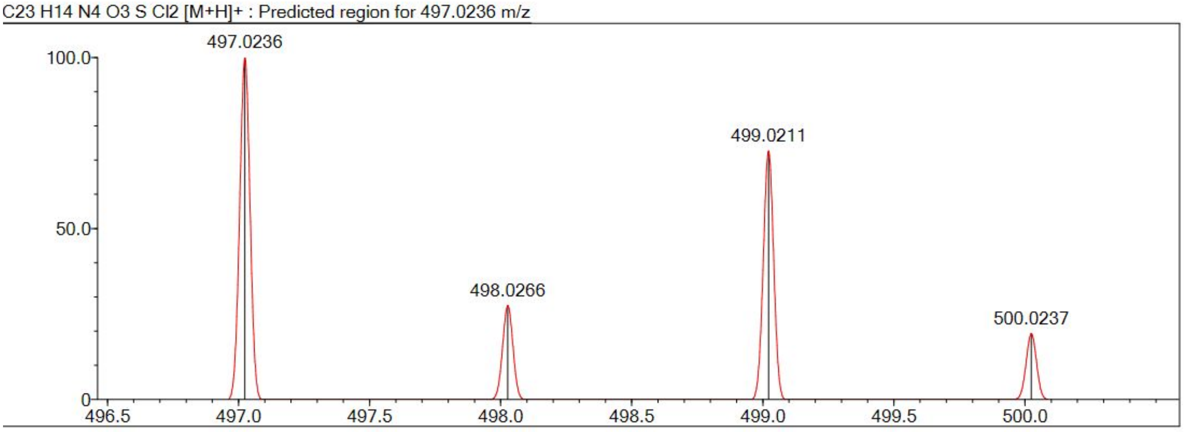

Rank	Score	Formula (M)	Ion	Meas. m/z	Pred. m/z	Df. (mDa)	Df. (ppm)	Iso	DBE
1	79.28	C23 H14 N4 O3 S Cl2	[M+H]+	497.0255	497.0236	1.9	3.82	85.29	18.0

Figure S26. Mass spectrum of compound **4h**

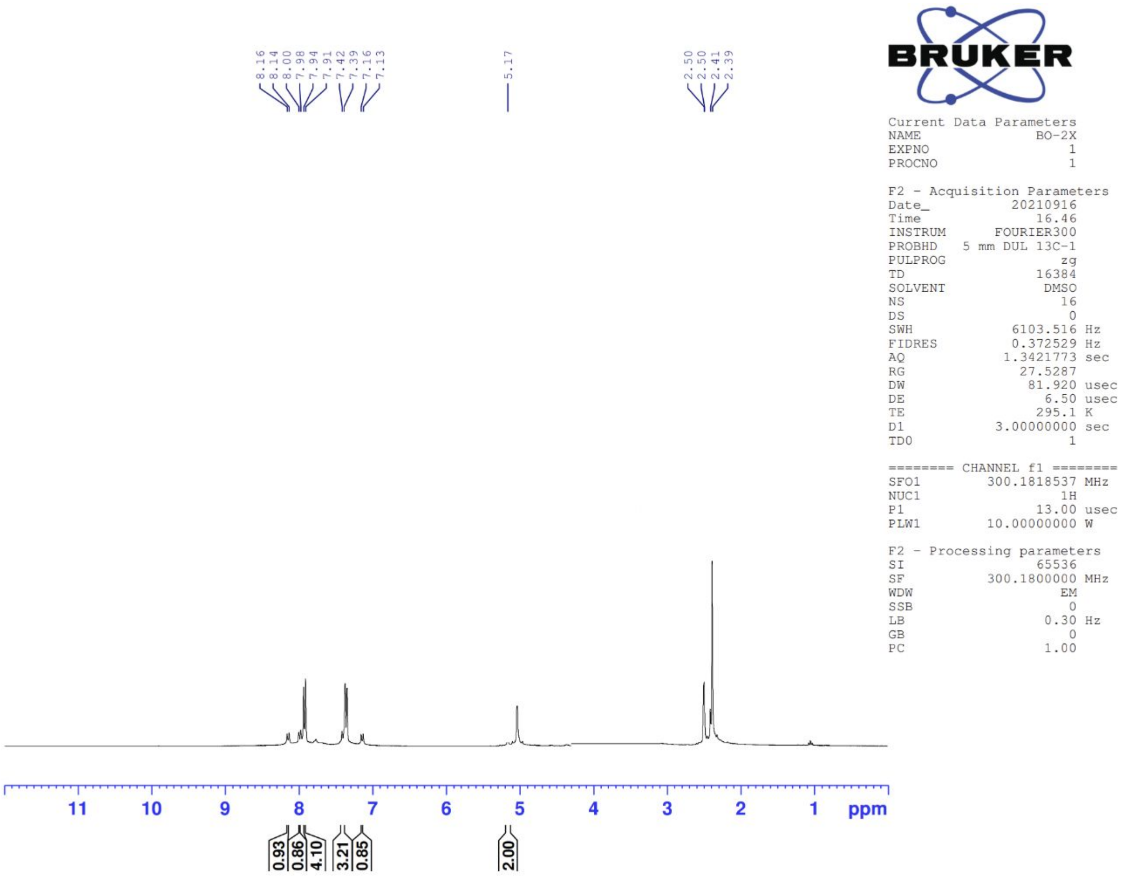

Figure S27. ^{1}H-NMR spectrum of compound **4i**

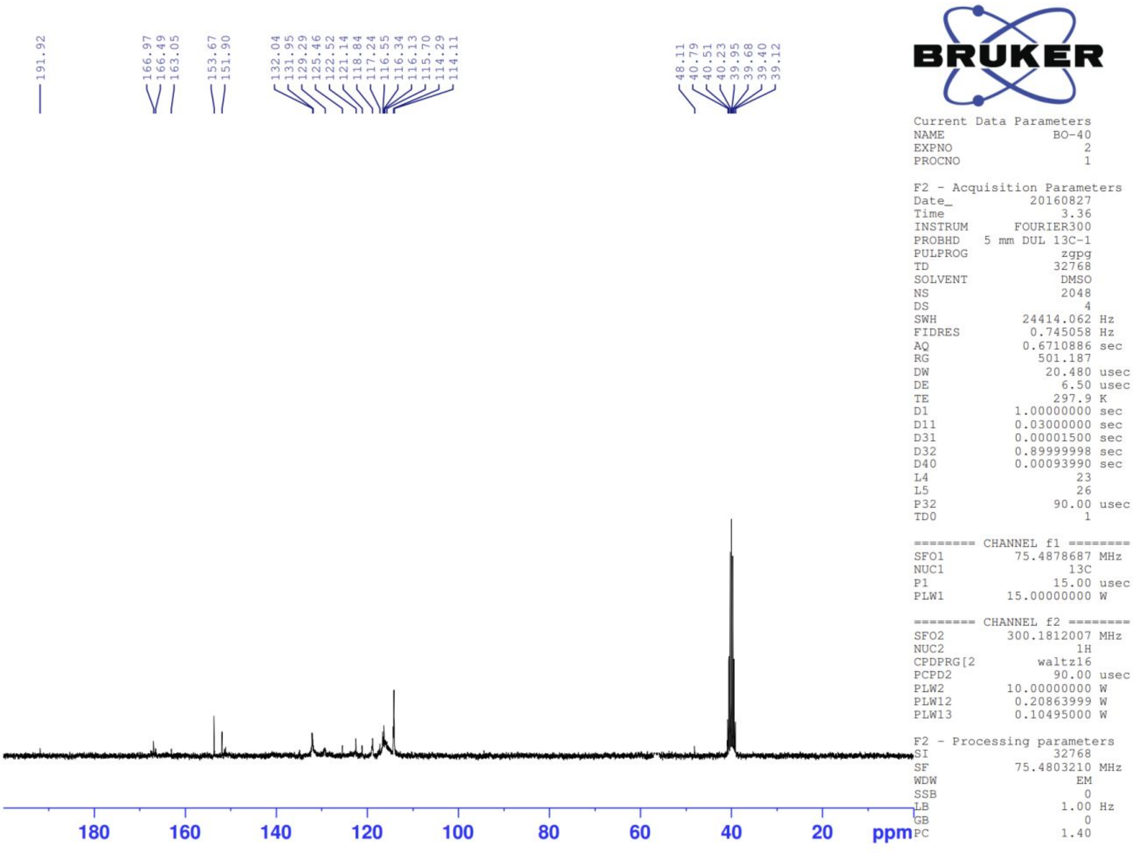

Figure S28. ^{13}C-NMR spectrum of compound **4i**

Data File: C:\LabSolutions\Data\Analiz\uac\BO-27A_2.lcd

Elmt	Val.	Min	Max	Elmt	Val.	Min	Max	Elmt	Val.	Min	Max	Elmt	Val.	Min	Max	Use Adduct
H	1	10	20	O	2	5	5	Cl	1	0	0	I	3	0	0	H
C	4	0	24	F	1	0	2	Br	1	0	1					
N	3	0	5	S	2	0	2	Ru	2	0	0					

Error Margin (ppm):	5	DBE Range:	0.0 - 20.0	Electron Ions:	both
HC Ratio:	unlimited	Apply N Rule:	yes	Use MSn Info:	no
Max Isotopes:	3	Isotope RI (%):	1.00	Isotope Res:	10000
MSn Iso RI (%):	10.00	MSn Logic Mode:	AND	Max Results:	500

Event#: 1 MS(E+) Ret. Time : 4.840 -> 5.053 - 8.467 -> 9.461 Scan# : 727 -> 759 - 1271 -> 1421

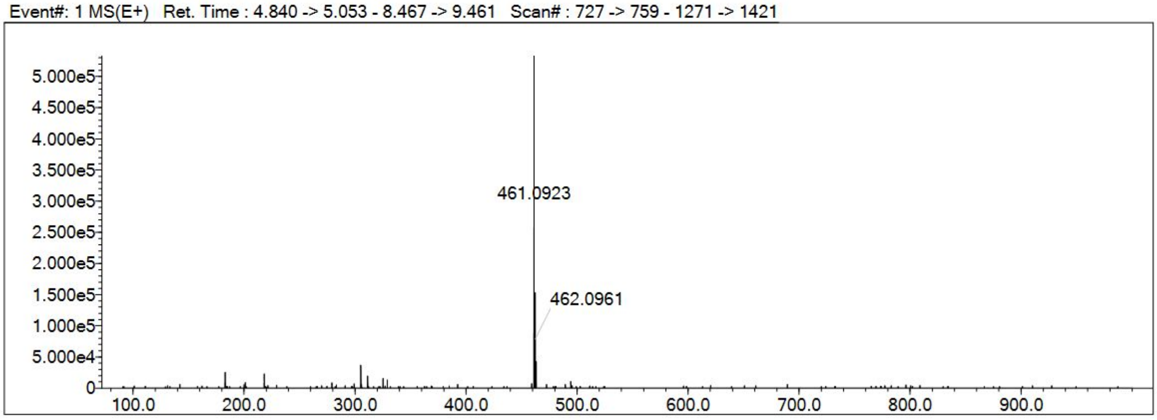

Measured region for 461.0923 m/z

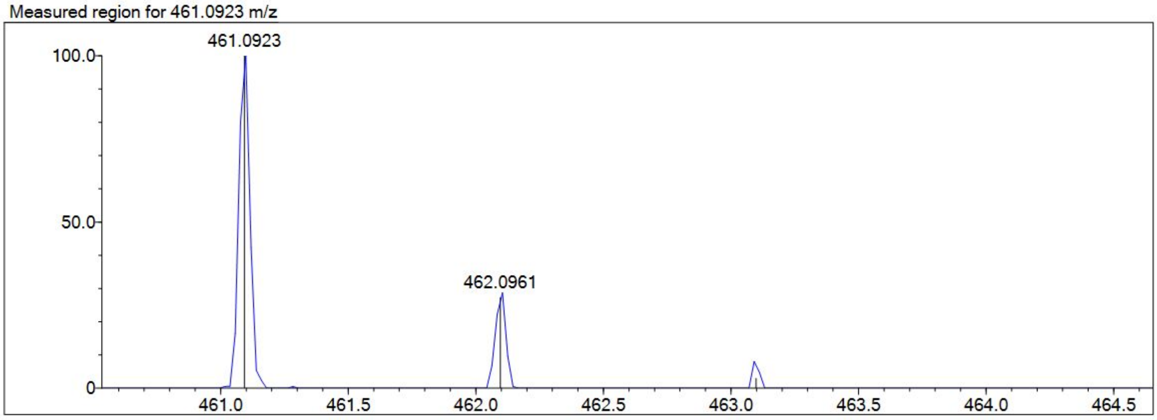

C23 H16 N4 O5 S [M+H]+ : Predicted region for 461.0914 m/z

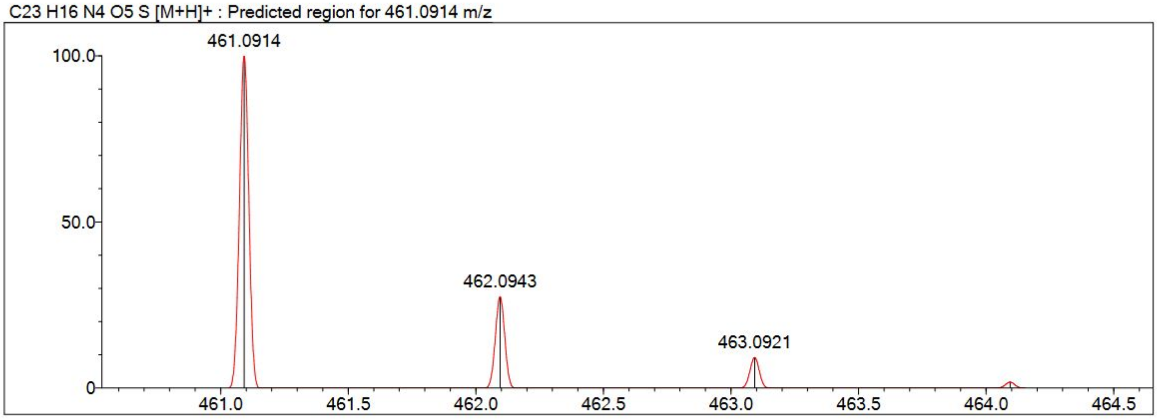

Rank	Score	Formula (M)	Ion	Meas. m/z	Pred. m/z	Df. (mDa)	Df. (ppm)	Iso	DBE
1	67.70	C23 H16 N4 O5 S	[M+H]+	461.0923	461.0914	0.9	1.95	69.35	18.0

Figure S29. Mass spectrum of compound **4i**

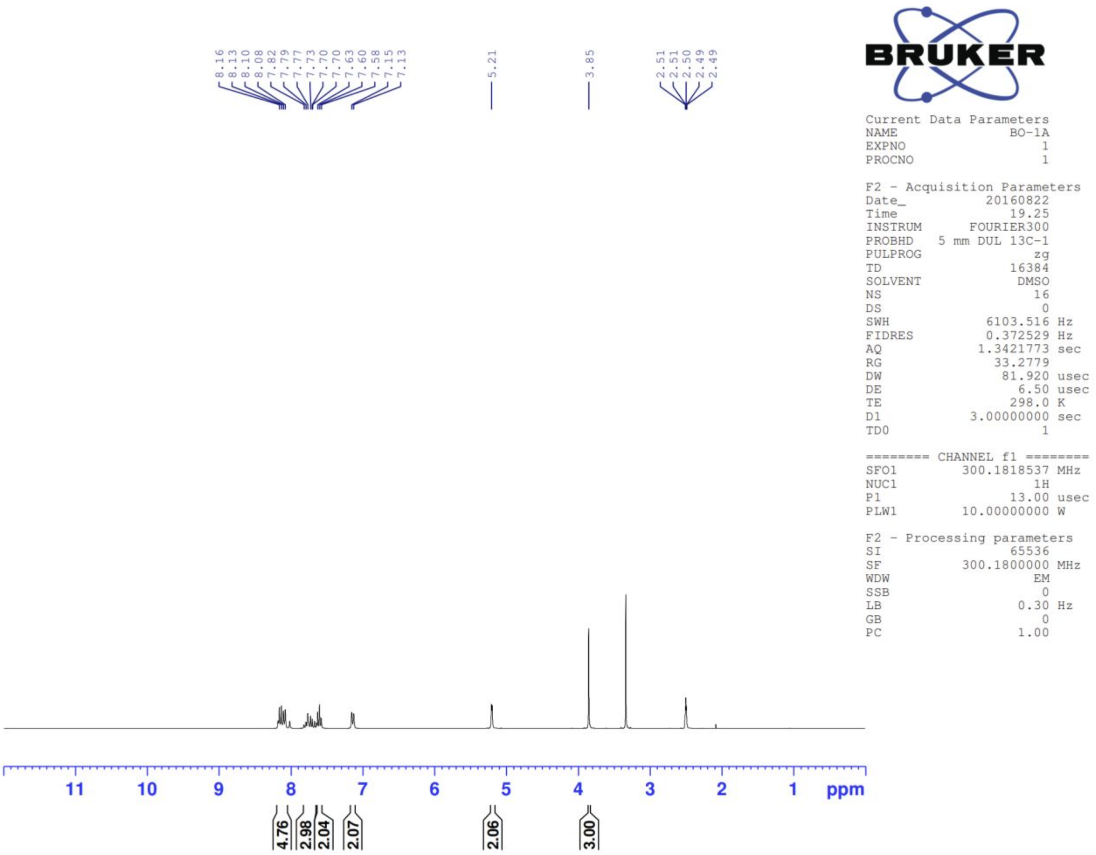

Figure S30. ^{1}H-NMR spectrum of compound **4j**

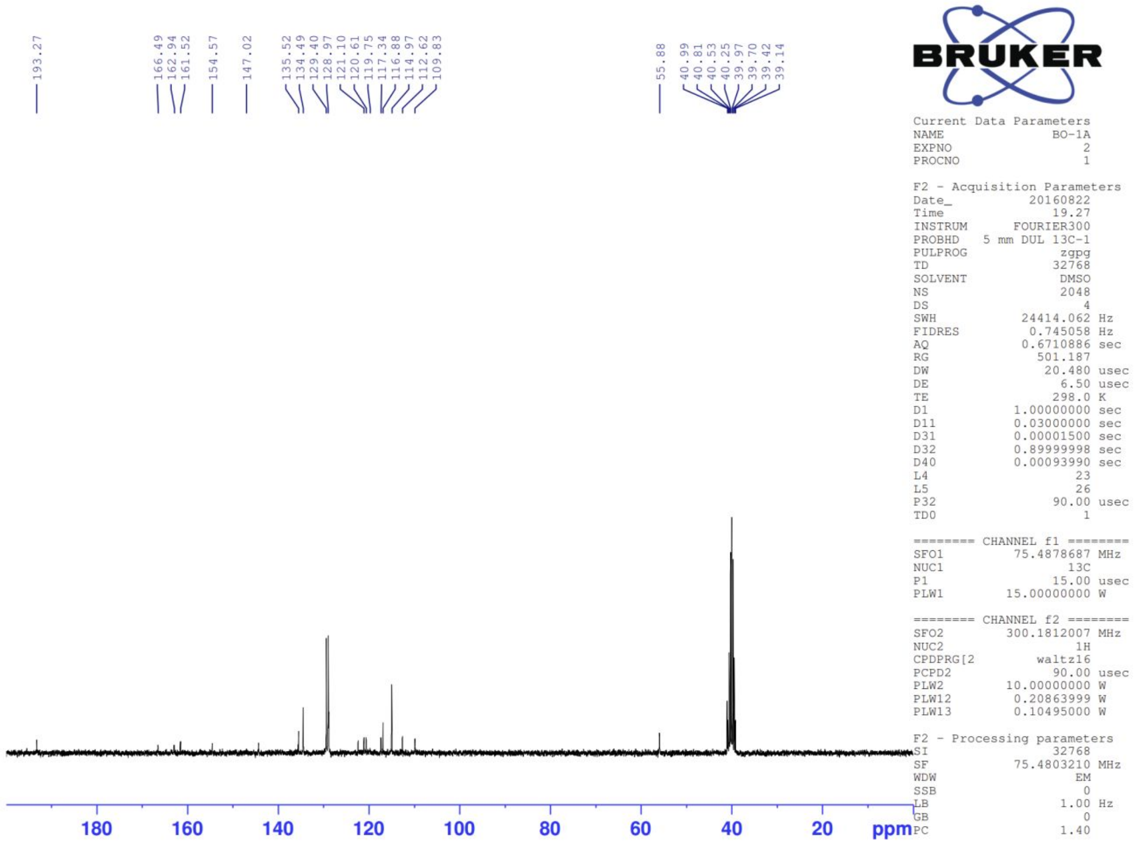

Figure S31. ^{13}C-NMR spectrum of compound **4j**

Data File: C:\LabSolutions\Data\Analiz\uac\BO1-A_2.lcd

Elmt	Val.	Min	Max	Elmt	Val.	Min	Max	Elmt	Val.	Min	Max	Use Adduct
H	1	10	25	O	2	3	5	Cl	1	0	1	H
C	4	20	29	F	1	0	1	Br	1	0	1	
N	3	4	6	S	2	1	1	I	3	0	0	

Error Margin (ppm):	10	DBE Range:	18.0 - 20.0	Electron Ions:	both
HC Ratio:	unlimited	Apply N Rule:	yes	Use MSn Info:	no
Max Isotopes:	3	Isotope RI (%):	1.00	Isotope Res:	10000
MSn Iso RI (%):	10.00	MSn Logic Mode:	AND	Max Results:	500

Event#: 1 MS(E+) Ret. Time : 6.333 -> 6.333 Scan# : 951 -> 951

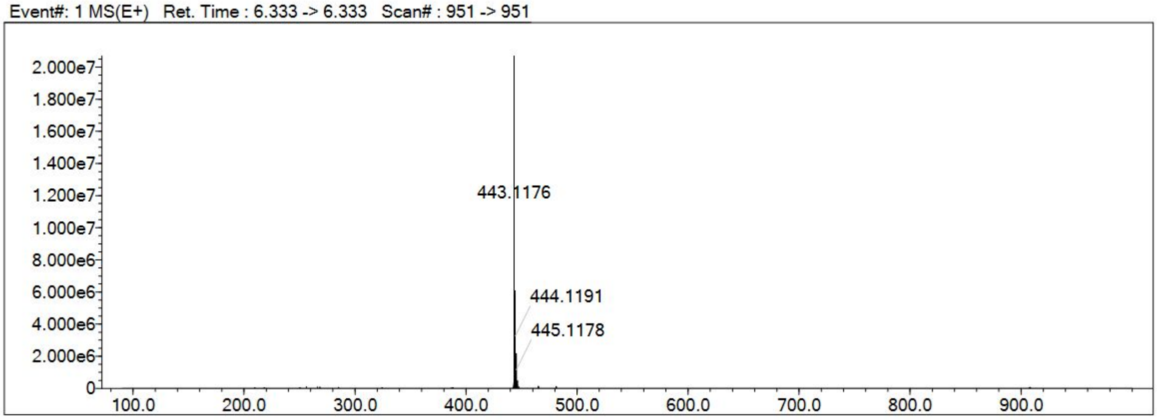

Measured region for 443.1176 m/z

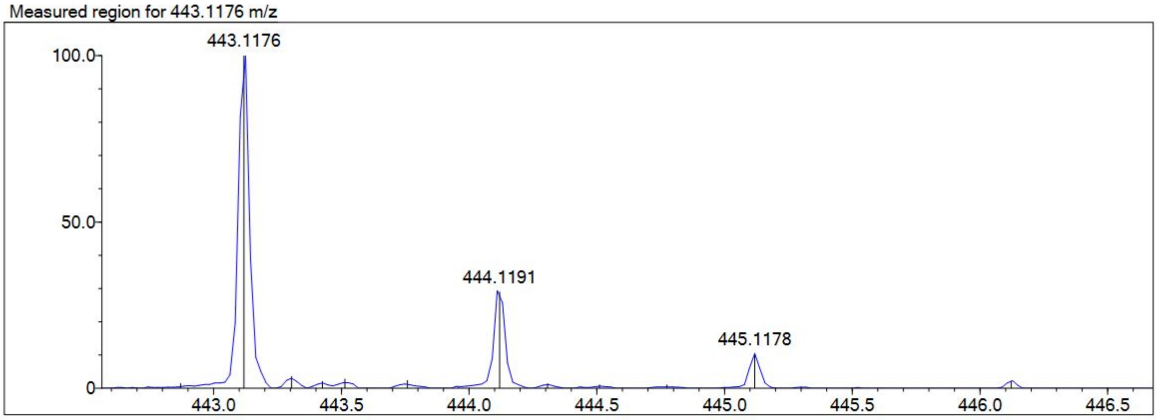

C24 H18 N4 O3 S [M+H]+ : Predicted region for 443.1172 m/z

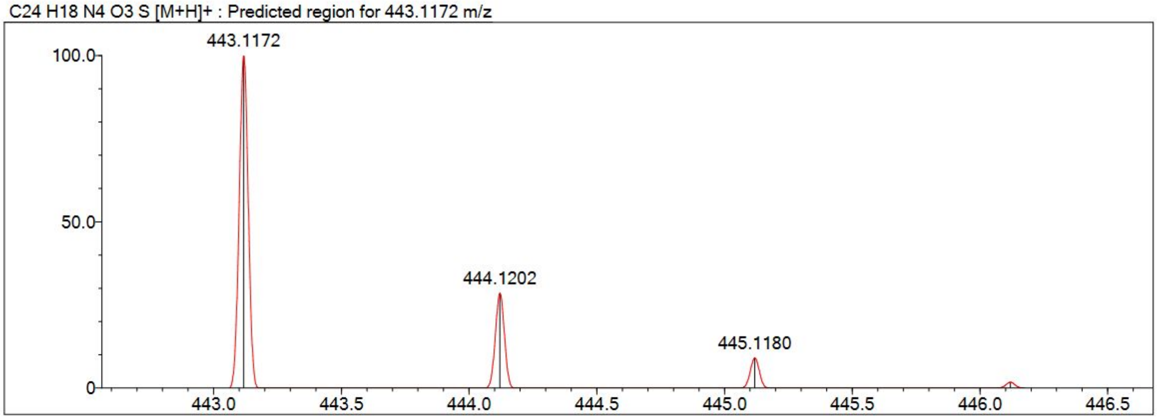

Rank	Score	Formula (M)	Ion	Meas. m/z	Pred. m/z	Df. (mDa)	Df. (ppm)	Iso	DBE
1	91.77	C24 H18 N4 O3 S	[M+H]+	443.1176	443.1172	0.4	0.90	91.77	18.0

Figure S32. Mass spectrum of compound **4j**

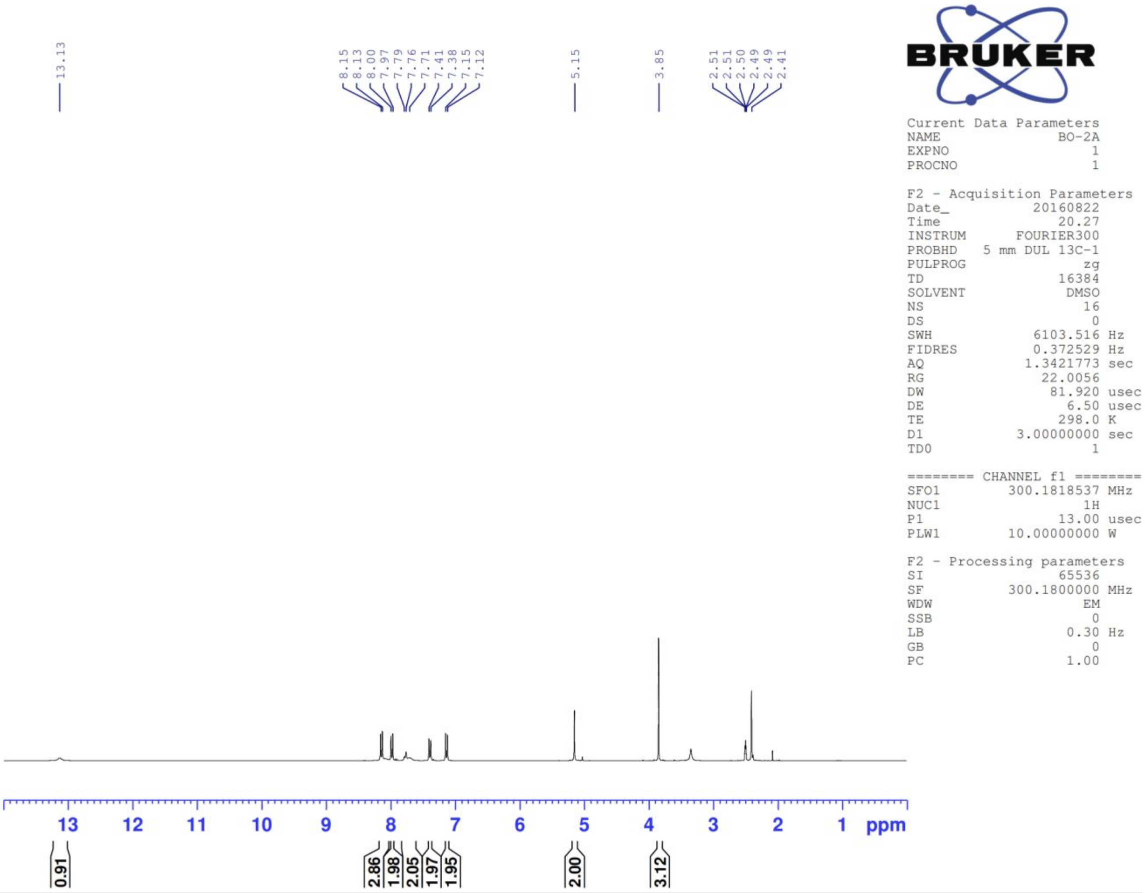

Figure S33. ^{1}H-NMR spectrum of compound **4k**

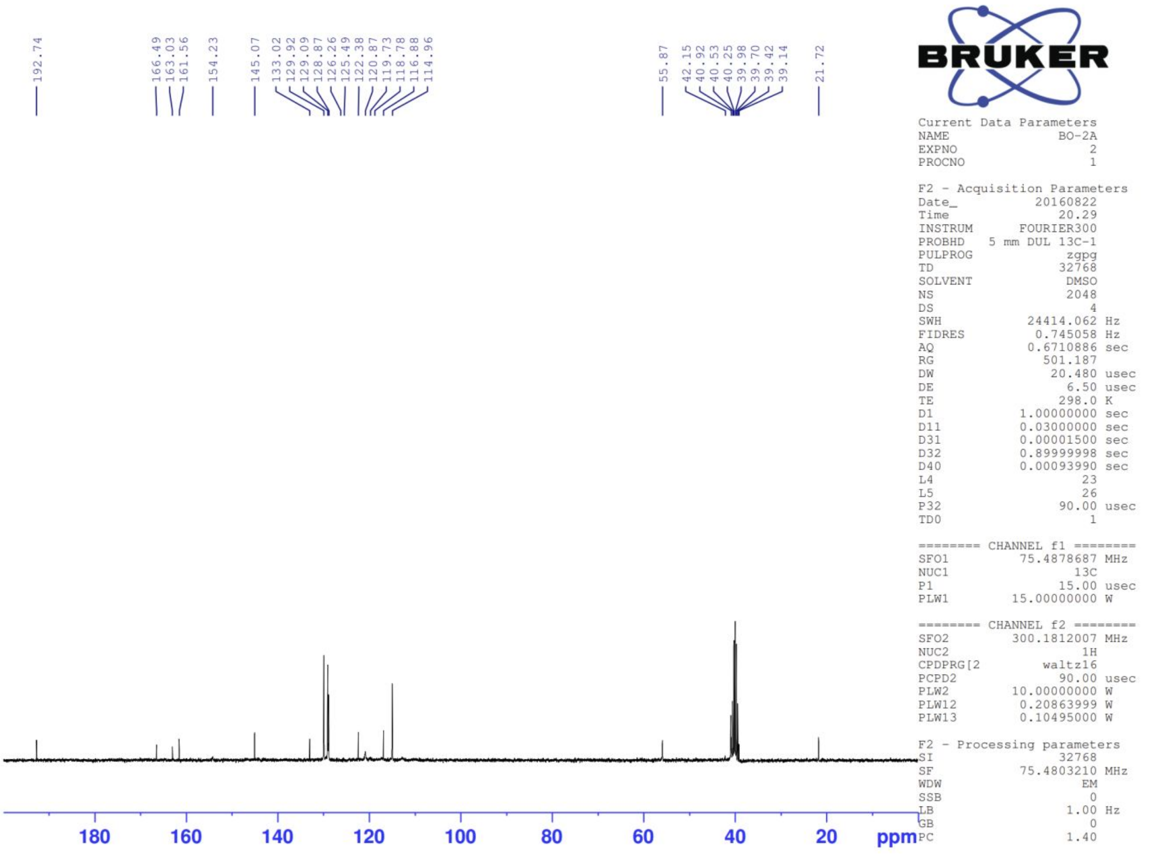

Figure S34. ^{13}C-NMR spectrum of compound **4k**

Formula Predictor Report - BO2-A_1.lcd

Data File: C:\LabSolutions\Data\Analiz\uac\BO2-A_1.lcd

Elmt	Val.	Min	Max	Elmt	Val.	Min	Max	Elmt	Val.	Min	Max	Use Adduct
H	1	10	25	O	2	3	5	Cl	1	0	1	H
C	4	20	29	F	1	0	1	Br	1	0	1	
N	3	4	6	S	2	1	1	I	3	0	0	

Error Margin (ppm):	10	DBE Range:	18.0 - 20.0	Electron Ions:	both
HC Ratio:	unlimited	Apply N Rule:	yes	Use MSn Info:	no
Max Isotopes:	3	Isotope RI (%):	1.00	Isotope Res:	10000
MSn Iso RI (%):	10.00	MSn Logic Mode:	AND	Max Results:	500

Event#: 1 MS(E+) Ret. Time : 1.680 -> 1.680 Scan# : 253 -> 253

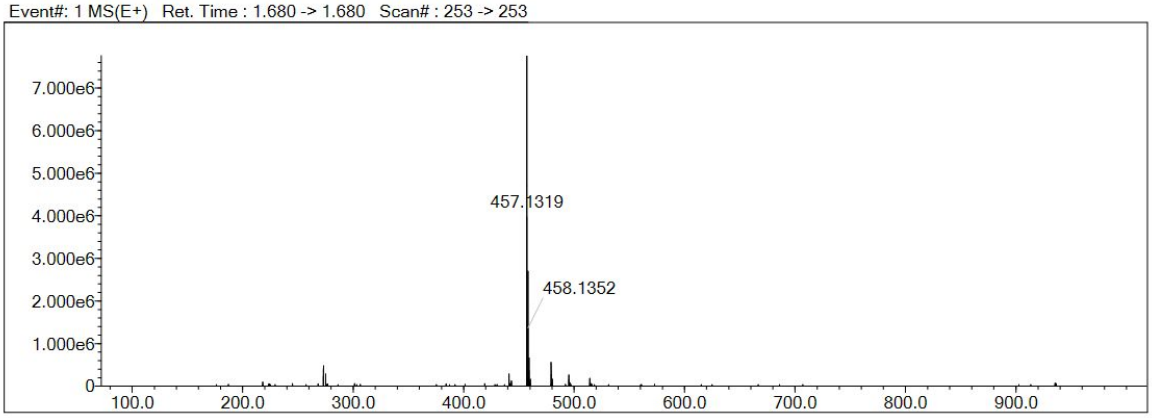

Measured region for 457.1319 m/z

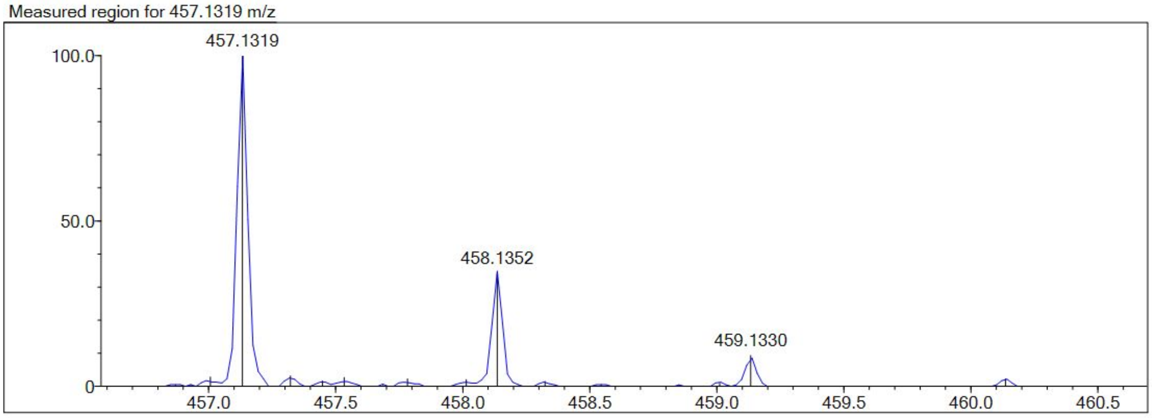

C25 H20 N4 O3 S [M+H]+ : Predicted region for 457.1329 m/z

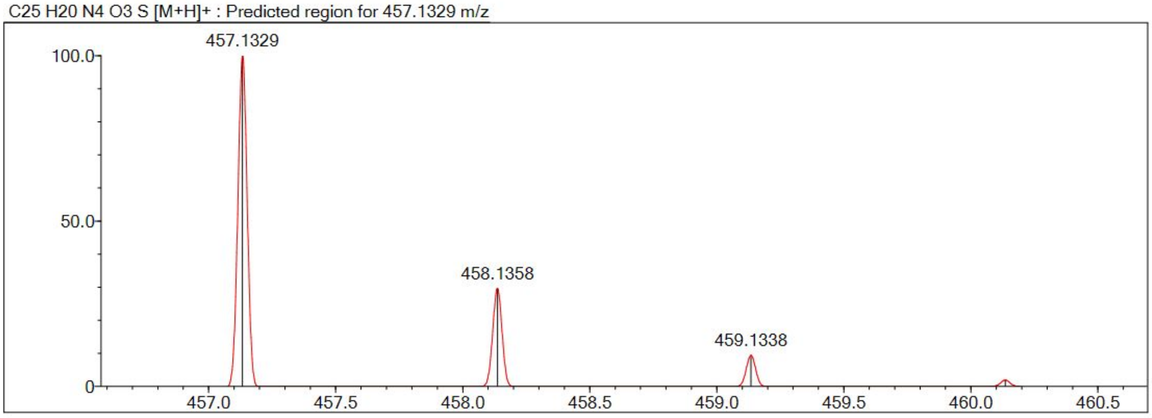

Rank	Score	Formula (M)	Ion	Meas. m/z	Pred. m/z	Df. (mDa)	Df. (ppm)	Iso	DBE
1	94.58	C25 H20 N4 O3 S	[M+H]+	457.1319	457.1329	-1.0	-2.19	97.48	18.0

Figure S35. Mass spectrum of compound **4k**

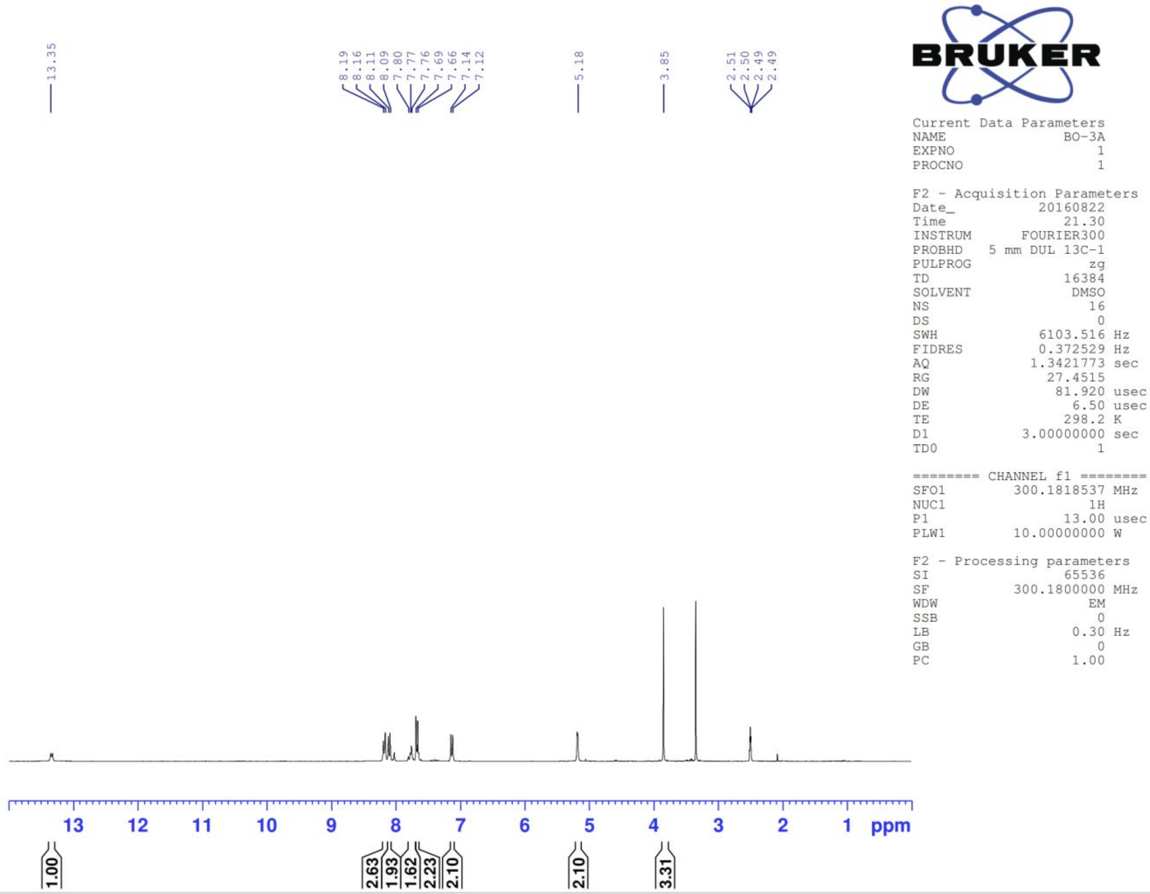

Figure S36. ^{1}H-NMR spectrum of compound **4l**

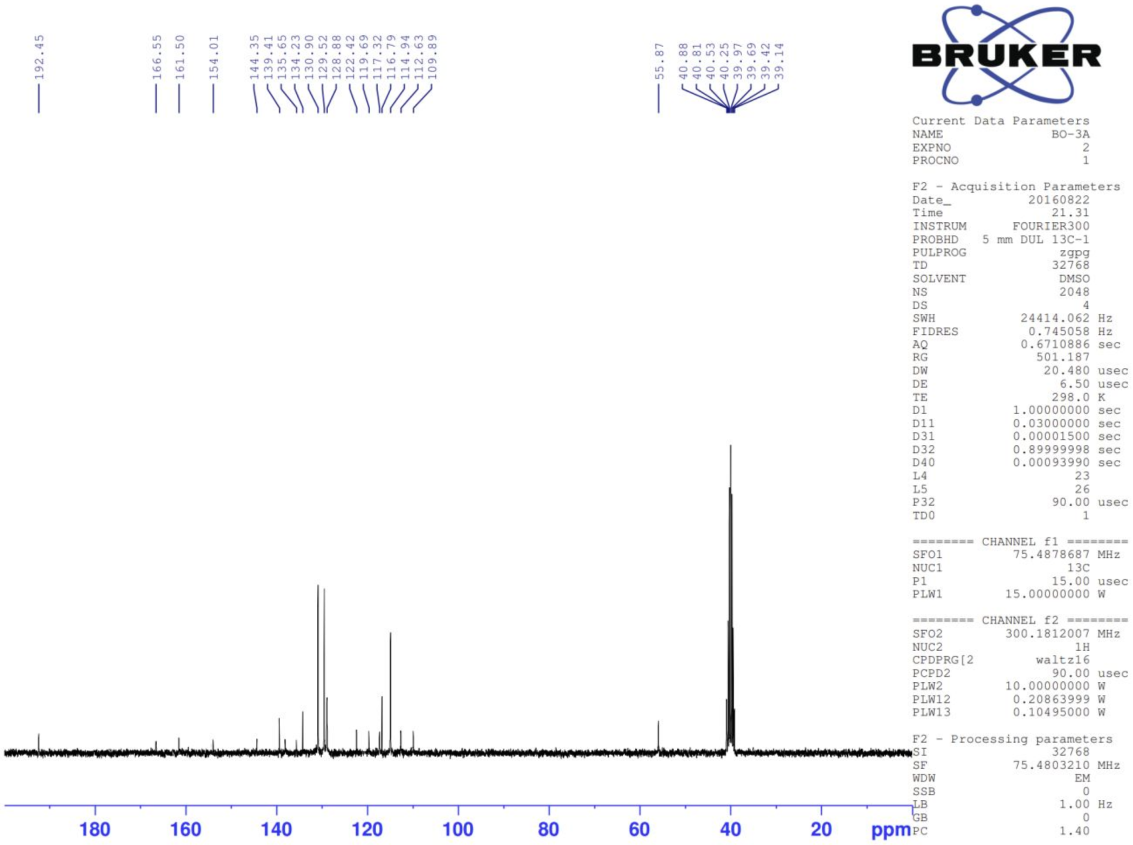

Figure S37. ^{13}C-NMR spectrum of compound **4l**

Formula Predictor Report - BO-3A_2.lcd

Data File: C:\LabSolutions\Data\Analiz\uac\BO-3A_2.lcd

Elmt	Val.	Min	Max	Elmt	Val.	Min	Max	Elmt	Val.	Min	Max	Elmt	Val.	Min	Max	Use Adduct
H	1	0	20	O	2	0	5	Cl	1	0	1	I	3	0	0	H
C	4	24	24	F	1	0	0	Br	1	0	0					
N	3	0	8	S	2	0	4	Ru	2	0	0					

Error Margin (ppm): 5
HC Ratio: unlimited
Max Isotopes: 3
MSn Iso RI (%): 10.00

DBE Range: 10.0 - 20.0
Apply N Rule: yes
Isotope RI (%): 1.00
MSn Logic Mode: AND

Electron Ions: both
Use MSn Info: no
Isotope Res: 10000
Max Results: 500

Event#: 1 MS(E+) Ret. Time : 7.320 -> 7.440 - 5.827 -> 6.192 Scan# : 1099 -> 1117 - 875 -> 929

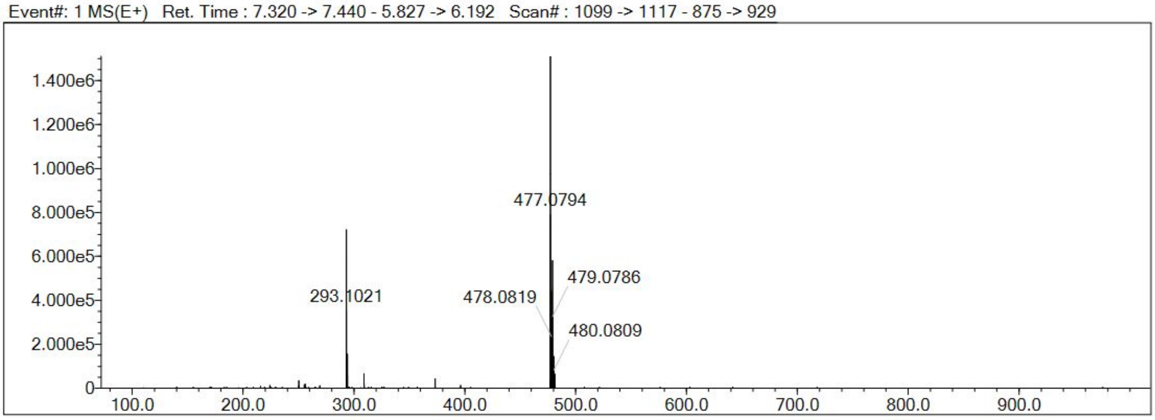

Measured region for 477.0794 m/z

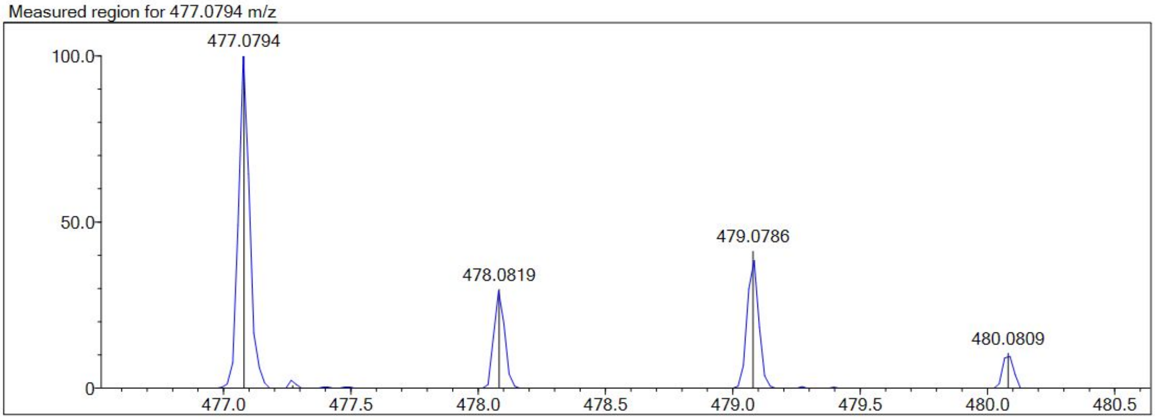

C24 H17 N4 O3 S Cl [M+H]+ : Predicted region for 477.0783 m/z

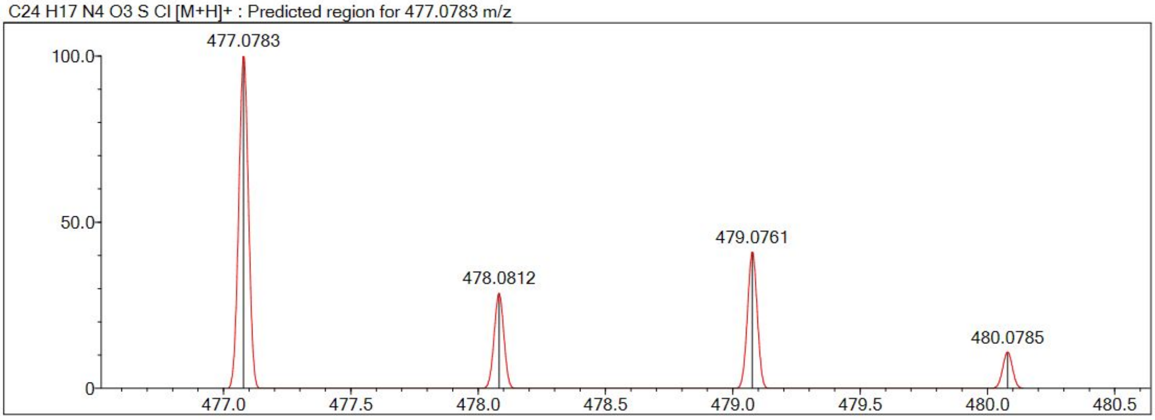

Rank	Score	Formula (M)	Ion	Meas. m/z	Pred. m/z	Df. (mDa)	Df. (ppm)	Iso	DBE
1	87.23	C24 H17 N4 O3 S Cl	[M+H]+	477.0794	477.0783	1.1	2.31	90.19	18.0

Figure S38. Mass spectrum of compound **4l**

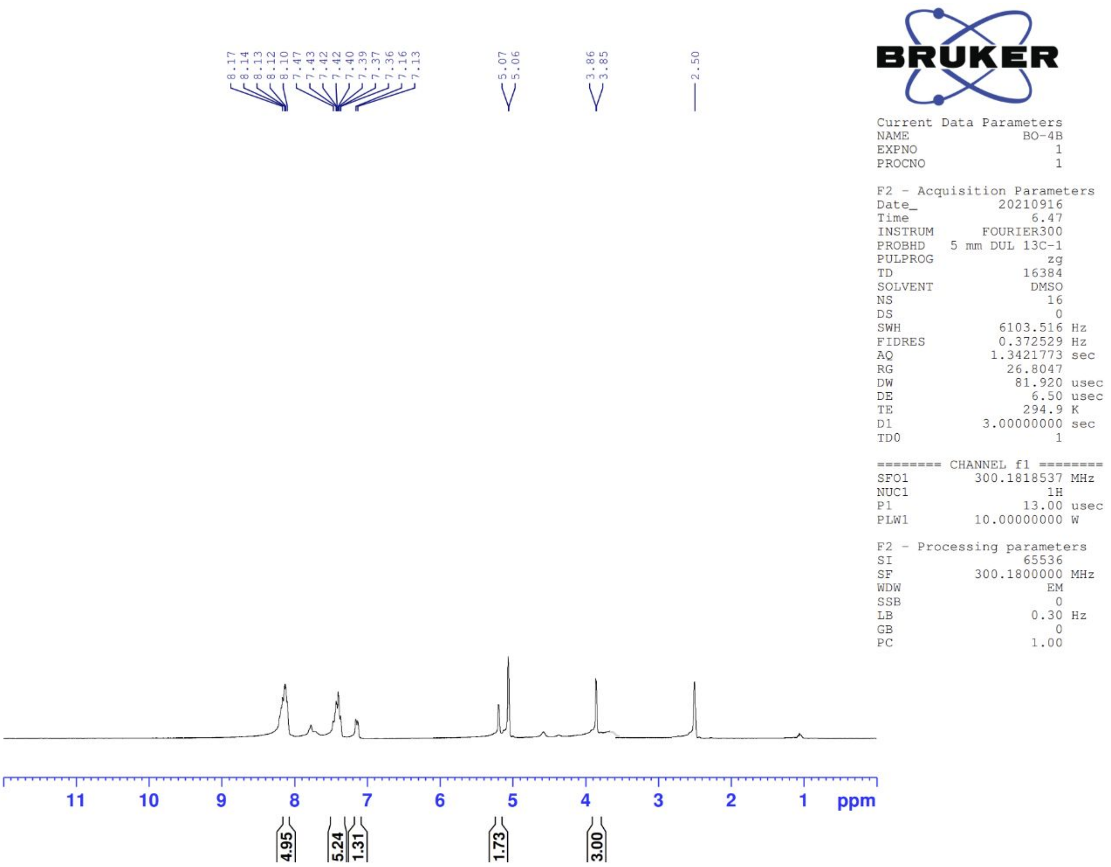

Figure S39. ^{1}H-NMR spectrum of compound **4m**

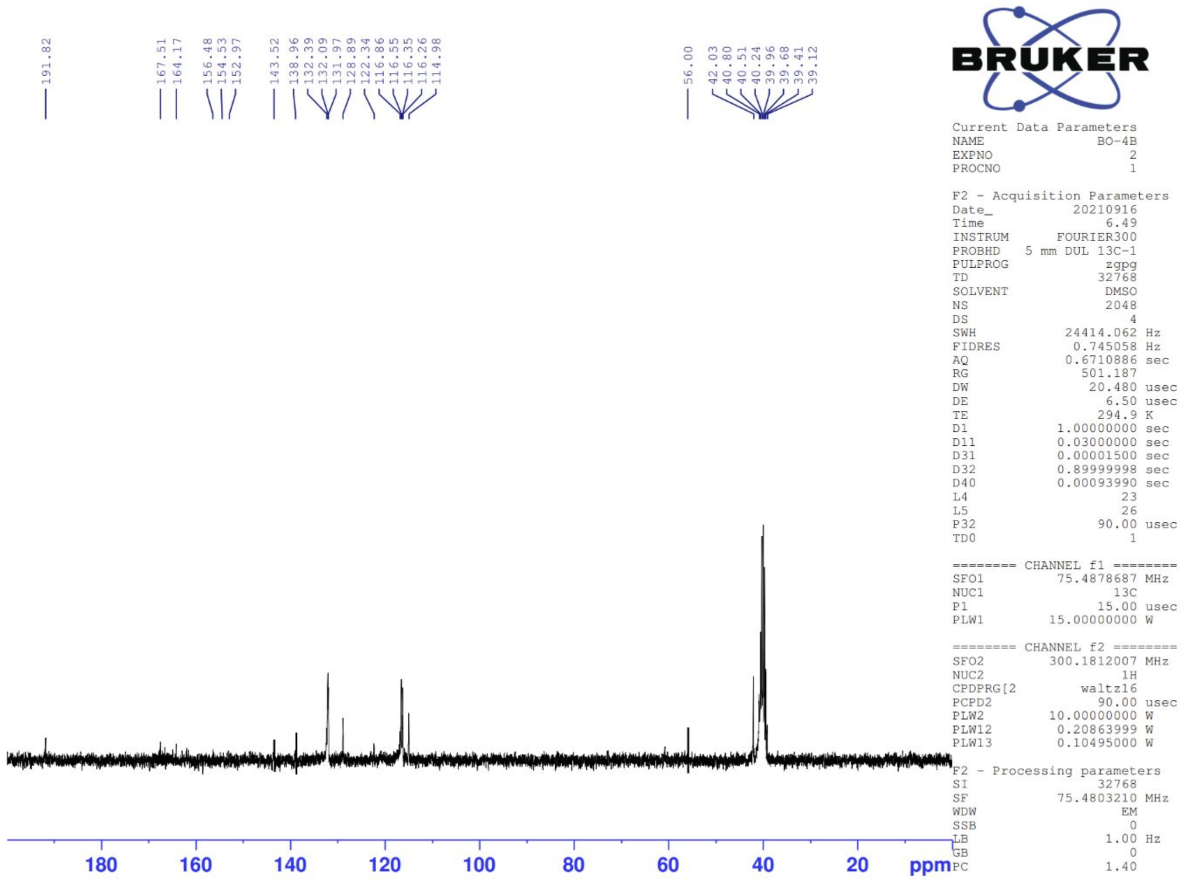

Figure S40. ^{13}C-NMR spectrum of compound **4m**

Formula Predictor Report - BO-4A_5.lcd Page 1 of 1

Data File: C:\LabSolutions\Data\Analiz\uac\BO-4A_5.lcd

Elmt	Val.	Min	Max	Elmt	Val.	Min	Max	Elmt	Val.	Min	Max	Elmt	Val.	Min	Max	Use Adduct
H	1	0	17	O	2	0	5	Cl	1	0	0	I	3	0	0	H
C	4	24	40	F	1	0	2	Br	1	0	0					
N	3	0	8	S	2	0	4	Ru	2	0	0					

Error Margin (ppm):	5	DBE Range:	10.0 - 20.0	Electron Ions:	both
HC Ratio:	unlimited	Apply N Rule:	yes	Use MSn Info:	no
Max Isotopes:	3	Isotope RI (%):	1.00	Isotope Res:	10000
MSn Iso RI (%):	10.00	MSn Logic Mode:	AND	Max Results:	500

Event#: 1 MS(E+) Ret. Time : 6.640 -> 7.027 - 8.040 -> 9.352 Scan# : 997 -> 1055 - 1207 -> 1403

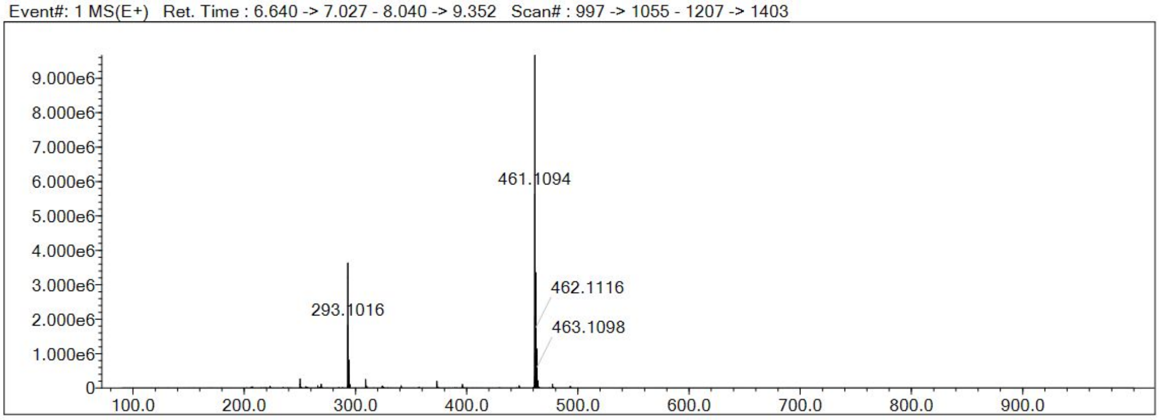

Measured region for 461.1094 m/z

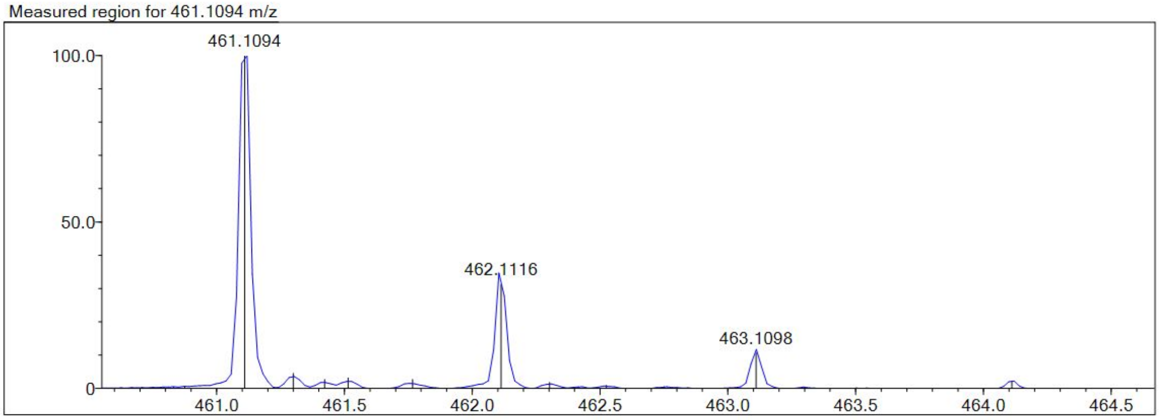

C24 H17 N4 O3 F S [M+H]+ : Predicted region for 461.1078 m/z

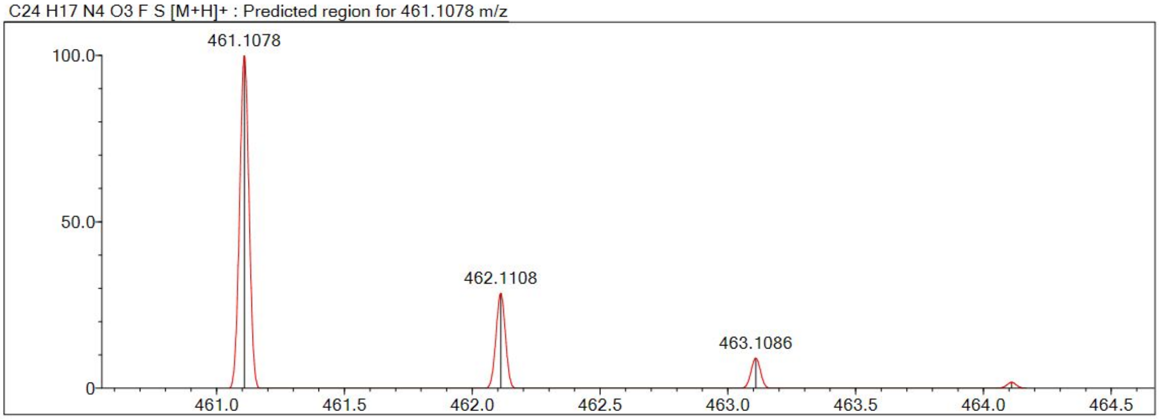

Rank	Score	Formula (M)	Ion	Meas. m/z	Pred. m/z	Df. (mDa)	Df. (ppm)	Iso	DBE
1	92.24	C24 H17 N4 O3 F S	[M+H]+	461.1094	461.1078	1.6	3.47	98.31	18.0

Figure S41. Mass spectrum of compound **4m**

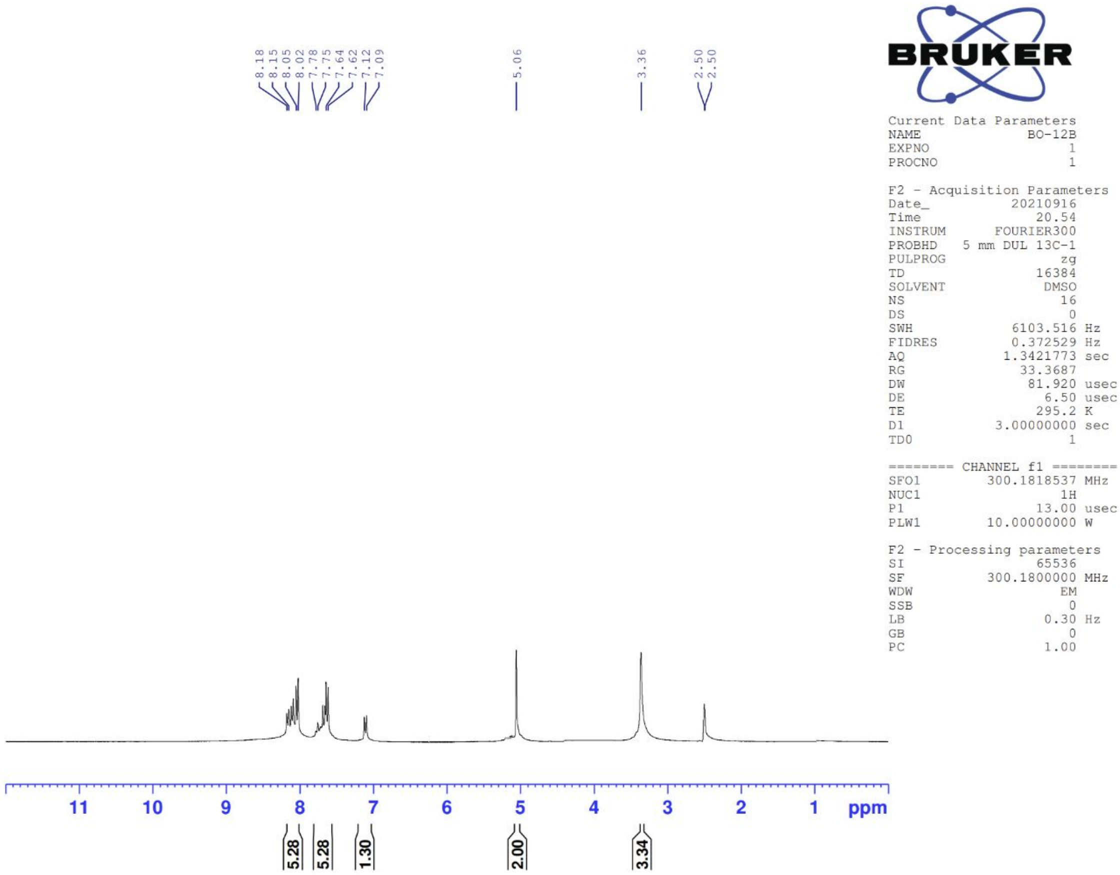

Figure S42. ^{1}H-NMR spectrum of compound **4n**

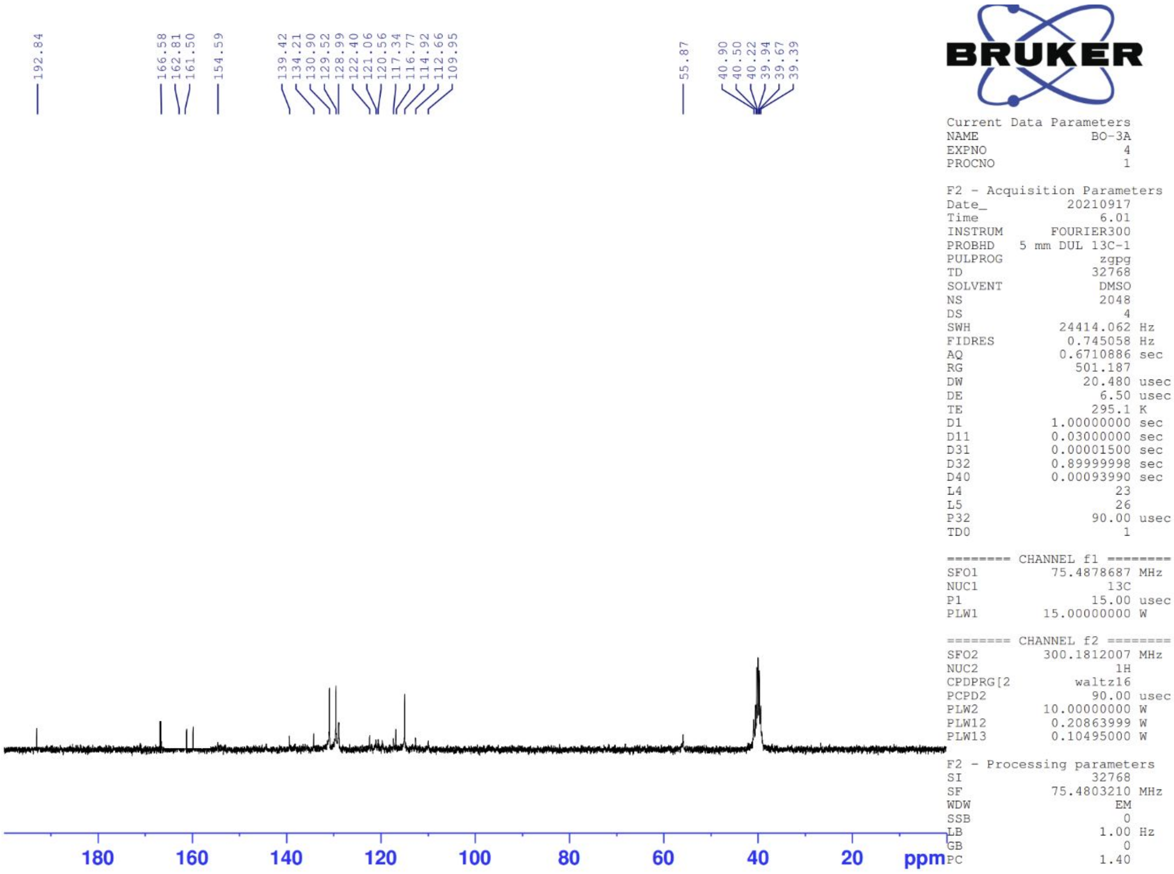

Figure S43. ^{13}C-NMR spectrum of compound **4n**

Formula Predictor Report - BO5_8.lcd Page 1 of 1

Data File: C:\LabSolutions\Data\Analiz\uac\BO5_8.lcd

Elmt	Val.	Min	Max	Elmt	Val.	Min	Max	Elmt	Val.	Min	Max	Use Adduct
H	1	10	25	O	2	3	5	Cl	1	0	2	H
C	4	20	29	F	1	0	2	Br	1	0	1	
N	3	4	5	S	2	1	1	I	3	0	0	

Error Margin (ppm): 5
HC Ratio: unlimited
Max Isotopes: 3
MSn Iso RI (%): 10.00

DBE Range: 18.0 - 20.0
Apply N Rule: yes
Isotope RI (%): 1.00
MSn Logic Mode: AND

Electron Ions: both
Use MSn Info: no
Isotope Res: 10000
Max Results: 500

Event#: 1 MS(E+) Ret. Time : 7.080 -> 7.080 Scan# : 1063 -> 1063

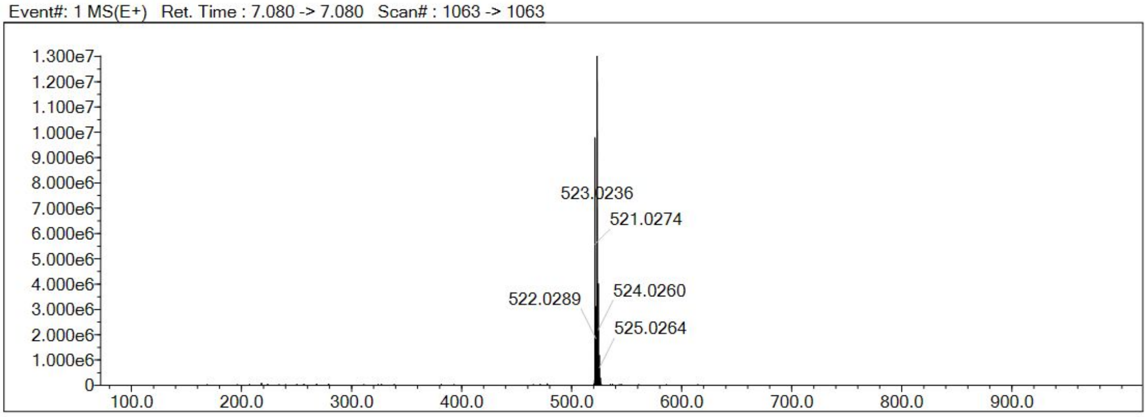

Measured region for 521.0274 m/z

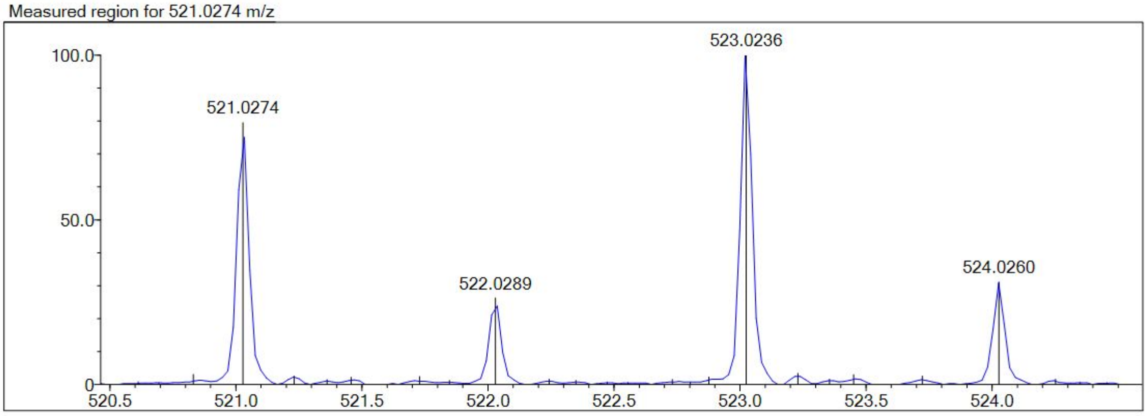

C24 H17 N4 O3 S Br [M+H]+ : Predicted region for 521.0277 m/z

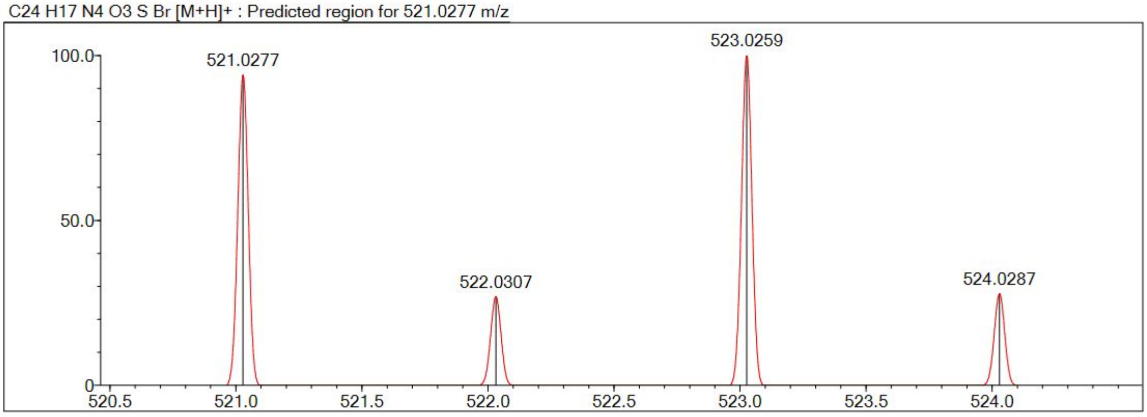

Rank	Score	Formula (M)	Ion	Meas. m/z	Pred. m/z	Df. (mDa)	Df. (ppm)	Iso	DBE
1	61.66	C24 H17 N4 O3 S Br	[M+H]+	521.0274	521.0277	-0.3	-0.58	61.66	18.0

Figure S44. Mass spectrum of compound **4n**

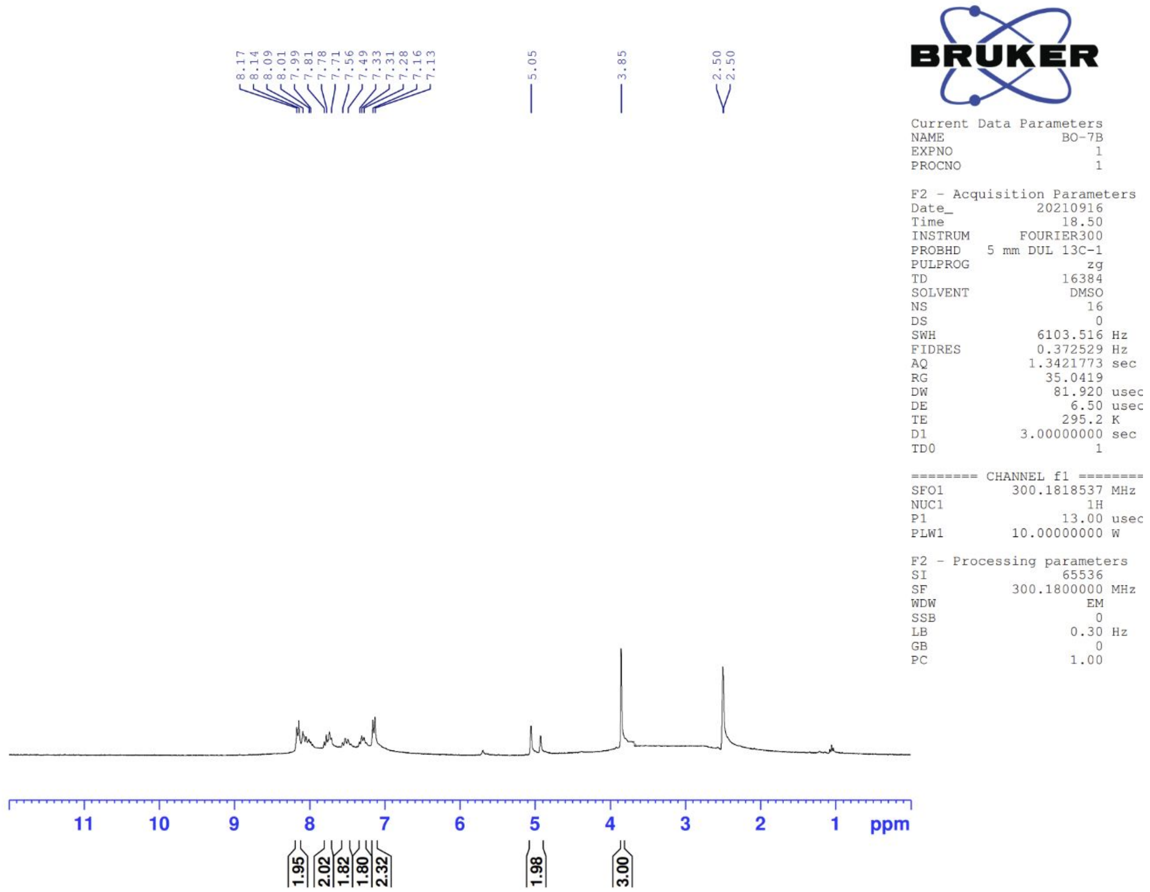

Figure S45. ^{1}H-NMR spectrum of compound **4o**

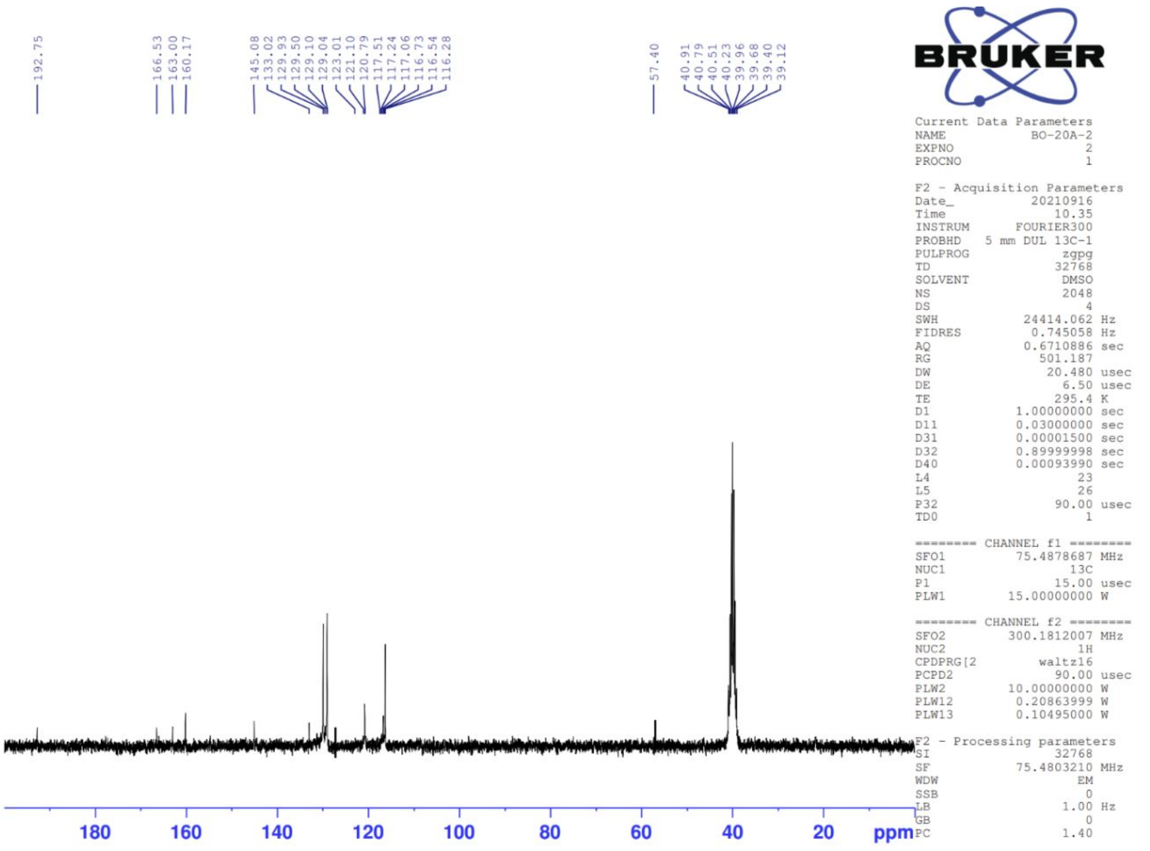

Figure S46. ^{13}C-NMR spectrum of compound **4o**

Data File: C:\LabSolutions\Data\Analiz\uac\BO-6A_7.lcd

Elmt	Val.	Min	Max	Elmt	Val.	Min	Max	Elmt	Val.	Min	Max	Elmt	Val.	Min	Max	Use Adduct
H	1	0	17	O	2	0	5	Cl	1	0	0	I	3	0	0	H
C	4	24	40	F	1	0	2	Br	1	0	0					
N	3	0	8	S	2	0	4	Ru	2	0	0					

Error Margin (ppm):	5	DBE Range:	10.0 - 20.0	Electron Ions:	both
HC Ratio:	unlimited	Apply N Rule:	yes	Use MSn Info:	no
Max Isotopes:	3	Isotope RI (%):	1.00	Isotope Res:	10000
MSn Iso RI (%):	10.00	MSn Logic Mode:	AND	Max Results:	500

Event#: 1 MS(E+) Ret. Time : 6.587 -> 6.667 - 7.800 -> 9.388 Scan# : 989 -> 1001 - 1171 -> 1409

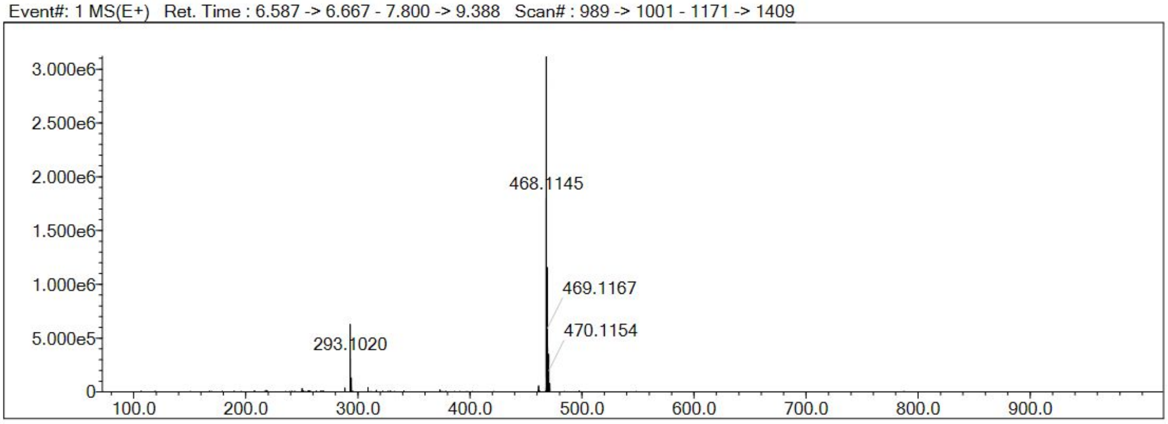

Measured region for 468.1145 m/z

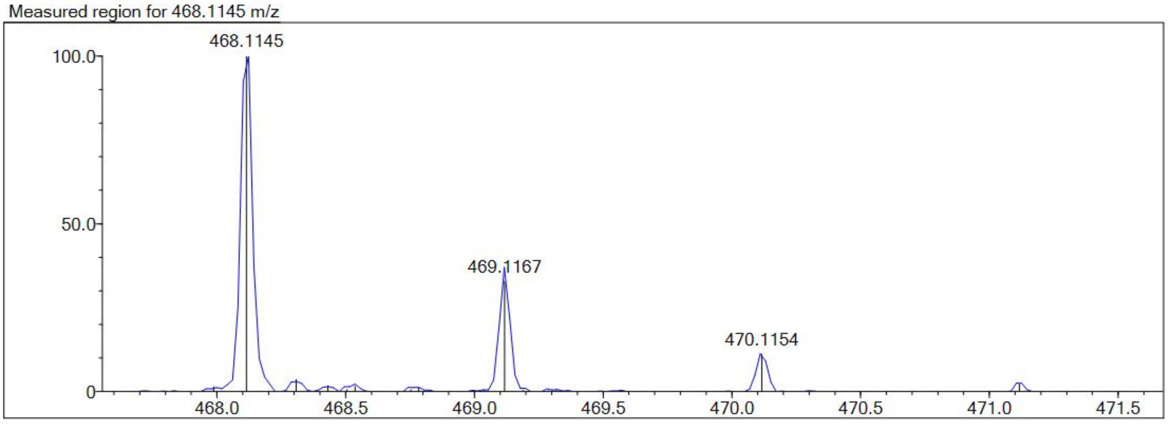

C25 H17 N5 O3 S [M+H]+ : Predicted region for 468.1125 m/z

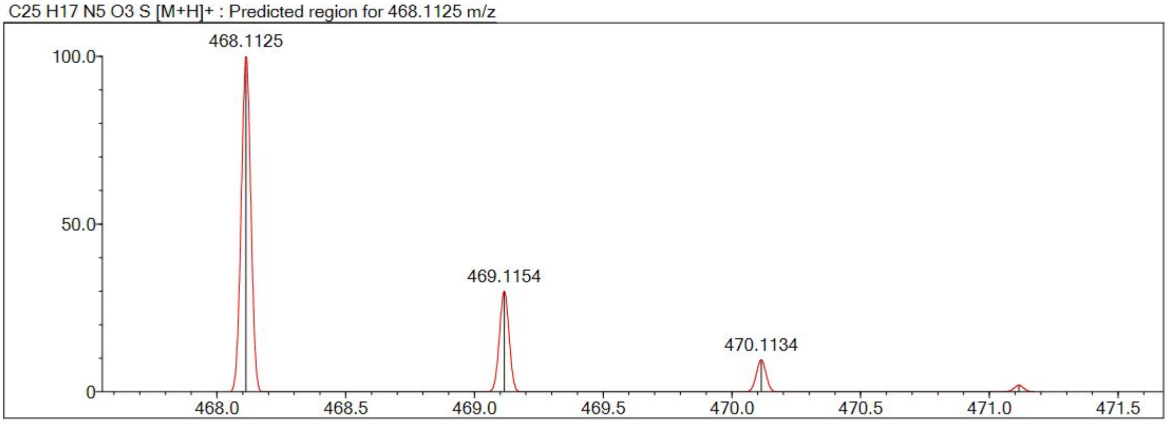

Rank	Score	Formula (M)	Ion	Meas. m/z	Pred. m/z	Df. (mDa)	Df. (ppm)	Iso	DBE
1	78.45	C25 H17 N5 O3 S	[M+H]+	468.1145	468.1125	2.0	4.27	85.43	20.0

Figure S47. Mass spectrum of compound **4o**

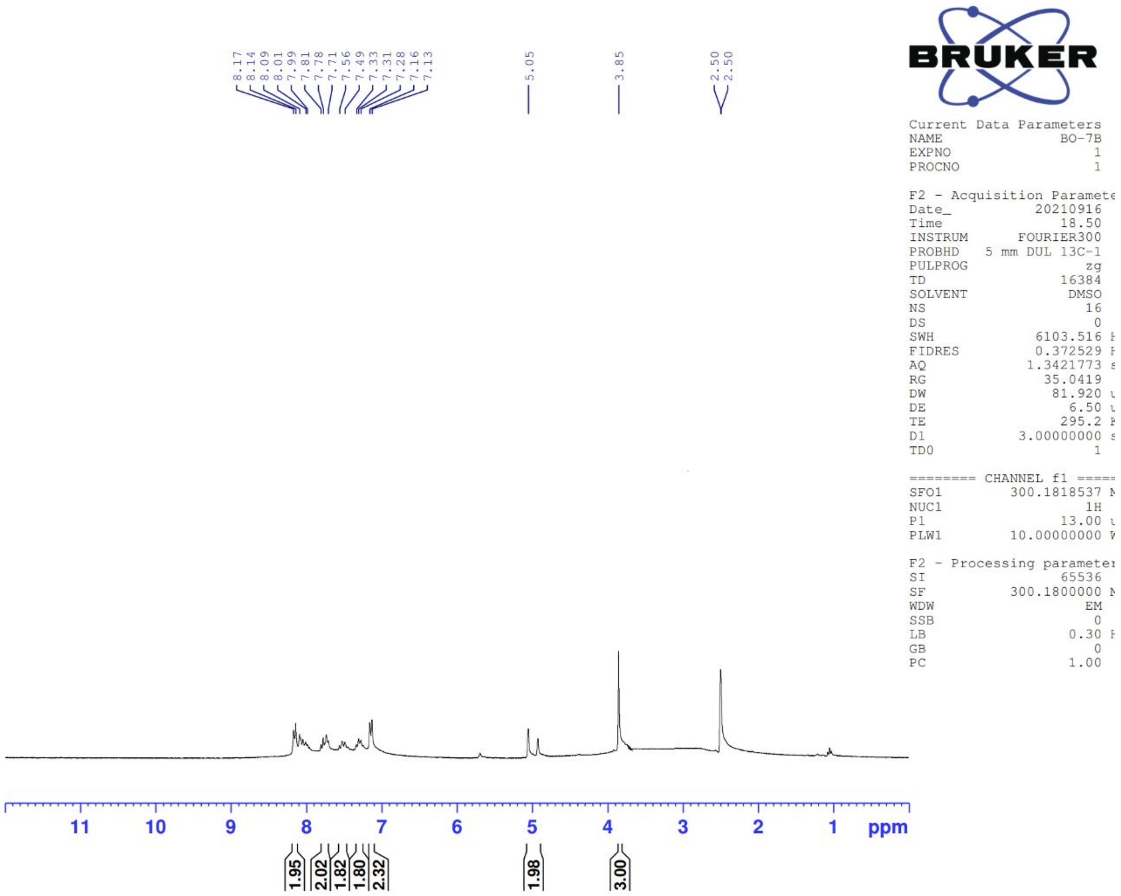

Figure S48. ^{1}H-NMR spectrum of compound **4p**

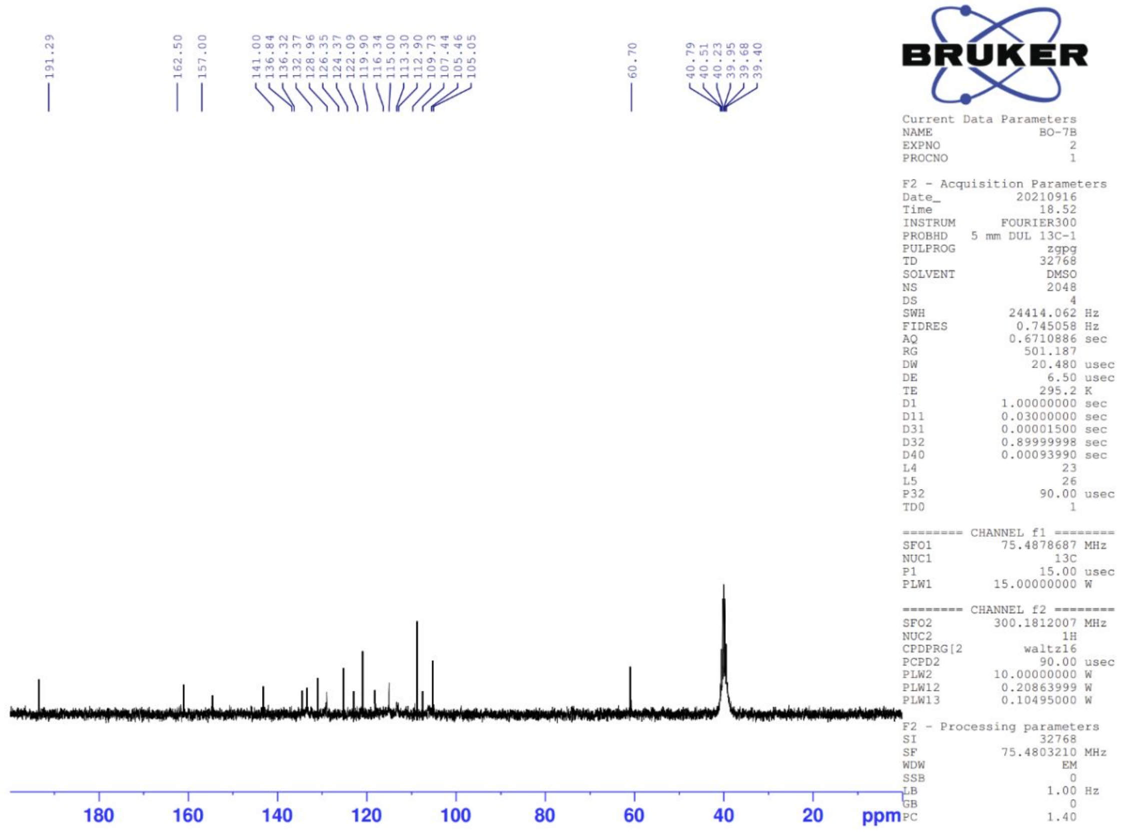

Figure S49. ^{13}C-NMR spectrum of compound **4p**

Data File: C:\LabSolutions\Data\Analiz\uac\BO7-A_2.lcd

Elmt	Val.	Min	Max	Elmt	Val.	Min	Max	Elmt	Val.	Min	Max	Use Adduct
H	1	10	25	O	2	3	5	Cl	1	0	1	H
C	4	20	29	F	1	0	2	Br	1	0	1	
N	3	4	6	S	2	1	1	I	3	0	0	

Error Margin (ppm):	5	DBE Range:	18.0 - 20.0	Electron Ions:	both
HC Ratio:	unlimited	Apply N Rule:	yes	Use MSn Info:	no
Max Isotopes:	3	Isotope RI (%):	1.00	Isotope Res:	10000
MSn Iso RI (%):	10.00	MSn Logic Mode:	AND	Max Results:	500

Event#: 1 MS(E+) Ret. Time : 6.507 -> 6.600 Scan# : 977 -> 991

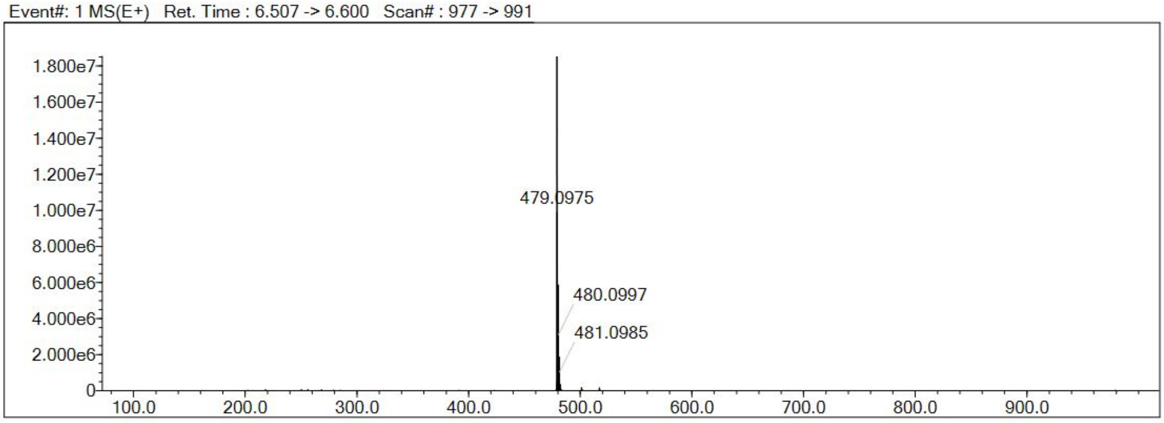

Measured region for 479.0975 m/z

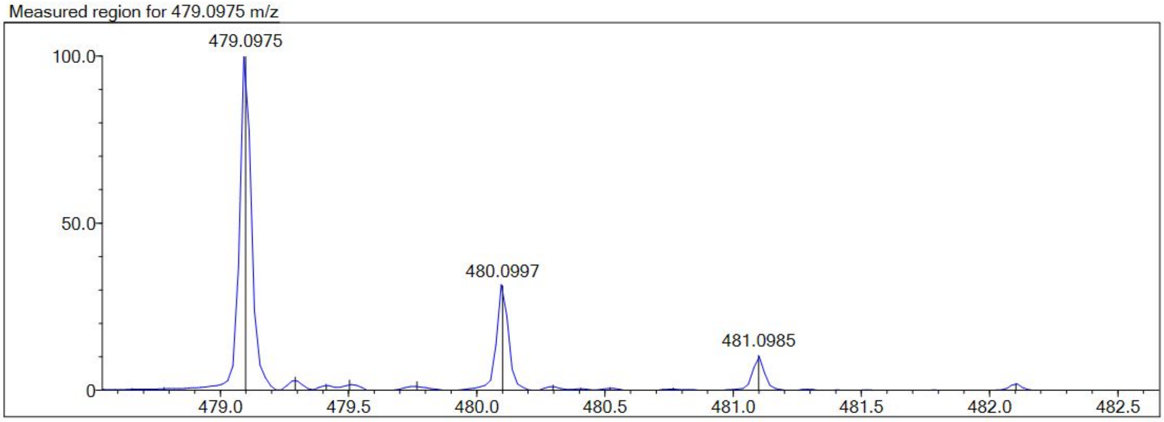

C24 H16 N4 O3 F2 S [M+H]+ : Predicted region for 479.0984 m/z

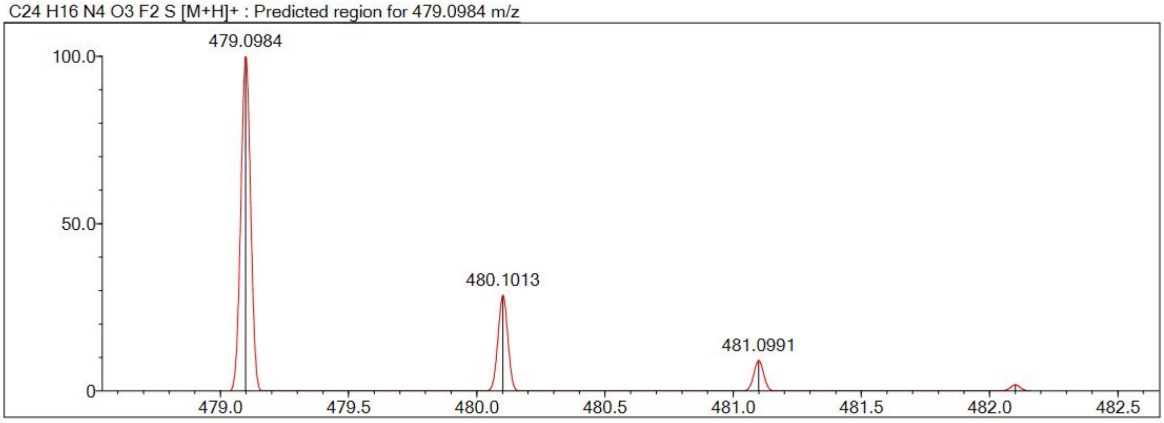

Rank	Score	Formula (M)	Ion	Meas. m/z	Pred. m/z	Df. (mDa)	Df. (ppm)	Iso	DBE
1	83.12	C24 H16 N4 O3 F2 S	[M+H]+	479.0975	479.0984	-0.9	-1.88	84.99	18.0

Figure S50. Mass spectrum of compound **4p**

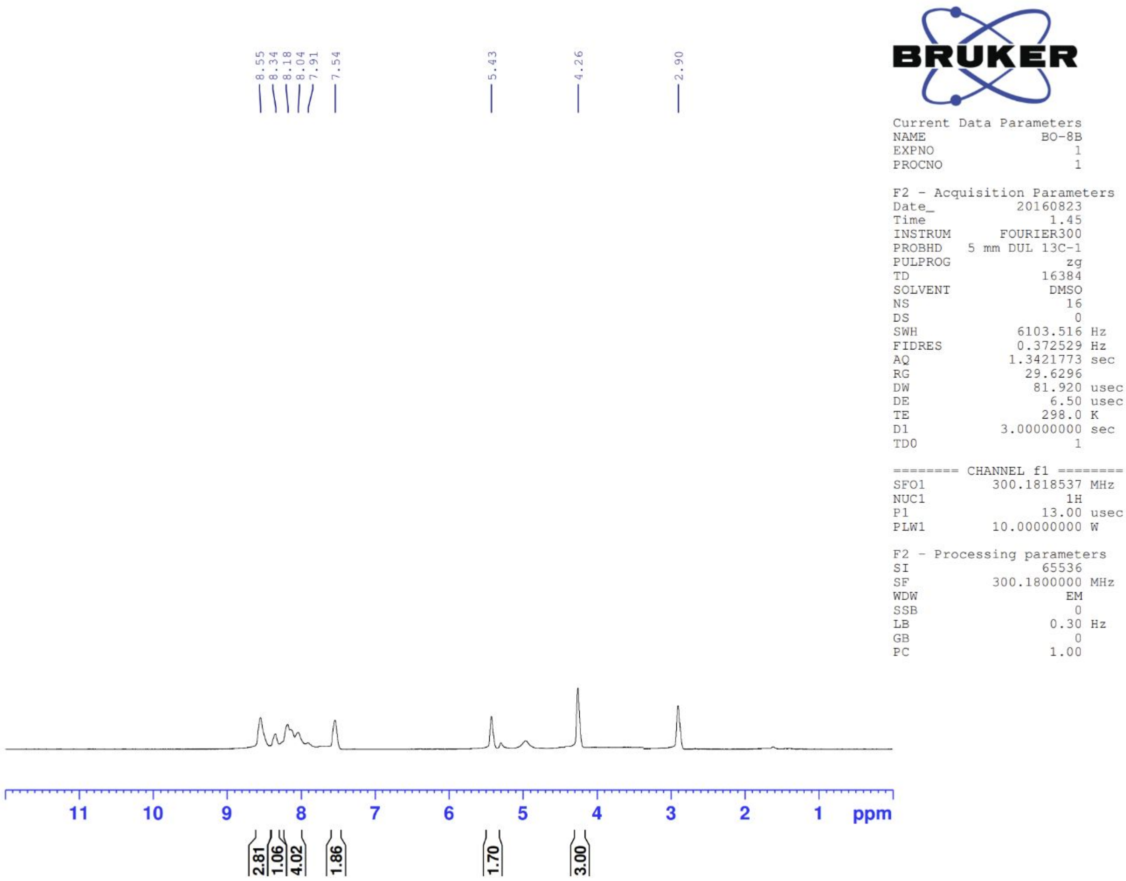

Figure S51. ^{1}H-NMR spectrum of compound **4r**

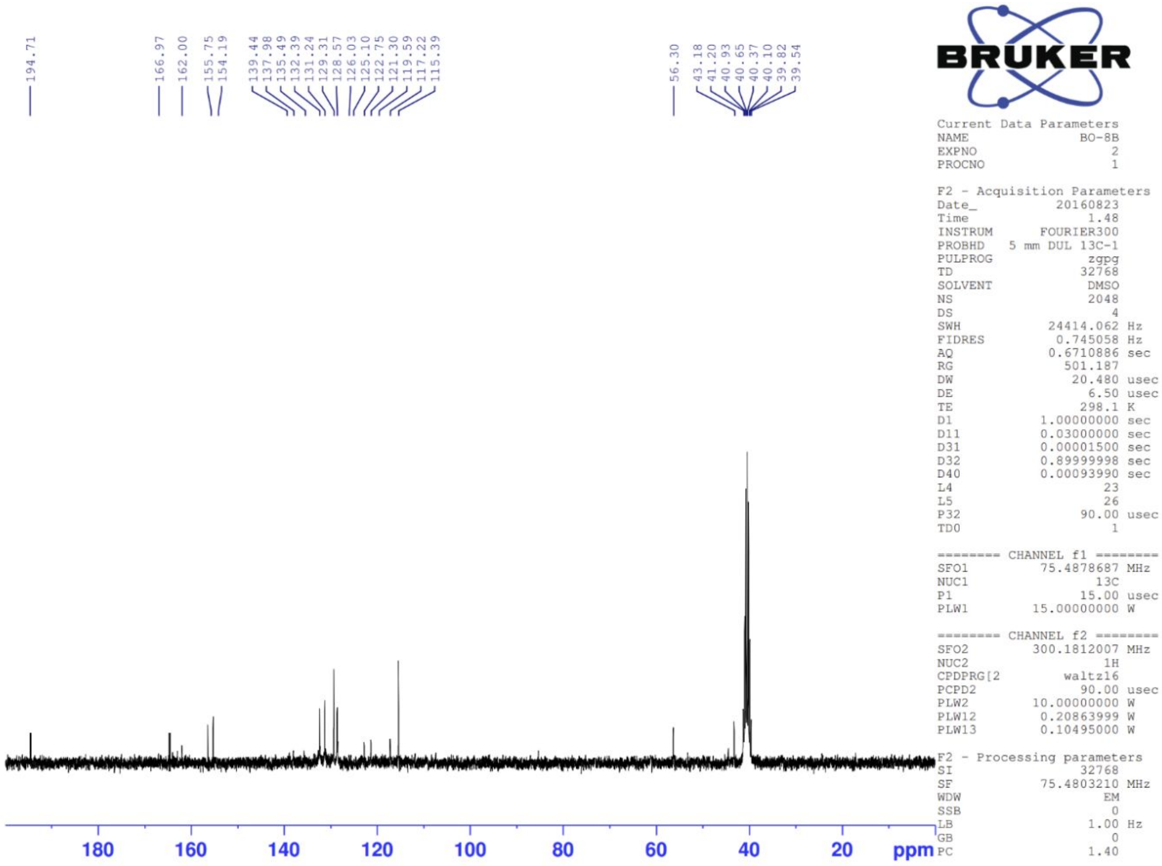

Figure S52. ^{13}C-NMR spectrum of compound **4r**

Formula Predictor Report - BO8-B_5.lcd

Page 1 of 1

Data File: C:\LabSolutions\Data\Analiz\uac\BO8-B_5.lcd

Elmt	Val.	Min	Max	Elmt	Val.	Min	Max	Elmt	Val.	Min	Max	Use Adduct
H	1	10	25	O	2	3	5	Cl	1	0	2	H
C	4	20	29	F	1	0	2	Br	1	0	1	
N	3	4	5	S	2	1	1	I	3	0	0	

Error Margin (ppm): 5
HC Ratio: unlimited
Max Isotopes: 3
MSn Iso RI (%): 10.00

DBE Range: 18.0 - 20.0
Apply N Rule: yes
Isotope RI (%): 1.00
MSn Logic Mode: AND

Electron Ions: both
Use MSn Info: no
Isotope Res: 10000
Max Results: 500

Event#: 1 MS(E+) Ret. Time : 7.107 -> 7.107 Scan# : 1067 -> 1067

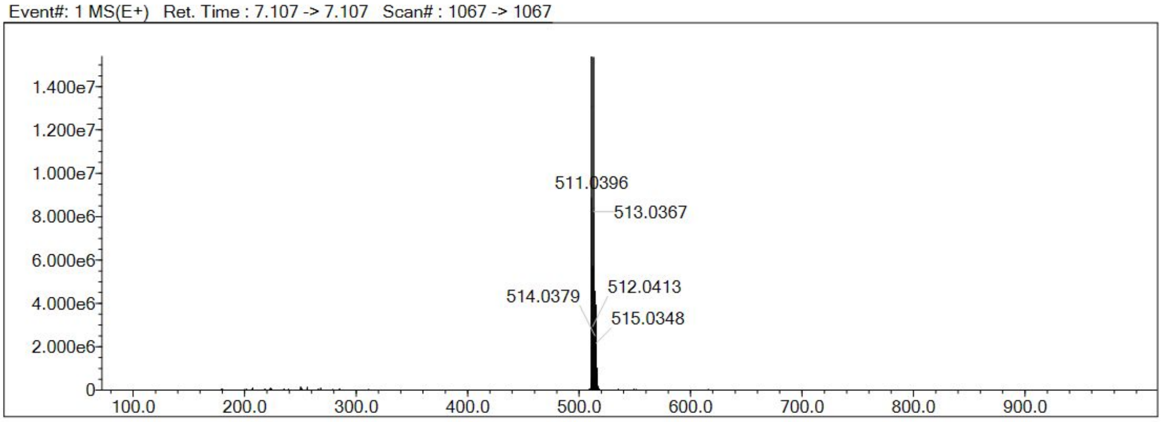

Measured region for 511.0396 m/z

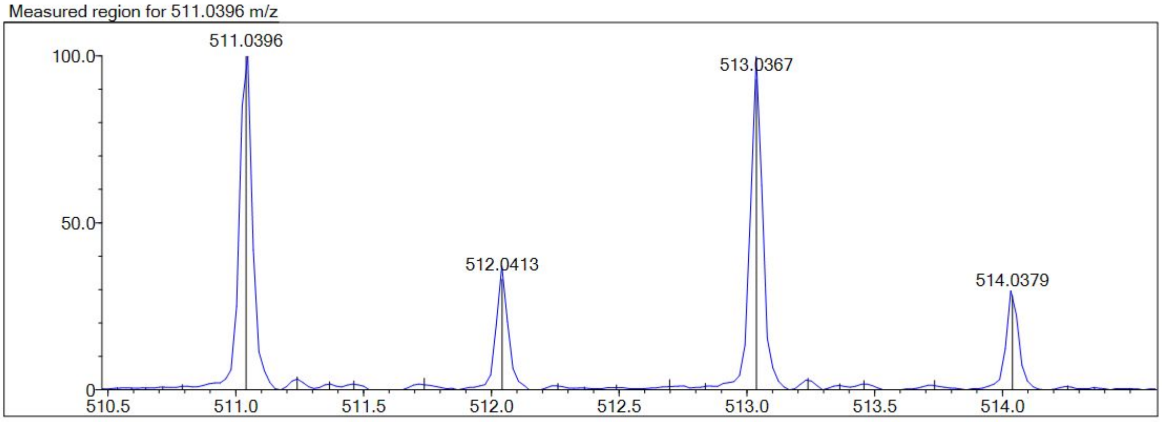

C24 H16 N4 O3 S Cl2 [M+H]+ : Predicted region for 511.0393 m/z

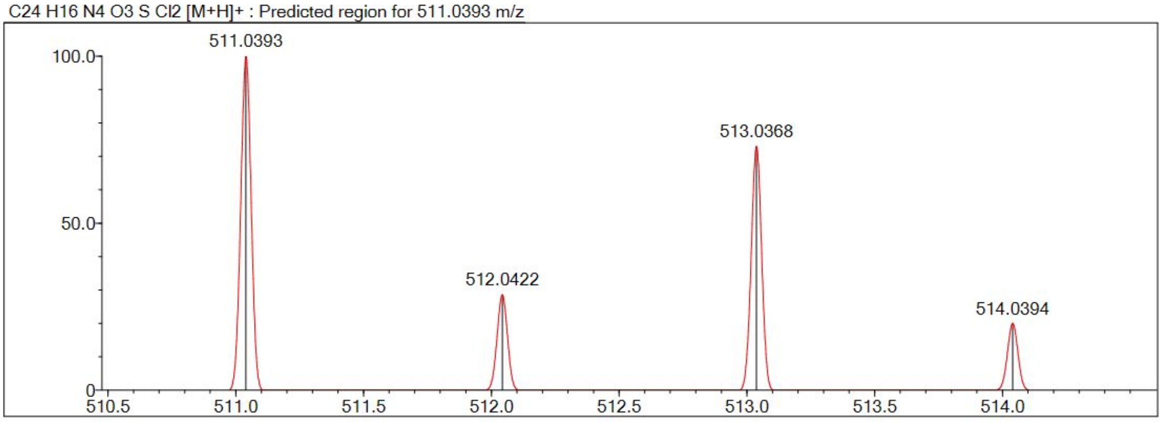

Rank	Score	Formula (M)	Ion	Meas. m/z	Pred. m/z	Df. (mDa)	Df. (ppm)	Iso	DBE
1	57.43	C24 H16 N4 O3 S Cl2	[M+H]+	511.0396	511.0393	0.3	0.59	57.43	18.0

Figure S53. Mass spectrum of compound **4r**

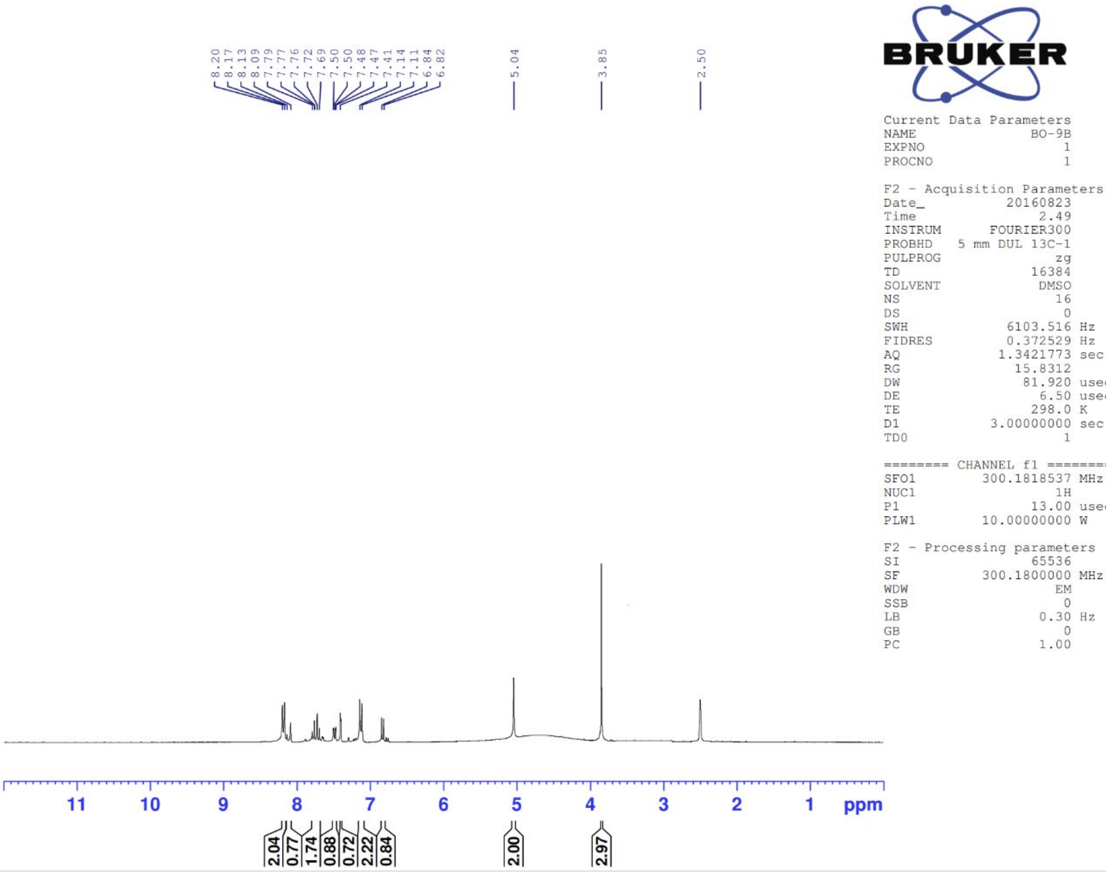

Figure S54. ^{1}H-NMR spectrum of compound **4s**

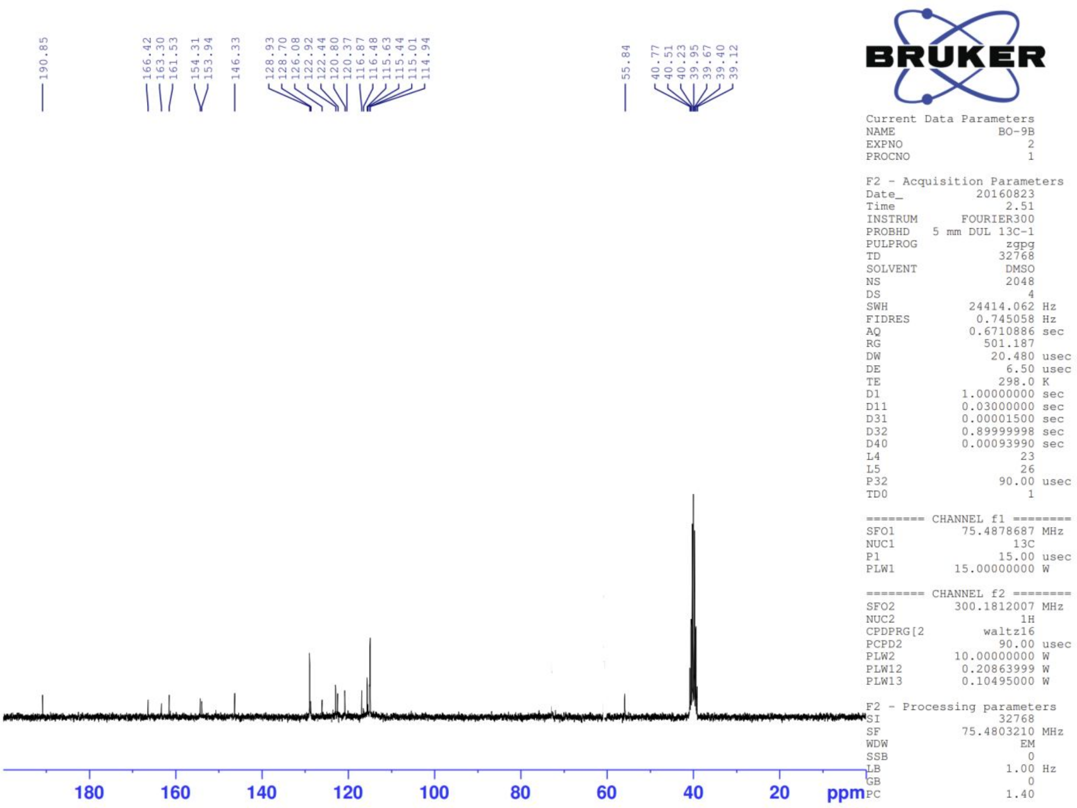

Figure S55. ^{13}C-NMR spectrum of compound **4s**

Formula Predictor Report - BO9-B_7.lcd

Page 1 of 1

Data File: C:\LabSolutions\Data\Analiz\uac\BO9-B_7.lcd

Elmt	Val.	Min	Max	Elmt	Val.	Min	Max	Elmt	Val.	Min	Max	Use Adduct
H	1	10	25	O	2	3	5	Cl	1	0	2	H
C	4	20	29	F	1	0	2	Br	1	0	1	
N	3	4	5	S	2	1	1	I	3	0	0	

Error Margin (ppm):	5	DBE Range:	18.0 - 20.0	Electron Ions:	both
HC Ratio:	unlimited	Apply N Rule:	yes	Use MSn Info:	no
Max Isotopes:	3	Isotope RI (%):	1.00	Isotope Res:	10000
MSn Iso RI (%):	10.00	MSn Logic Mode:	AND	Max Results:	500

Event#: 1 MS(E+) Ret. Time : 5.307 -> 5.307 Scan# : 797 -> 797

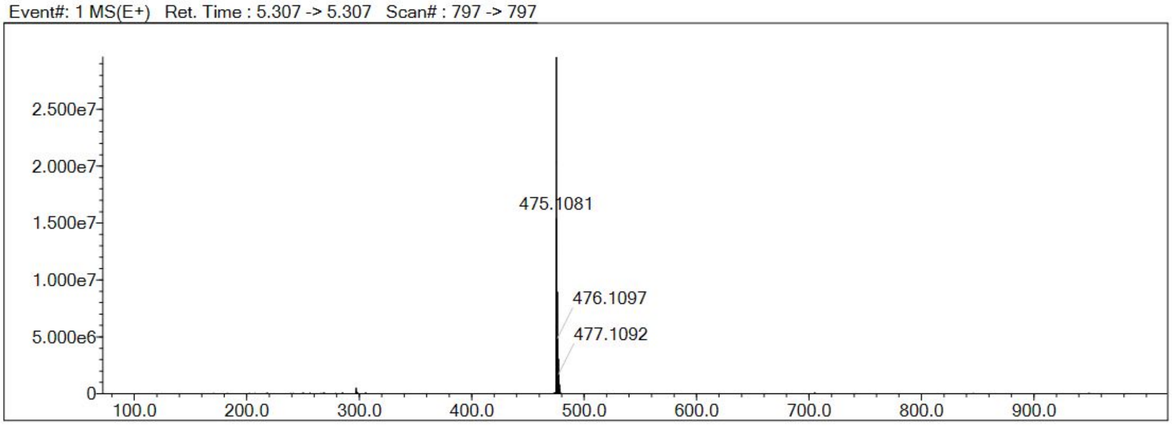

Measured region for 475.1081 m/z

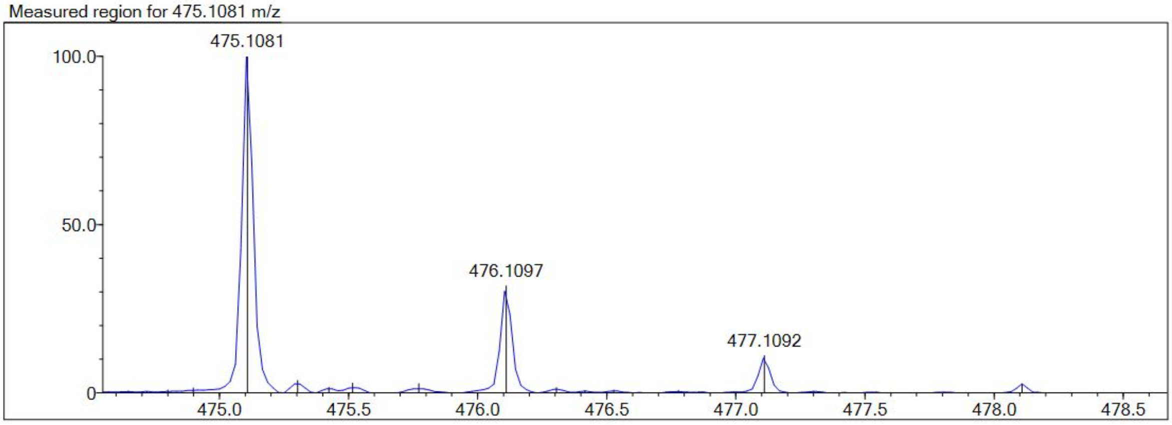

C24 H18 N4 O5 S [M+H]+ : Predicted region for 475.1071 m/z

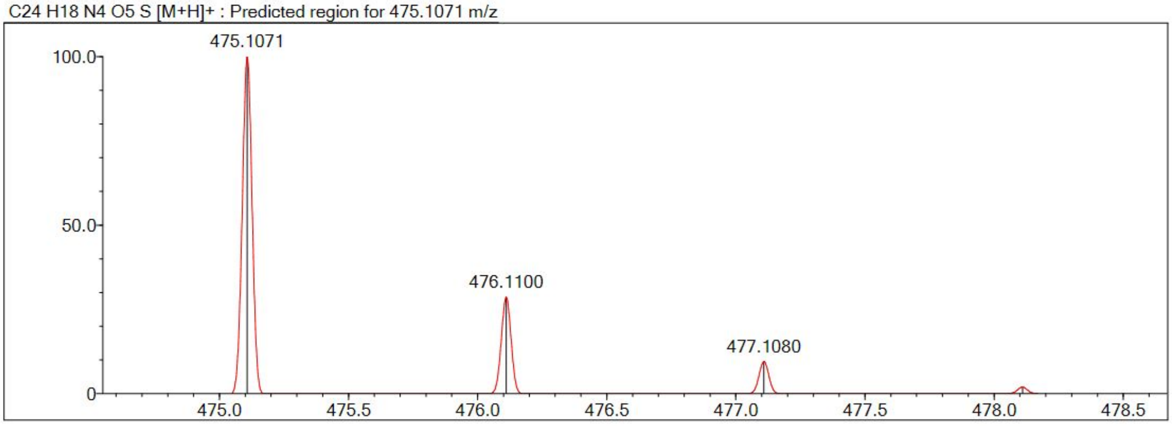

Rank	Score	Formula (M)	Ion	Meas. m/z	Pred. m/z	Df. (mDa)	Df. (ppm)	Iso	DBE
1	91.22	C24 H18 N4 O5 S	[M+H]+	475.1081	475.1071	1.0	2.10	93.80	18.0

Figure S56. Mass spectrum of compound **4s**
